# Co-Occurrence Patterns of Symptom and Functional Burden Associated with Tofacitinib and Upadacitinib: A Multidimensional Analysis of Spontaneous Reports in Rheumatoid Arthritis

**DOI:** 10.3390/pharmaceutics18101318

**Published:** 2026-10-17

**Authors:** Jinglin Yang, Yanyan Wang, Yunfei Ma, Mengyi Lu, Fang Shao, Haichun Liu

**Affiliations:** 1 Jiangsu Key Laboratory of Innovative Drug Research and Development for Mental and Neurological Diseases, China Pharmaceutical University, 639 Longmian Avenue, Nanjing 211198, China; 2 Department of Biostatistics, School of Public Health, Nanjing Medical University, Nanjing 211166, China

**Keywords:** rheumatoid arthritis, tofacitinib, upadacitinib, JAK inhibitors, FAERS, pharmacovigilance, co-occurrence networks, Bayesian networks, time to onset

## Abstract

**Background/Objectives:** Pharmacovigilance studies typically evaluate adverse events as isolated drug–event pairs, potentially overlooking symptoms and functional impairments reported concurrently within the same case. We investigated co-reported adverse event patterns for tofacitinib and upadacitinib in patients with rheumatoid arthritis (RA). **Methods:** FAERS reports submitted between 2004 and 2025 were included if tofacitinib or upadacitinib was recorded as the primary suspect drug for RA. Disproportionality analysis was integrated with three co-occurrence network approaches, Bayesian network analysis, and time-to-onset (TTO) analysis. Methotrexate served as an indication-matched comparator. Network analyses were stratified by reporter and submission type, and TTO was reassessed for the common 2019–2025 period. FAERS signals were also compared descriptively with Canada Vigilance data. **Results:** We identified 57,438 tofacitinib-related and 29,869 upadacitinib-related reports, yielding 316 and 413 positive Preferred Term signals, respectively. In the tofacitinib networks, fatigue was consistently associated with pain, mood-related terms, treatment experience, and gastrointestinal and infectious events. Upadacitinib reports exhibited a distinct pattern, with falls, pain, functional limitations, and injuries occupying central positions. Reduced grip strength, decreased joint range of motion, and loss of personal independence formed a recurrent functional cluster. Methotrexate networks were primarily organized around disease-activity terms. The median reported TTO was 34 days for tofacitinib and 115 days for upadacitinib; corresponding values for the 2019–2025 period were 59 and 115 days, respectively. These intervals should not be interpreted as true onset latency or as established hazard patterns. **Conclusions:** Co-reporting analysis yielded insights beyond individual disproportionality signals. Tofacitinib reports were predominantly characterized by fatigue and associated symptoms, whereas upadacitinib reports more prominently featured functional limitations, falls, and injuries.

## 1. Introduction

Rheumatoid arthritis (RA) is a chronic autoimmune disease characterized by persistent symptom burden and functional impairment [1]. Although treat-to-target strategies and the use of biologic and targeted synthetic disease-modifying antirheumatic drugs have substantially improved disease control, clinical remission does not necessarily restore overall health. Even among patients meeting the Disease Activity Score in 28 Joints (DAS28) remission criteria, pain, fatigue, and functional impairment may persist; these residual symptoms are not fully explained by inflammatory markers or structural damage [2,3,4]. Such burdens often span multiple domains, jointly affecting daily activities, treatment experience, and long-term medication adherence. Consequently, safety assessments should consider patient-reported symptoms and functional changes in addition to serious medical outcomes.

Janus kinase (JAK) inhibitors are widely used to treat RA, yet the long-term safety of tofacitinib and upadacitinib remains under scrutiny. Previous studies have focused primarily on serious infections, herpes zoster, major adverse cardiovascular events, and malignancy [5,6,7,8,9,10,11,12], as well as venous thromboembolism and its reported time to onset [13,14]. While these outcomes form an essential framework for safety evaluation, they do not fully capture the burden experienced during routine treatment. Events such as pain, fatigue, perceived lack of efficacy, functional decline, falls, and treatment interruption may not be classified as serious outcomes, but they can co-occur and affect treatment persistence and daily functioning. Evaluating these events in isolation may obscure the clinical contexts reflected by their joint reporting.

The U.S. Food and Drug Administration (FDA) Adverse Event Reporting System (FAERS) database collects spontaneous reports from patients, health professionals, and manufacturers, providing a resource for examining safety concerns beyond clinical trials. Patient reports can offer insights into symptom experiences, functional effects, and medication use [15]. However, conventional disproportionality analysis treats each drug-event pair as the unit of analysis, primarily assessing whether an individual event is reported more frequently than expected. This approach cannot readily describe how several events combine within the same report, resulting in disconnected signals that do not directly identify potentially interrelated reporting patterns [16,17].

Adverse-event co-occurrence networks provide a complementary perspective by representing events as connected nodes and identifying closely associated clusters at the report level [18]. Bayesian networks further elucidate conditional associations among co-reported events, while time-to-onset (TTO) analysis describes when reported events occur after treatment initiation. Used alongside conventional signal detection, these methods allow for the simultaneous consideration of signal strength, event structure, and reporting time. Real-world studies of JAK inhibitors have largely focused on individual serious outcomes; systematic comparisons of event combinations, central nodes, and temporal patterns for tofacitinib and upadacitinib remain limited.

We therefore combined conventional disproportionality analysis with co-occurrence networks, Bayesian networks, and TTO analysis to characterize reporting patterns for tofacitinib and upadacitinib in FAERS reports related to RA. The Canada Vigilance Adverse Reaction Online Database (CVARD) served as a complementary national reporting source for a descriptive comparison of preferred-term signals. This approach aimed to identify drug-specific event clusters and central nodes that may be missed by single-event signal detection.

## 2. Materials and Methods

### 2.1. Data Collection and Preprocessing

**Figure 1:**
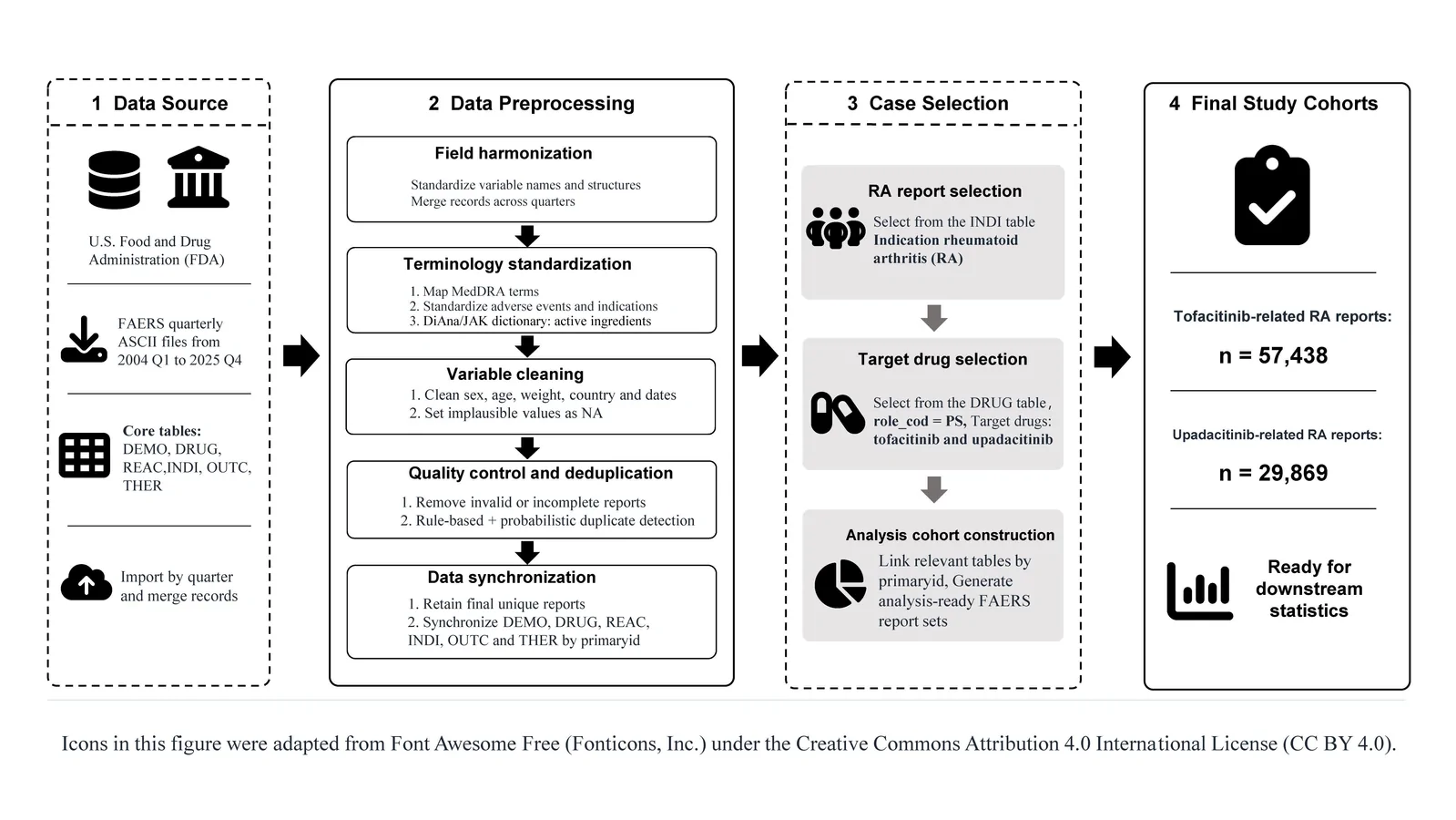
FAERS data-cleaning workflow. Data processing included downloading, preprocessing, case selection, and construction of the final analytic datasets. FAERS, U.S. Food and Drug Administration Adverse Event Reporting System.

Quarterly data extracts from the FDA FAERS, released between 2004 and 2025, were utilized. Demographic and Administrative Data (DEMO), Drug Information (DRUG), Adverse Events (REAC), Patient Outcomes (OUTC), Report Sources (RPSR), Therapy Dates (THER), and Indications (INDI) were linked via report identifier. This relational structure aligns with standardized representations of spontaneous adverse-event reporting data [19]. Drug names were mapped to standardized active ingredients using the DiAna dictionary [20], and adverse events were coded to Medical Dictionary for Regulatory Activities (MedDRA) version 28.1 Preferred Terms (PTs). Reports were included if tofacitinib or upadacitinib was recorded as the primary suspect drug in the DRUG table (ROLE_COD = “PS”) and at least one INDI record linked by report identifier (PRIMARYID) contained the MedDRA preferred term “rheumatoid arthritis” in the INDI_PT field. These selection criteria were applied uniformly to both drugs. Data cleaning followed the DiAna workflow. For cases with multiple versions, the record with the latest FDA receipt date was retained. Deduplication was performed on the entire DEMO table prior to cohort selection using duplicate flags generated by the DiAna workflow. Of the 19,541,117 DEMO records, 3,503,741 (17.9%) were flagged as duplicates and removed, leaving 16,037,376 records for cohort construction. The final dataset comprised 57,438 tofacitinib-related and 29,869 upadacitinib-related reports. Figure 1 illustrates the selection process [21,22].

Additionally, CVARD served as a complementary data source for a descriptive cross-database comparison. Reports recorded between 1 January 2012 and 31 December 2025 were restricted to those listing tofacitinib or upadacitinib as a suspect drug and rheumatoid arthritis as the indication. Drug names and adverse events were standardized to active ingredients and MedDRA PTs, respectively, and the most recent version of duplicate or follow-up reports was retained. The same composite positive-signal criterion defined for FAERS was applied to CVARD. Further details regarding CVARD processing and cohort characteristics are provided in the Supplementary Methods and Table S15.

### 2.2. Signal Detection

Signals were detected using the reporting odds ratio (ROR), proportional reporting ratio (PRR), Bayesian confidence propagation neural network (BCPNN), and multi-item gamma Poisson shrinker (MGPS) [23,24,25]. All disproportionality estimates were calculated within the rheumatoid arthritis reporting background; thus, the reference group for each drug consisted of all other reports listing rheumatoid arthritis as the indication, rather than the full database. Consequently, manifestations of the underlying disease appear in both the exposed and reference groups and cannot independently generate a positive signal. For the primary analysis, PTs with at least three reports were evaluated. A positive signal required the lower 95% credible limit of the information component (IC025) > 0 and at least one of the following criteria: (1) a lower 95% confidence limit of the ROR > 1; (2) PRR ≥ 2 and χ^2^ ≥ 4; or (3) the lower 90% credible limit of the empirical Bayes geometric mean (EBGM05) > 2. Under the WHO-UMC BCPNN framework, signals are operationally defined using the criterion IC025 > 0. This Bayesian framework employs shrinkage to stabilize estimates under sparse data conditions, thereby reducing the impact of statistical noise relative to purely unsmoothed disproportionality measures. The supporting criteria were combined using a disjunctive rule, such that a signal was identified if the PT met any of the predefined criteria. A two-fold effect-size requirement was deliberately omitted because symptom- and functional-event PTs are common in RA regardless of treatment, and their relative reporting increases are expected to be modest. As a signal-level sensitivity analysis, PTs that were positive under each criterion applied independently (BCPNN/IC025, ROR with a lower 95% confidence limit > 1, PRR ≥ 2 with χ^2^ ≥ 4, and EBGM05 > 2) were re-screened. The composite analysis was then repeated using minimum report-count thresholds of 10 and 30 (Supplementary Tables S30 and S31). Signals were classified as listed or unlisted by comparison with the latest FDA prescribing information for each drug. The 2 × 2 contingency table, detailed formulae, and positive-signal criteria are provided in Supplementary Tables S1 and S2 [26,27,28].

### 2.3. Co-Occurrence Network Analysis

Following signal detection, PTs meeting the combined positive-signal criteria were considered candidate network nodes [18]. Frequency thresholds were applied to limit the inclusion of sparsely reported PTs in the networks. Because the upadacitinib cohort contained approximately half as many reports as the tofacitinib cohort, a lower relative-frequency threshold was used to retain a broader set of events in the smaller cohort. PTs were retained if they occurred in at least 1 per 200 tofacitinib reports or 1 per 300 upadacitinib reports, corresponding to minimum counts of 288 and 100, respectively. The methotrexate comparator used the same minimum count of 288 reports as tofacitinib. These filters yielded similarly sized node sets of 77, 65, and 79 PTs for tofacitinib, upadacitinib, and methotrexate, respectively, thereby limiting disparities in network size during descriptive comparisons. These pragmatic thresholds applied only to network construction and did not affect disproportionality signal detection.

**Figure 2:**
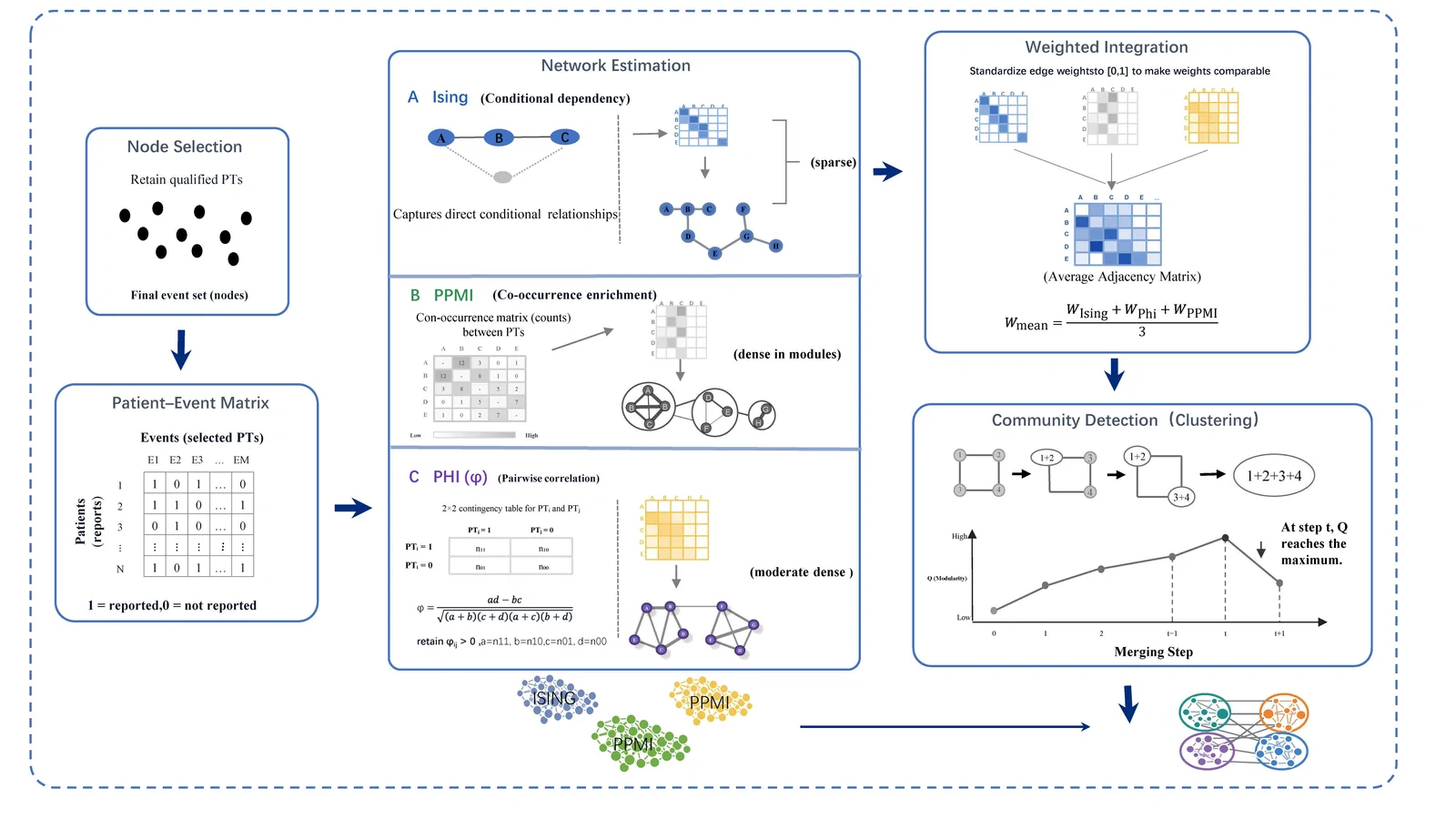
Co-occurrence network analysis workflow. Positive PTs formed a binary report-by-event matrix. Associations and clusters were estimated independently using the Ising model, PPMI, and the phi coefficient and were presented as complementary network representations.

Using the same drug-specific node set, event associations were independently estimated using the Ising model, positive pointwise mutual information (PPMI), and the phi coefficient. The Ising model estimates conditional dependencies through least absolute shrinkage and selection operator (LASSO) regularization, producing a sparse network [29,30,31]. PPMI quantifies positive co-occurrence above that expected under independence, with additive smoothing reducing instability arising from infrequent event pairs [32]. The phi coefficient measures the overall association between binary events and is more sensitive to co-occurrence among relatively common events [33]. Supplementary Table S3 provides detailed formulae and implementation parameters. The overall workflow of the co-occurrence network analysis is shown in Figure 2.

Communities were identified separately using fast-greedy modularity optimization. Network structure and cross-method agreement were summarized using weighted modularity, small-worldness, edge-set Jaccard similarity, and symmetric mean community purity, as detailed in Supplementary Table S4.

Co-occurrence networks were additionally re-estimated within the health-professional, consumer, expedited, and non-expedited strata of each drug. The node set was held fixed at the main-analysis node set to ensure that degree z-scores and edge-set similarities remained comparable across strata. The Jaccard index measures the proportion of shared edges in the union of two edge sets, with higher values indicating greater edge overlap. Purity measures the agreement between community assignments, with higher values indicating greater concordance, although it is sensitive to the number and size of communities. Both indices range from 0 to 1, with values approaching 1 indicating identical edge sets or community assignments and values approaching 0 indicating no correspondence. As no consensus benchmark exists for adverse-event co-occurrence networks, both indices were used for relative, descriptive comparison across methods rather than as pass/fail criteria (Table S4).

### 2.4. Sensitivity Analysis Using a Methotrexate Comparator Cohort

As a sensitivity analysis for RA-related reporting, PT signal and co-occurrence network analyses were repeated using methotrexate as an RA comparator. Methotrexate was selected because it is the anchor drug for rheumatoid arthritis, shares the indication and much of the treated population, and acts through a mechanism unrelated to JAK inhibition. Therefore, a configuration generated by the disease should appear in the methotrexate cohort, whereas one specific to JAK inhibition should not. Reports were included when methotrexate was listed as the primary suspect drug and rheumatoid arthritis was recorded as the indication. The same observation period, data-cleaning procedures, deduplication rules, PT selection, and composite positive-signal criterion were applied as in the primary analyses. A positive signal required at least three reports, IC025 > 0, and at least one of the following: ROR025 > 1, PRR ≥ 2 with χ^2^ ≥ 4, or EBGM05 > 2. The final methotrexate comparator cohort comprised 37,526 unique reports. Details of the comparator construction and density-corrected centrality analysis are provided in Supplementary Methods.

### 2.5. Bayesian Network Analysis

To characterize conditional-association structures among co-reported adverse events, Bayesian networks were constructed using the same drug-specific PT node sets as the co-occurrence networks [34,35]. The resulting directed acyclic graphs represented model-derived orientations of conditional associations. Because FAERS records co-reporting information without event-level temporal ordering, clinical interpretation focused on the learned connectivity structure.

**Figure 3:**
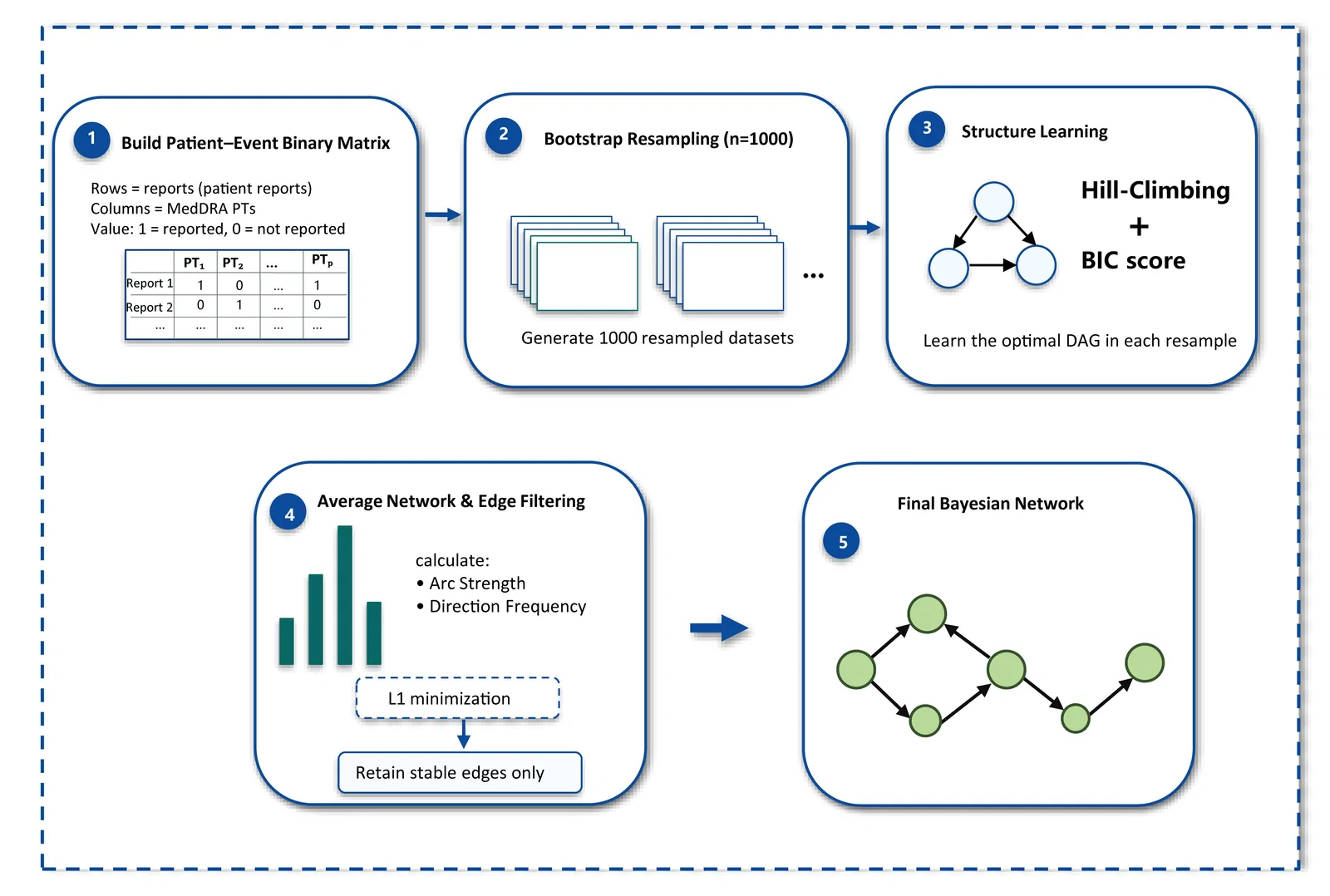
Bayesian network analysis workflow. Candidate directed acyclic graphs were learned using a score-based hill-climbing algorithm, assessed by bootstrap resampling, and averaged after applying the selected edge-strength threshold. Directed edges indicate model-derived orientations of conditional dependence.

Candidate graphs were learned using a score-based hill-climbing algorithm, with the Bayesian information criterion balancing model fit and network complexity [35]. Structural stability was assessed using 1000 bootstrap resamples. An averaged network was constructed using the edge-strength threshold selected by L1-norm minimization, and bootstrap edge-inclusion frequency and orientation stability were recorded for each retained edge [36]. To quantify complementarity with the co-occurrence networks, Bayesian edges were collapsed into undirected pairs and compared with the Ising, phi, and PPMI edge sets using the Jaccard index. Network interpretation focused on node connectivity, betweenness, and stable adjacencies. The workflow of the Bayesian network analysis is shown in Figure 3.

### 2.6. Time-to-Onset Analysis

Time-to-onset (TTO) was defined as the interval in days between the treatment start date (START_DT) and the adverse-event date (EVENT_DT), with analyses restricted to 0–365 days. Reports with missing or incomplete dates, an EVENT_DT earlier than START_DT, or a calculated TTO exceeding 365 days were excluded [37,38]. Across all reports for each drug over the full observation period, 3112 of 57,438 tofacitinib reports (5.42%) and 2912 of 29,869 upadacitinib reports (9.75%) had a calculated TTO exceeding 365 days and no eligible TTO within 0–365 days. Overall TTO analyses included reports containing at least one positive PT, with each report (PRIMARYID) contributing its earliest eligible TTO (Supplementary Table S26). TTO distributions were summarized using medians, quartiles, and ranges. Weibull scale (α) and shape (β) parameters were estimated with 95% confidence intervals. Failure-type classification was based on the unrounded 95% confidence interval of β: an interval entirely below 1 indicated an early-failure pattern, an interval containing 1 indicated a random-failure pattern, and an interval entirely above 1 indicated a wear-out pattern. The β-based labels describe the shape of the fitted distribution of reported intervals. Because FAERS captures report-level timing rather than population-based follow-up, β is interpreted as a descriptor of the reported-interval distribution: a value below 1 denotes a greater concentration of recorded intervals near treatment initiation with a longer fitted right tail, rather than an estimate of biological hazard. Same-day observations were retained as 0 days in descriptive summaries and replaced with 0.5 days only for Weibull fitting [39,40,41,42]. PT-specific Weibull analyses required at least three eligible TTO observations; PTs with fewer observations were excluded. For each PT, the proportion contributing to the analysis was calculated as the number of reports with an eligible TTO divided by the total number of reports containing that PT for the same drug and observation period. Kaplan–Meier curves illustrated cumulative TTO distributions, and the Kruskal–Wallis test compared TTO distributions across positive PTs with at least three eligible observations.

To address differences in drug approval dates, analyses were repeated within a common 2019–2025 calendar window, with positive PT signals re-estimated within that window. Calendar year was assigned using the event date when available, followed by the initial FDA receipt date and then the FDA receipt date. Among positive-signal reports in this window, 1079 of 22,687 tofacitinib reports (4.76%) and 1873 of 17,866 upadacitinib reports (10.48%) were excluded for TTO exceeding 365 days (Supplementary Table S26). The proportion of reports with an eligible TTO and the characteristics of included and excluded reports were assessed by drug, reporter type, seriousness status, and reporting year. TTO distributions were additionally compared between drugs within strata defined by reporter type, submission type, and seriousness status using the Wilcoxon rank-sum test.

### 2.7. Statistical Analysis

Data were organized in Microsoft Excel 2019. Signal detection, network analysis, Bayesian network analysis, TTO analysis, and visualization were conducted in R version 4.5.1. Differences in TTO distributions across positive PTs were assessed using the Kruskal–Wallis test, with *p* < 0.05 considered statistically significant.

## 3. Results

### 3.1. Baseline Characteristics of Adverse-Event Reports

**Figure 4:**
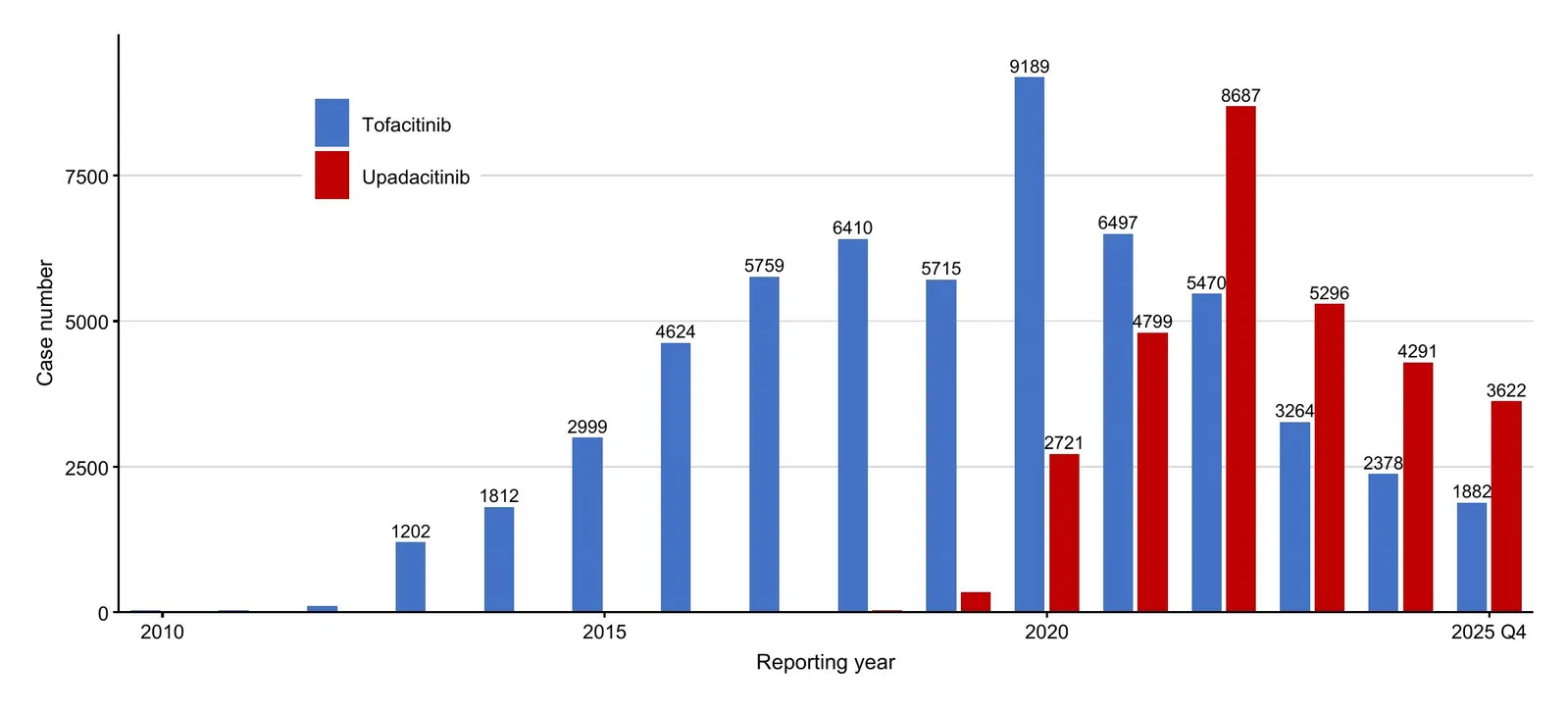
Annual trends in adverse-event reports involving tofacitinib and upadacitinib among patients with RA in FAERS.

Following data cleaning and deduplication, 57,438 tofacitinib-related and 29,869 upadacitinib-related RA reports were included. Percentages for sex, reporter type, and outcome were calculated using the total number of reports in each drug cohort, including those with unknown values, as the denominator. Women accounted for 81.7% of tofacitinib reports and 78.3% of upadacitinib reports. Tofacitinib reports were submitted in similar proportions by health professionals (43.7%) and consumers (41.9%), whereas consumers submitted 72.5% of upadacitinib reports. Hospitalization was recorded in 14.4% of tofacitinib reports and 24.6% of upadacitinib reports, while other serious outcomes were noted in 25.8% and 39.9%, respectively. The majority of reports originated from the Americas (93.8% for tofacitinib and 88.7% for upadacitinib) (Table 1; Figure 4).

Reporter-stratified baseline characteristics are presented in Supplementary Table S21. The proportion of expedited reports was higher for upadacitinib than for tofacitinib among both health-professional reports (70.6% versus 40.0%) and consumer reports (78.9% versus 41.4%). Serious reports were also more frequent in the upadacitinib strata than in the corresponding tofacitinib strata.

### 3.2. Signal Detection

The primary composite definition identified 316 positive PT signals for tofacitinib, comprising 22 listed and 294 unlisted signals. Criterion-specific analyses identified 316 PTs meeting IC025 > 0, 368 meeting the ROR criterion, 257 meeting the proportional reporting ratio/chi-squared (PRR/χ^2^) criterion, and 57 meeting the EBGM05 criterion. As these criteria overlap, the criterion-specific counts are not additive. Every PT meeting IC025 > 0 also satisfied at least one supporting criterion; consequently, the composite definition selected the same 316 PTs as IC025 > 0 applied alone and was more restrictive than the ROR criterion applied alone. The median report count among the 316 primary positive PTs was 66.5 (interquartile range [IQR], 21–285; range, 3–7360). When the minimum report count threshold was increased to 10 and 30, 283 and 214 positive PTs remained, respectively.

**Figure 5:**
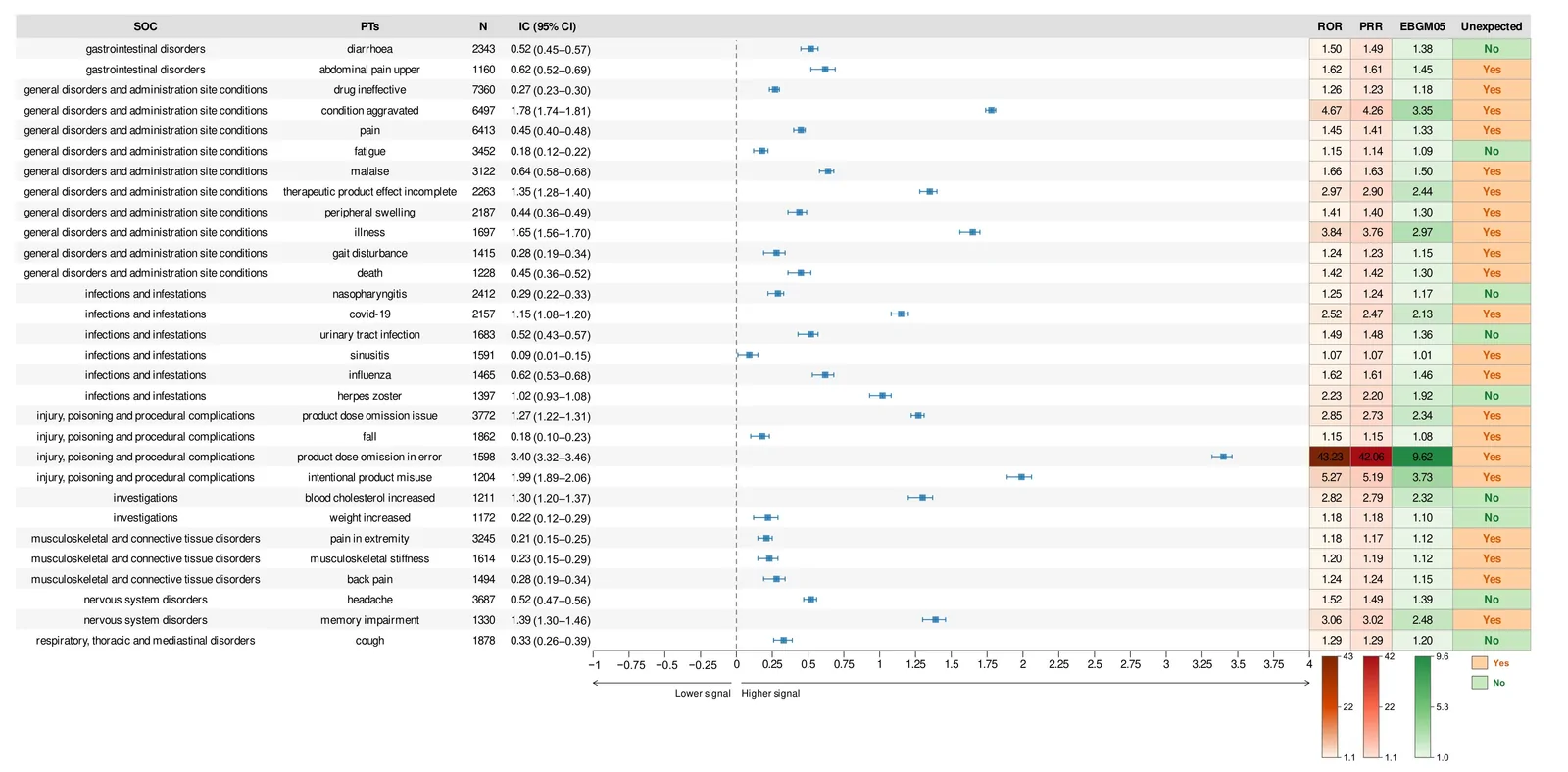
Disproportionality signals for adverse events reported with tofacitinib.

Among PTs meeting the positive-signal definition for tofacitinib, those with the highest report counts were drug ineffective (n = 7360; IC025 = 0.23), condition aggravated (n = 6497; IC025 = 1.74), pain (n = 6413; IC025 = 0.40), product dose omission issue (n = 3772; IC025 = 1.22), and headache (n = 3687; IC025 = 0.47) (Figure 5; Supplementary Table S18).

**Figure 6:**
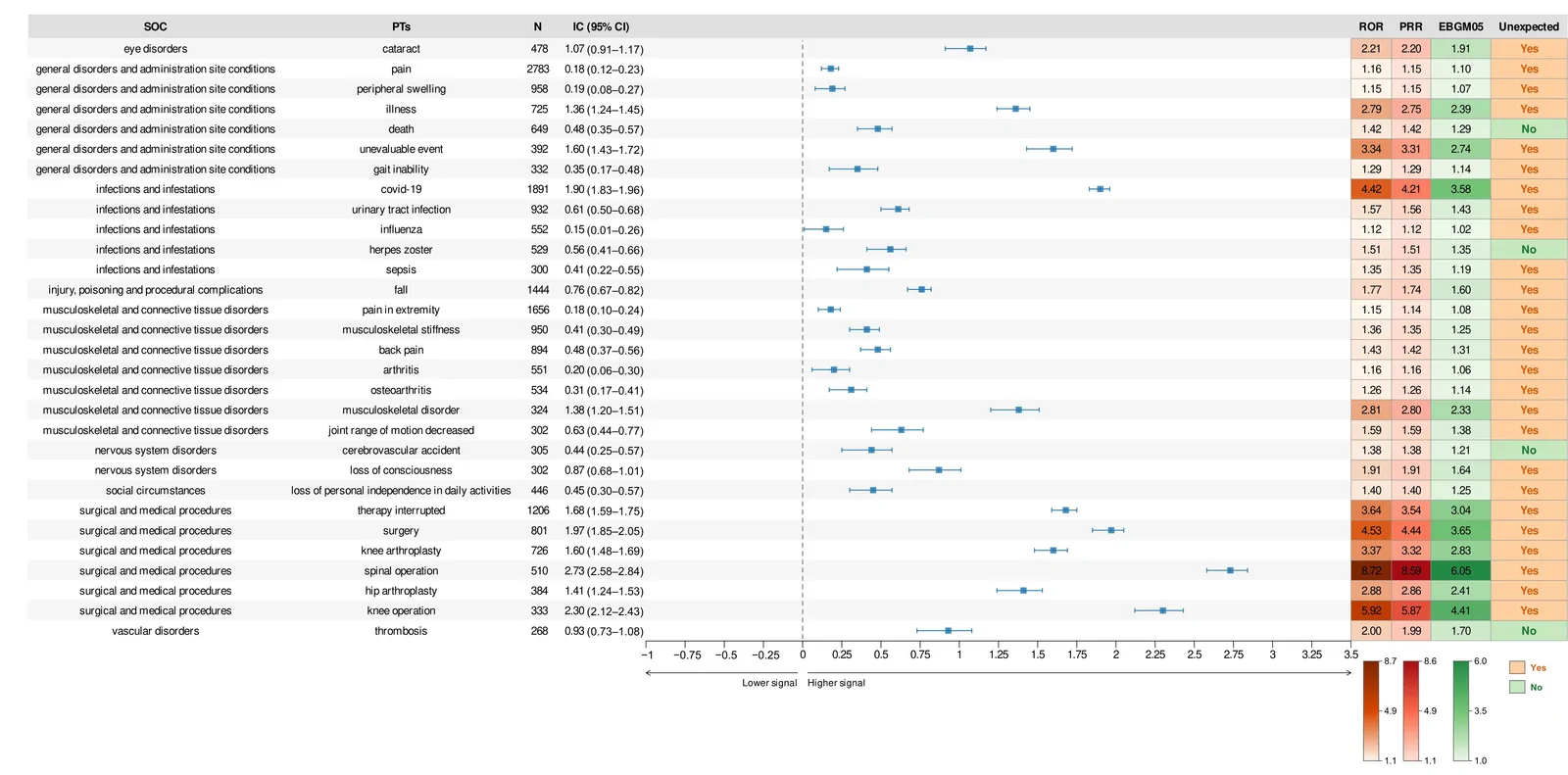
Disproportionality signals for adverse events reported with upadacitinib.

For upadacitinib, the primary composite definition identified 413 positive PT signals, including 13 listed and 400 unlisted signals. Criterion-specific analyses identified 413 PTs meeting IC025 > 0, 515 meeting the ROR criterion, 498 meeting the PRR/χ^2^ criterion, and 145 meeting the EBGM05 criterion. The composite definition again coincided with IC025 > 0 and was more restrictive than either the ROR or the PRR/χ^2^ criterion applied alone. The median report count among the 413 primary positive PTs was 17 (IQR, 7–53; range, 3–2783). With minimum report-count thresholds of 10 and 30, 275 and 149 positive PTs remained, respectively. Thus, the upadacitinib signal set contained a larger low-frequency component than the tofacitinib signal set. Low-count PTs identified in the disproportionality analysis were interpreted as exploratory signals, whereas the network analyses were restricted to higher-frequency positive PTs reported in at least 288 tofacitinib reports or 100 upadacitinib reports. Among PTs meeting the positive-signal definition for upadacitinib, those with the highest report counts were pain (n = 2783; ROR = 1.16, 95% CI 1.11–1.20; IC025 = 0.12), COVID-19 (n = 1891; ROR = 4.42, 95% CI 4.20–4.65; IC025 = 1.83), pain in extremity (n = 1656; ROR = 1.15, 95% CI 1.09–1.21; IC025 = 0.10), fall (n = 1444; ROR = 1.77, 95% CI 1.68–1.87; IC025 = 0.67), and therapy interrupted (n = 1206; ROR = 3.64, 95% CI 3.42–3.87; IC025 = 1.59) (Figure 6; Supplementary Table S19). Complete signal estimates are provided in Supplementary Tables S18 and S19. Arthralgia did not meet the positive-signal criterion for either drug (4392 tofacitinib and 2418 upadacitinib reports), and fatigue did not meet it for upadacitinib (1023 reports). Given the large counts, the absence of a signal reflects the absence of disproportionate reporting relative to other rheumatoid arthritis reports rather than limited precision.

CVARD included 15,648 tofacitinib-related reports and 4985 upadacitinib-related reports. Reporter composition differed between the two cohorts: health professionals accounted for 69.7% of tofacitinib-related reports and 9.4% of upadacitinib-related reports, whereas consumers accounted for 28.7% and 45.9%, respectively; the corresponding proportions with unknown reporter type were 1.5% and 44.7% (Table S15). CVARD yielded 557 positive tofacitinib PT signals and 101 positive upadacitinib PT signals. Among the FAERS-positive PTs identified in the main analysis, 129 of 316 tofacitinib signals (40.8%) and 43 of 413 upadacitinib signals (10.4%) were also positive in CVARD under the same composite signal criterion. When considered relative to the CVARD signal sets, the overlaps were 129 of 557 (23.2%) and 43 of 101 (42.6%). Because an overlap cannot exceed the size of the smaller signal set, the 101 positive upadacitinib PTs identified in CVARD capped the attainable overlap at 24.5% of the 413 FAERS signals; relative to this ceiling, the two drugs reached a similar fraction (40.8% and 42.6%). Restricting the comparison to PTs for which CVARD had sufficient reports to perform disproportionality analysis, the overlap was 129 of 248 (52.0%) for tofacitinib and 43 of 143 (30.1%) for upadacitinib. Therefore, the overlap reflects the size and information density of the two CVARD cohorts as much as any drug-specific difference (Supplementary Tables S16–S19; Supplementary Figure S6).

### 3.3. Co-Occurrence Network Analysis

**Figure 7:**
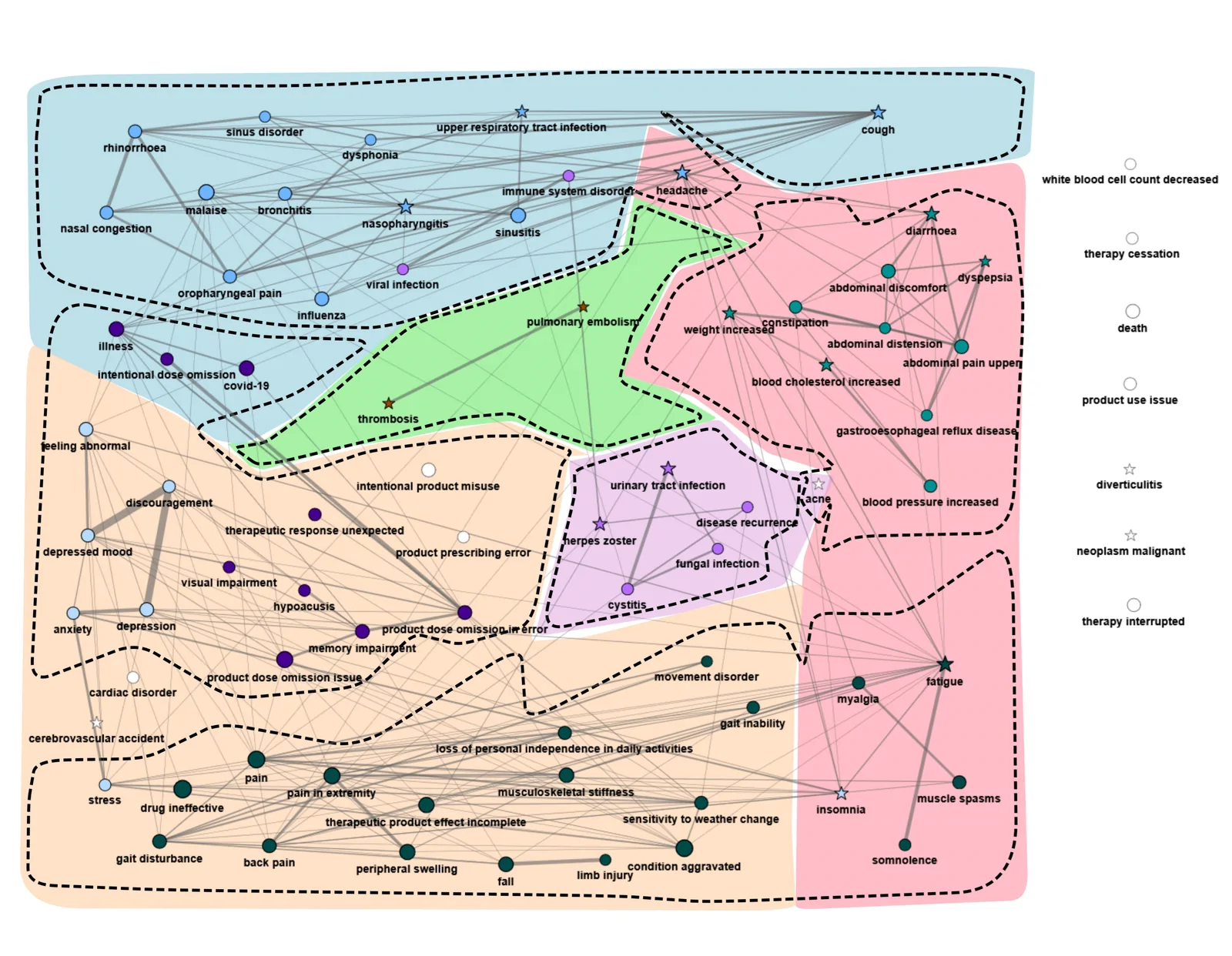
Tofacitinib adverse-event co-occurrence networks. Edge width represents association strength; node colors, dashed outlines, and solid outlines indicate clusters identified by the Ising, phi, and PPMI methods, respectively. Node positions were manually adjusted to reduce overlap. Asterisks indicate PTs corresponding to adverse reactions or symptoms listed in the current prescribing information for tofacitinib.

**Figure 8:**
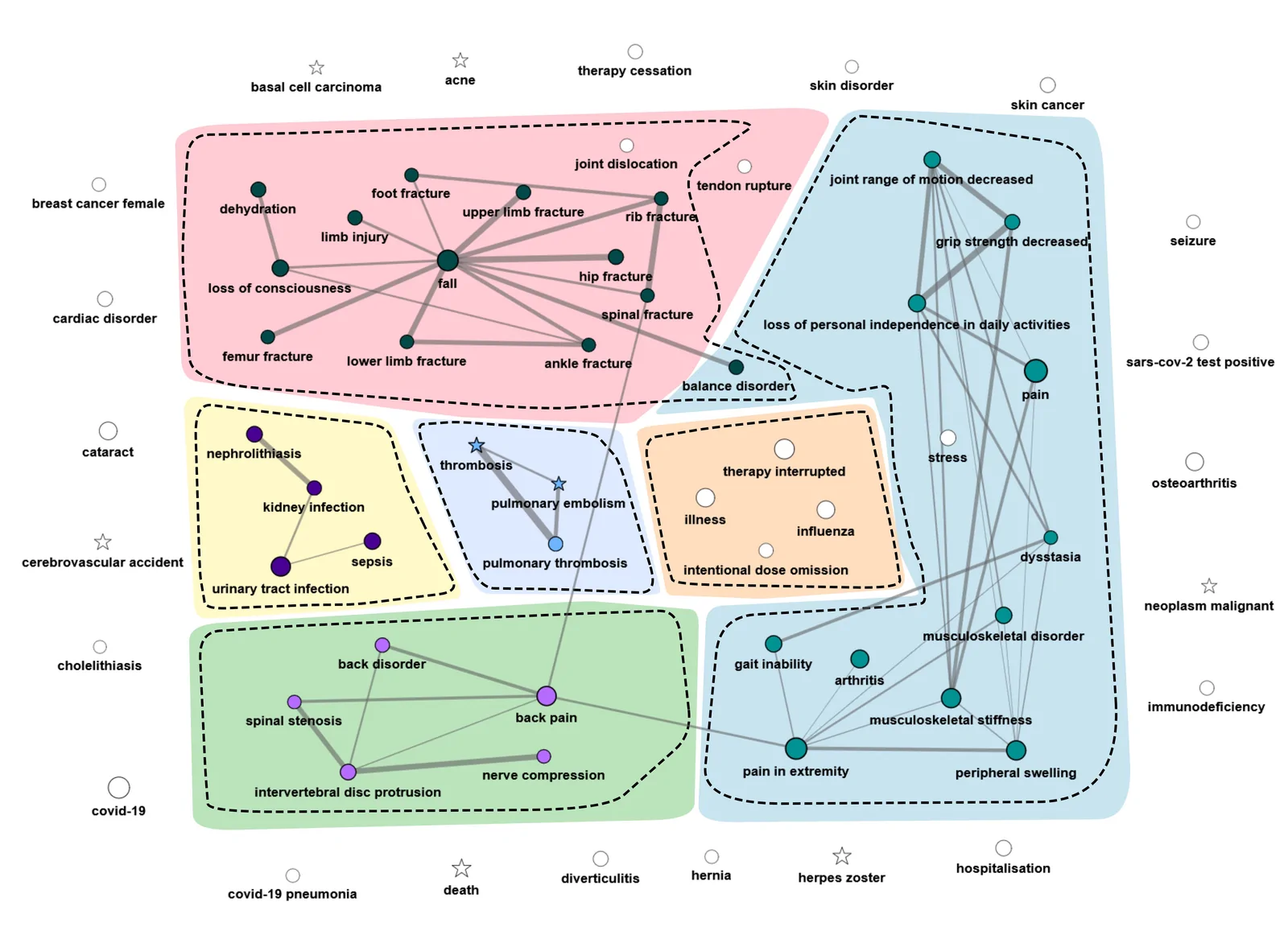
Upadacitinib adverse-event co-occurrence networks. Edge width represents association strength; node colors, dashed outlines, and solid outlines indicate clusters identified by the Ising, phi, and PPMI methods, respectively. Node positions were manually adjusted to reduce overlap. Asterisks indicate PTs corresponding to adverse reactions or symptoms listed in the current prescribing information for upadacitinib.

All six networks exhibited modularity values above 0.3. Edge overlap was greatest between the phi and PPMI networks: the two methods shared 74.1% of the edges in their union for tofacitinib and 83.0% for upadacitinib (Jaccard indices, 0.741 and 0.830, respectively). Symmetric mean community purity ranged from 0.766 to 0.916 for tofacitinib and from 0.938 to 0.969 for upadacitinib, with values closer to 1 indicating closer correspondence between community assignments (Table 2 and Table 3; Supplementary Table S4). The sparse Ising networks emphasized local conditional dependencies, whereas the phi and PPMI networks retained broader association patterns (Table 2 and Table 3; Figure 7 and Figure 8; Supplementary Tables S5–S10).

The tofacitinib Ising, phi, and PPMI networks shared 77 nodes. Fatigue was the highest-degree node in every representation, with degrees of 21, 30, and 40. Pain, back pain, pain in extremity, insomnia, illness, headache, and condition aggravated also ranked among the most connected PTs. Five domains were reproduced by all three estimators: psychological symptoms, gastrointestinal symptoms, respiratory or infectious events, recurrent or herpes-zoster-related infections, and thromboembolic events. The psychological cluster was the most stable and consistently contained depressed mood, depression, discouragement, anxiety, feeling abnormal, insomnia, and stress, with discouragement linked to both depressed mood and depression in every network; the depressed mood–discouragement edge was consistently the stronger of the two. The respiratory/infectious, recurrent-infection, and thromboembolic clusters likewise retained closely related PT cores throughout. Method-specific differences involved the broader symptom and medication-use clusters: in the PPMI network the psychological structure extended toward fatigue, myalgia, somnolence, and memory impairment, indicating co-occurrence across mood, sleep, cognitive, and general symptom domains, whereas in the Ising and phi networks these PTs were assigned to separate communities. Musculoskeletal and functional-burden and medication-use communities were also delineated, but their boundaries varied across methods. (Figure 7; Supplementary Tables S5–S7).

The upadacitinib networks shared 65 nodes and were centered on functional burden and injury-related reporting. Fall was the highest-degree node in the Ising and phi networks, whereas pain was co-central with fall, back pain, joint range of motion decreased, and pain in extremity in the PPMI network. Five domains were reproduced by all three estimators: functional burden, falls and fractures, urinary or infectious events, spinal disorders, and thromboembolic events. The functional-burden core consistently included grip strength decreased, joint range of motion decreased, loss of personal independence in daily activities, musculoskeletal stiffness, dysstasia, and pain. Fall was consistently connected with hip, upper-limb, lower-limb, femur, rib, and spinal fractures. Method-specific differences involved the community assignments of peripheral terms, including balance disorder and stress, and the grouping of illness, influenza, intentional product dose omission, and therapy interrupted (Figure 8; Supplementary Tables S8–S10).

Across reporter and submission-type strata, fatigue or pain remained the most connected node in every tofacitinib network (edge Jaccard 0.485–0.647; community purity 0.73–0.90), and fall remained the most connected node in the upadacitinib consumer and expedited networks, which cover 21,645 and 20,881 of the 29,869 reports (Jaccard 0.670–0.839; purity 0.92–1.00). In the two smallest upadacitinib strata, the networks were too sparse to identify a single central node; however, pain, pain in extremity, fall, femur fracture, and loss of personal independence in daily activities still occupied degree percentiles of 92.6 to 99.2 in the health-professional stratum (Table S33). The functional domain therefore persists across strata. The networks for the two drugs remained distinguishable within every stratum (cross-drug edge Jaccard 0.118–0.442; Table S34).

### 3.4. Sensitivity Analysis Using a Methotrexate Comparator Cohort

As a sensitivity analysis for RA-related reporting, we repeated the PT signal and co-occurrence network analyses in 37,526 methotrexate reports, using the same RA background and the same composite criterion. The comparator analysis identified 1263 positive PT entries and retained 79 candidate network nodes, the least frequent of which was reported 294 times. These networks were organized around disease-activity terms, including arthritis, arthropathy, synovitis, joint swelling, musculoskeletal stiffness, and abnormalities of C-reactive protein and erythrocyte sedimentation rate. Pain and fall did not meet the positive-signal criterion in the comparator cohort and were not retained as nodes, whereas fatigue and loss of personal independence in daily activities did meet the criterion and were retained, although in less consistent network positions (Table 4). Complete comparator results are provided in Supplementary Table S32.

### 3.5. Bayesian Network Analysis

The Bayesian networks showed partial edge-set overlap with the co-occurrence networks. The Jaccard indices for Bayesian versus Ising, phi, and PPMI networks were 0.430, 0.316, and 0.239 for tofacitinib, and 0.351, 0.310, and 0.283 for upadacitinib, respectively. Thirty-three of 160 tofacitinib Bayesian edges and 55 of 100 upadacitinib Bayesian edges were absent from the union of the three co-occurrence networks. These Bayesian-specific edges had median bootstrap inclusion frequencies of 0.853 and 0.855, respectively (Supplementary Tables S28 and S29). Bootstrap resampling showed that the presence of an adjacency was considerably more reproducible than its orientation. The median edge-inclusion frequency was 0.921 for tofacitinib and 0.909 for upadacitinib, and 54.4% and 51.0% of retained edges, respectively, were recovered in at least 90% of resamples. Orientation was less stable. The median orientation stability was 0.855 for tofacitinib and 0.895 for upadacitinib, but it fell below 0.80 for 42.5% and 30.0% of edges and below 0.60 for 15.6% and 12.0%. Edges with orientation stability close to 0.50 carry essentially no directional information; for these edges, only the adjacency can be regarded as supported. Edge-inclusion and orientation frequencies for the adjacencies discussed above are given in Table S29.

**Figure 9:**
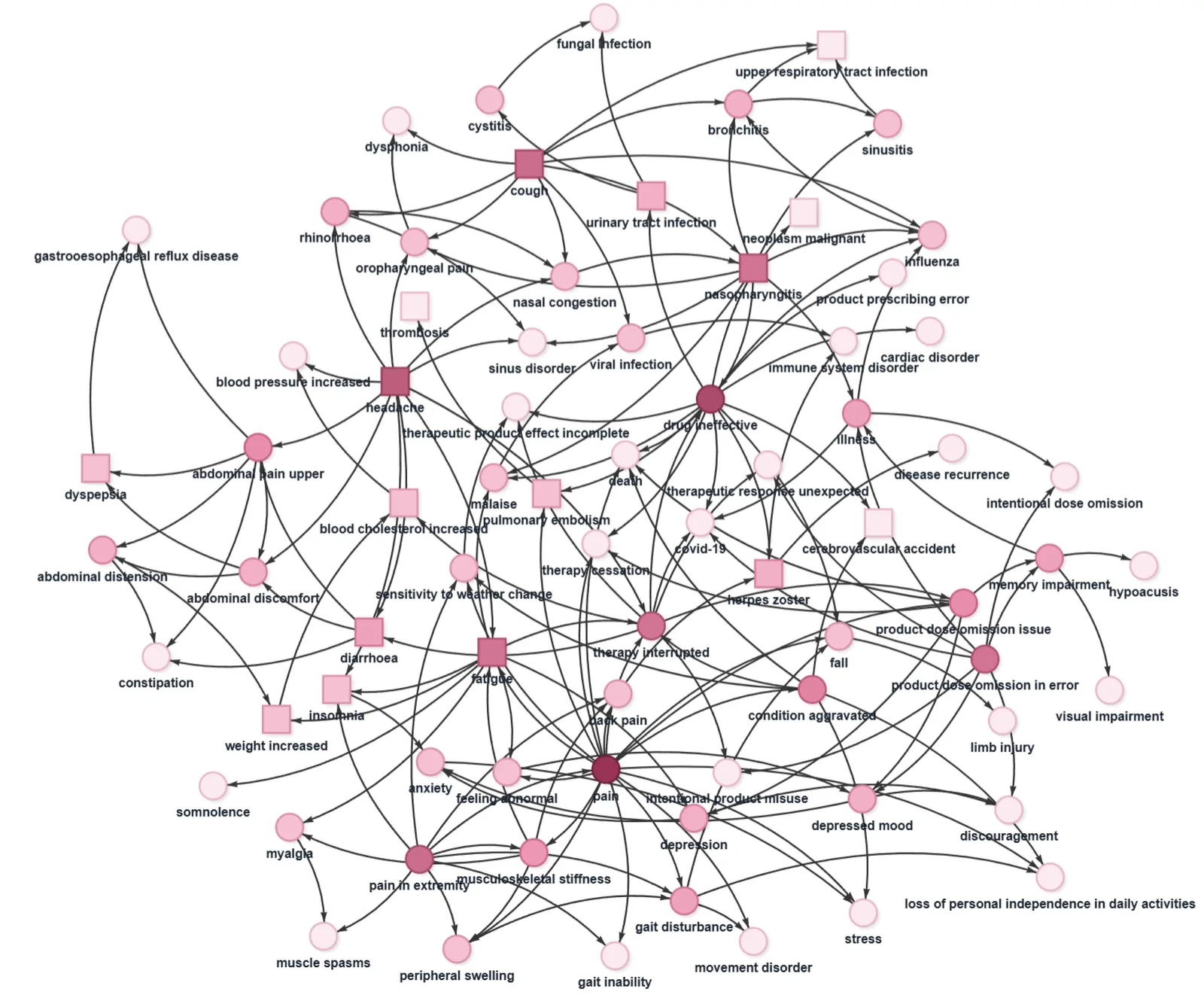
Bayesian ego network of adverse events in tofacitinib-related reports. Edges represent model-derived conditional associations supported by the bootstrap analysis. Arrowheads show the model-derived orientation of the learned structure and do not indicate temporal sequence or causal direction. Node positions were manually adjusted to reduce overlap. The complete interactive Bayesian network is provided in the Supplementary Interactive Files.

The Bayesian network revealed a structured organization of tofacitinib-related reports across symptom, functional, serious-outcome, infectious, and medication-use domains. Pain had the highest total degree, followed by drug ineffective, and both nodes showed broad connectivity across these reporting domains. Therapy interrupted had the highest betweenness centrality, followed by drug ineffective, indicating their bridging positions between otherwise distinct reporting domains. The pain–therapy interrupted and drug ineffective–therapy interrupted adjacencies had bootstrap edge-inclusion frequencies of 1.000. Fatigue connected mood-related, sleep-related, and general-symptom terms, whereas product dose omission in error linked medication-use terms with perceived treatment response and cognitive or infectious events. Together, these associations linked mood-related, cognitive, symptom, perceived treatment-response, and medication-use PTs within the broader reporting structure (Figure 9).

**Figure 10:**
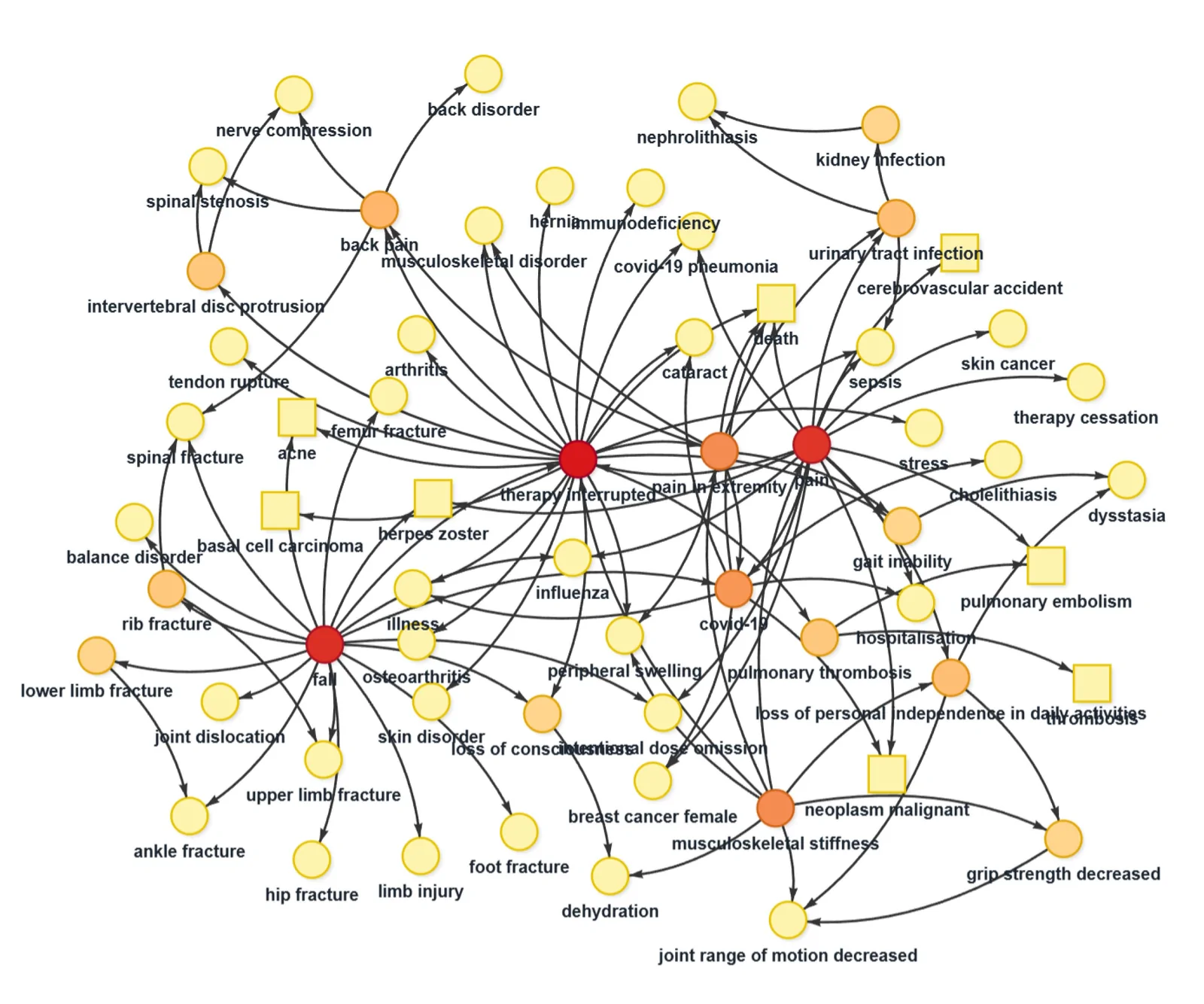
Bayesian ego network of adverse events in upadacitinib-related reports. Edges represent model-derived conditional associations supported by the bootstrap analysis. Arrowheads show the model-derived orientation of the learned structure and do not indicate temporal sequence or causal direction. Node positions were manually adjusted to reduce overlap. The complete interactive Bayesian network is provided in the Supplementary Interactive Files.

The upadacitinib Bayesian network displayed a different structure. Therapy interrupted had the highest total degree of 24 and the highest betweenness centrality (0.0201), whereas fall and pain each had a total degree of 18. Fall was connected mainly to fracture and injury-related PTs, while pain was connected with functional and mobility-related terms, including grip strength decreased, joint range of motion decreased, musculoskeletal stiffness, gait disturbance, peripheral swelling, and loss of personal independence in daily activities, as well as with therapy interrupted. The fall–therapy interrupted and pain–therapy interrupted adjacencies had bootstrap edge-inclusion frequencies of 1.000. Together, these associations defined a functional-injury reporting domain involving therapy interrupted, falls, pain, mobility-related limitation, and injury-related PTs (Figure 10; Supplementary Tables S11–S14).

### 3.6. Time-to-Onset Analysis

**Figure 11:**
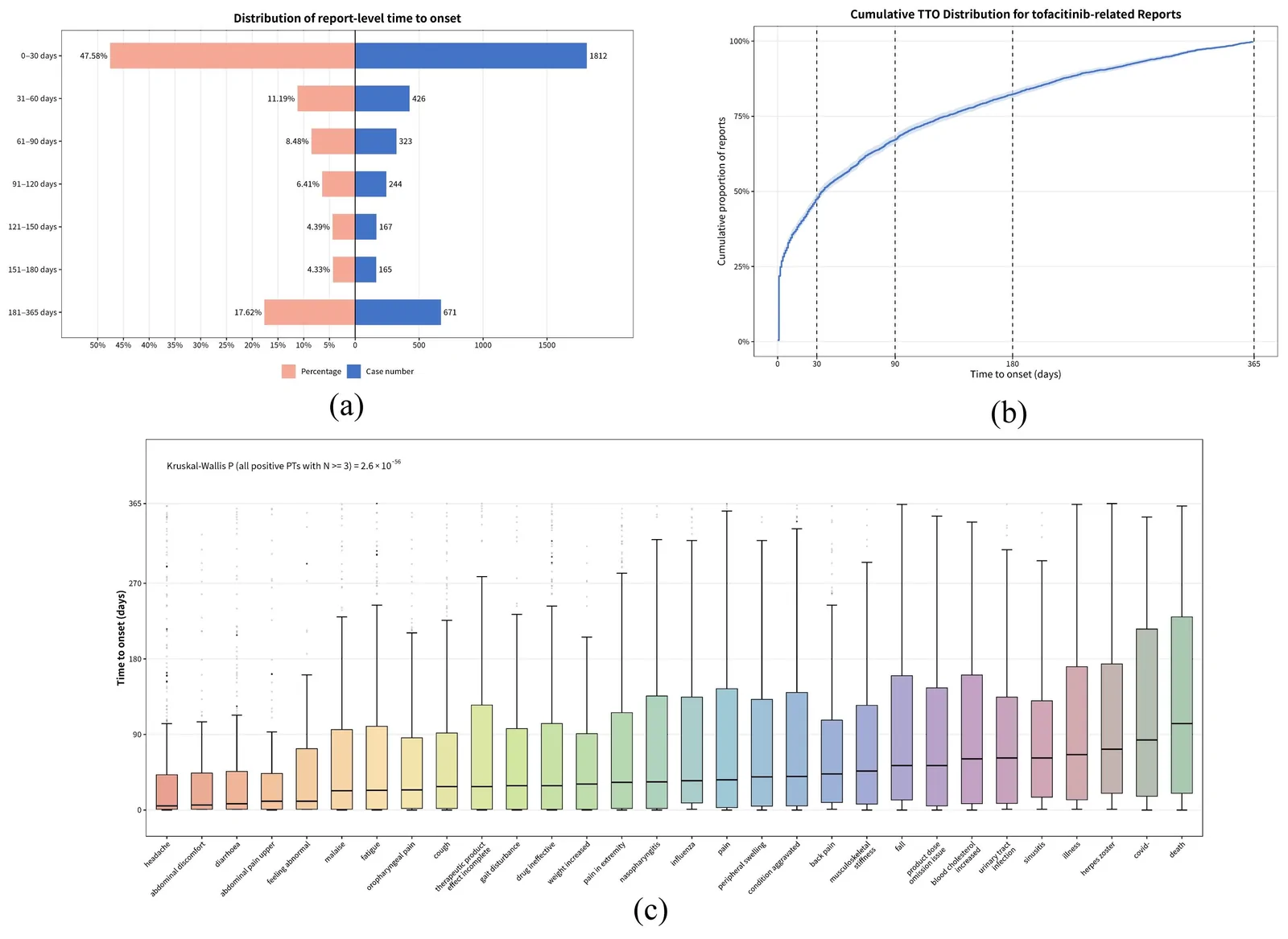
TTO analysis for eligible tofacitinib-related reports containing at least one positive PT: (**a**) report counts by interval; (**b**) cumulative TTO distribution; and (**c**) PT-specific TTO distributions. Differences in TTO distributions across positive PTs with at least three TTO observations were assessed using the Kruskal–Wallis test (*p* = 2.6 × 10^−56^).

**Figure 12:**
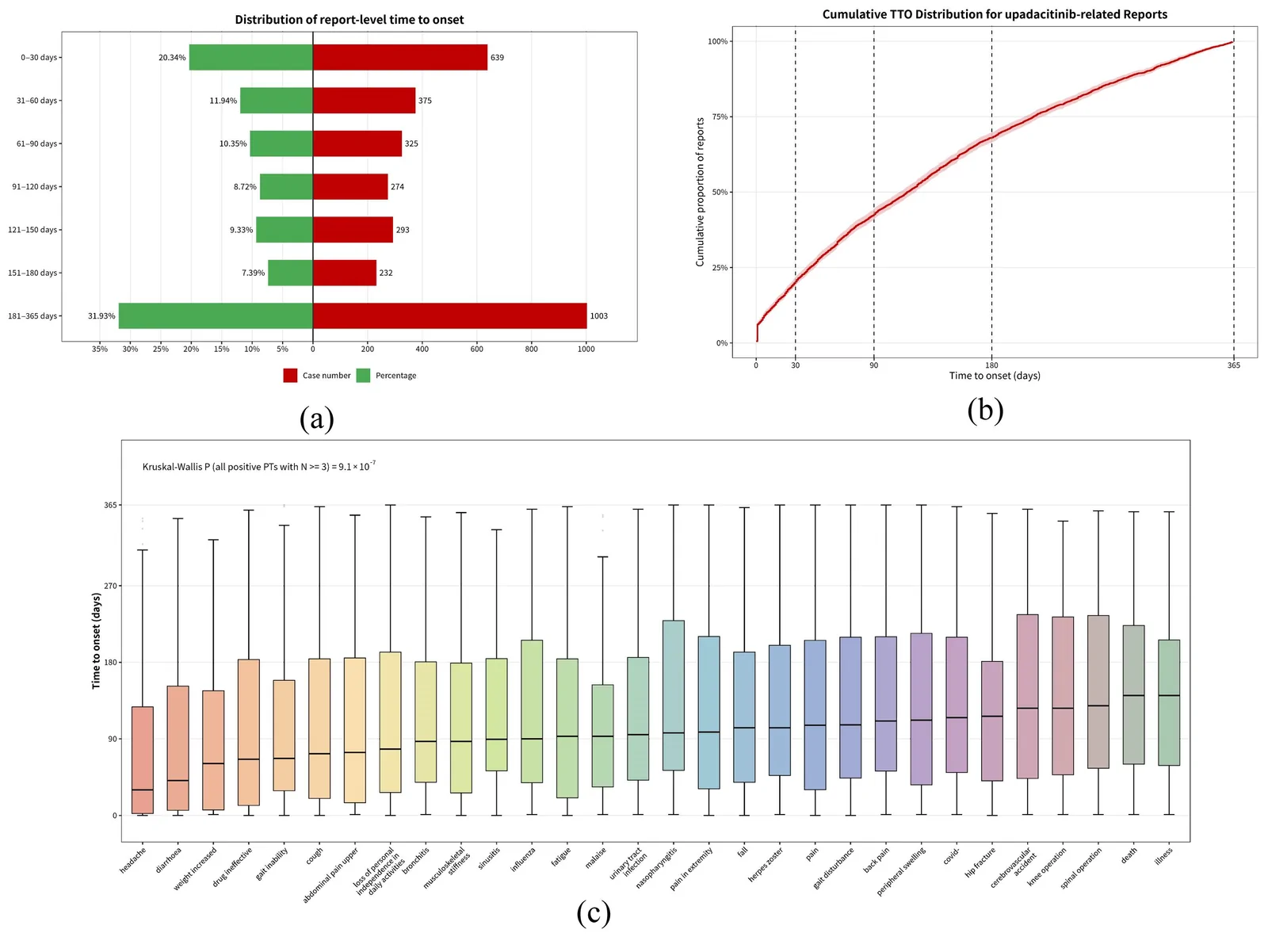
TTO analysis for eligible upadacitinib-related reports containing at least one positive PT: (**a**) report counts by interval; (**b**) cumulative TTO distribution; and (**c**) PT-specific TTO distributions. Differences in TTO distributions across positive PTs with at least three TTO observations were assessed using the Kruskal–Wallis test (*p* = 9.1 × 10^−7^).

Report-level time to onset (TTO) was evaluable for 3808 tofacitinib and 3141 upadacitinib reports containing at least one positive PT. The median TTO was 34 days (IQR, 3–129) for tofacitinib and 115 days (IQR, 41–212) for upadacitinib, with Weibull shape parameters of 0.61 (95% CI, 0.59–0.62) and 1.03 (95% CI, 1.00–1.06), respectively. According to the prespecified β-based classification rule, the fitted distribution of reported intervals for tofacitinib was characterized as early failure. In contrast, upadacitinib was classified as random failure because its 95% CI included 1, although the interval approached the classification boundary (Table 5 and Table 6; Figure 11 and Figure 12). These results characterize the distribution of intervals between the treatment-start date and the event date as recorded in the reports; they do not identify when the events biologically occurred during treatment.

Because the two drugs were approved in different years, we repeated the analysis within the common 2019–2025 calendar window. The contrast was reproduced: median TTO was 59 days (IQR, 5–174.75) for tofacitinib and 115 days (IQR, 43–213.5) for upadacitinib, with Weibull shape parameters of 0.596 (95% CI, 0.569–0.624) and 1.011 (95% CI, 0.979–1.043), preserving the early-failure versus random-failure distinction (Table S25). In the common window, 1262 of 22,687 tofacitinib positive-signal reports (5.56%) and 2715 of 17,866 upadacitinib positive-signal reports (15.20%) contributed an eligible TTO observation within 0–365 days. Within each drug, date availability was higher for health-professional than for consumer reports and was stable across the seven common reporting years; the cohort with the smaller share of health-professional reports nevertheless had the higher availability, so date completeness followed neither reporter composition nor calendar time (Table S27). Reports contributing an eligible TTO were enriched for serious reports in both drug cohorts and for health-professional reports in the tofacitinib cohort; comparisons of included and excluded reports are provided in Table S27. The contrast was also reproduced within every stratum defined by reporter type, submission type, and seriousness: median TTO was shorter for tofacitinib in all seven strata (median differences, 28–68 days; all *p* < 1 × 10^−4^, Wilcoxon rank-sum test), and the Weibull shape parameter was lower for tofacitinib with non-overlapping 95% CIs in all seven strata (tofacitinib, 0.507–0.736; upadacitinib, 0.807–1.125). Reporter type and submission type shifted the absolute values within each drug but not the ordering between drugs (Table S27). These analyses describe the interval between reported treatment start and reported event onset and do not estimate incidence or absolute onset latency.

PT-specific Weibull analyses included 222 of 316 tofacitinib-positive PTs and 164 of 413 upadacitinib-positive PTs, each with at least three valid TTO observations. PT-specific shape parameters were interpreted descriptively because the number of valid observations varied across PTs. Tofacitinib fits were more frequently classified as early-failure type, whereas upadacitinib fits were predominantly of the random-failure type; for COVID-19 and death, the estimated shape parameters and their 95% confidence intervals were entirely above 1, indicating a wear-out pattern within the truncated 0–365-day reporting window. Supplementary Table S20 reports the number of contributing reports, the proportion among all reports containing that PT, the Weibull shape parameter, and its 95% confidence interval for each PT.

## 4. Discussion

### 4.1. Differences in Adverse-Event Reporting Patterns Between the Drugs

We examined event structures beyond isolated drug–event pairs using several complementary pharmacovigilance methods. Disproportionality, network, Bayesian, and time-to-onset (TTO) analyses revealed distinct reporting patterns for the two drugs. Tofacitinib reports exhibited a symptom and treatment-experience pattern centered on fatigue, pain, mood, perceived effectiveness, and medication use. In contrast, upadacitinib reports demonstrated a functional-injury pattern involving pain, functional limitation, treatment interruption, falls, fractures, and injury. The partial overlap between FAERS and CVARD depended on signal set sizes and the availability of evaluable PTs. CVARD identified 101 positive upadacitinib PTs, limiting the maximum possible overlap to 24.5% of the 413 FAERS-positive PTs. Moreover, 270 of these 413 PTs were absent from CVARD or reported fewer than three times, precluding evaluation. The upadacitinib CVARD cohort was smaller than the tofacitinib cohort, contained fewer reactions per report (median, 2 versus 4), and consisted predominantly of study reports (Table S15). Consequently, the apparent drug-specific difference in concordance is largely attributable to the informational contribution of each CVARD cohort; thus, we interpret CVARD as a descriptive comparison rather than as external validation.

The composition of report sources provides important context. Consumers submitted most upadacitinib reports, whereas consumers and health professionals contributed similar proportions of tofacitinib reports. Patient reports can capture subjective symptoms and effects on daily functioning that are less visible in summaries focused on serious outcomes [15]. Therefore, the prominent functional signal in upadacitinib reports may be influenced by the larger proportion of consumer reports and expedited submissions in this reporting stream. Stratified networks indicate that reporter composition influences the magnitude of this contrast but not the functional domain that defines it. Falls remained the most connected node in the consumer and expedited strata, while pain, pain in extremity, falls, and femur fracture occupied the highest degree percentiles among clinician-submitted reports.

### 4.2. Co-Occurrence Networks

The fatigue-centered structure in tofacitinib reports should be interpreted as a multidimensional reporting pattern rather than as a drug-attributable effect. Fatigue may reflect the convergence of pain, sleep disturbance, functional limitation, and perceived treatment benefit [43,44]. Persistent symptoms and impaired daily functioning may be reported alongside discouragement, depressed mood, or depression; perceived treatment ineffectiveness may further increase psychological burden and influence medication-use events. Fatigue was the most connected node across all three co-occurrence networks, and the repeated depressed mood–discouragement and depression–discouragement edges defined a reproducible psychological-symptom component. In the broader PPMI network, this component extended toward myalgia, somnolence, and memory impairment, linking mood-related terms with sleep, cognitive, and general symptom domains. Overall, tofacitinib reports included fatigue, persistent symptoms, mood-related terms, and medication-use events that frequently co-occurred.

In contrast, upadacitinib-related reports were organized around a functional-injury pattern. Reduced grip strength, decreased joint range of motion, loss of personal independence in daily activities, dysstasia, pain, and peripheral swelling formed a stable functional-burden cluster across the co-occurrence networks. Falls were the central node in the Ising and phi networks and remained closely connected with hip, upper-limb, lower-limb, femur, rib, and spinal fractures, whereas pain was co-central with falls and several mobility-related terms in the PPMI network. Previous studies have reported links between rheumatoid arthritis, functional limitation, falls, and fractures [45,46,47].

The possibility that these configurations reflect rheumatoid arthritis itself was addressed at two levels. All disproportionality estimates were obtained within the rheumatoid arthritis reporting background; thus, the reference group for each drug was the remainder of the rheumatoid arthritis reports. Consequently, arthralgia, the cardinal manifestation of the disease, failed to reach the positive-signal criterion in any of the three cohorts. The methotrexate comparator, drawn from the same indication and observation period, produced networks organized around disease-activity terms. Pain and falls, the two most central nodes of the upadacitinib networks, did not reach the criterion in that cohort. Fatigue and loss of personal independence in daily activities did reach it, indicating that elevated reporting of these two terms is not specific to either study drug. What distinguishes the tofacitinib networks is the position fatigue occupies within them, at the 99th to 100th degree percentile, compared with the 94th, 66th, and 57th percentiles in the comparator networks (Table S32). Disease-related symptom burden is therefore present in the source data and is recovered when the exposure is a conventional disease-modifying agent, but it does not fully account for the fall-centered functional structure seen with upadacitinib or for the strength of the psychological associations seen with tofacitinib. The comparator remains imperfect, as methotrexate differs from the two study drugs in route of administration, position in the treatment sequence, and the calendar period during which its reports accumulated within the study window.

### 4.3. Bayesian Network Analysis

Bayesian findings suggest that the two treatments were associated with different forms of patient-experienced burden in spontaneous reports. For tofacitinib, the fatigue-centered co-occurrence pattern and the high connectivity of pain, perceived ineffectiveness, and treatment interruption are consistent with a symptom-experience structure in which persistent physical symptoms, inadequate perceived response, and treatment-related difficulties are reported together. Links between fatigue, sleep disturbance, depressive terms, discouragement, and cognitive or somatic symptoms indicate that the psychological component was embedded in a broader experience of reduced well-being rather than representing an isolated psychiatric signal. Fatigue may capture the co-reporting of disease burden, persistent symptoms, sleep disturbance, and perceived treatment dissatisfaction.

The upadacitinib network points to a different form of burden centered on physical capability and treatment continuity. The convergence of functional limitation in the co-occurrence networks with the central positions of pain, falls, and treatment interruption in the Bayesian structure suggests that mobility impairment and reduced independence were connected reporting domains. Pain, reduced strength, restricted movement, and impaired gait were frequently reported together with falls and injury-related PTs, and reports containing functional limitation also frequently included treatment interruption or discontinuation. Mood-related terms had comparatively limited centrality and were not the principal organizing feature of the upadacitinib reports.

These findings suggest that the reported burden differed between the two treatments, although the contrast may partly reflect differences in reporter composition and reporting practices. Tofacitinib reports were characterized by linked fatigue, mood, sleep, perceived effectiveness, and medication-use terms. Upadacitinib reports showed a functional-injury reporting pattern involving mobility loss, independence, treatment persistence, falls, and injury. The Bayesian analysis is most useful for identifying clinical domains with central or bridging roles within the reporting structure; its directed edges were treated as model-derived descriptors of conditional association and were not interpreted as causal or temporal relationships. This distinction is supported by the bootstrap results. Adjacencies were recovered consistently across resamples, with median edge-inclusion frequencies of 0.921 for tofacitinib and 0.909 for upadacitinib, whereas orientation was reproducible for some adjacencies and close to indeterminate for others. Orientation stability fell below 0.80 for 42.5% of tofacitinib edges and 30.0% of upadacitinib edges, and several adjacencies recovered in every resample had orientation stability near 0.50, including condition aggravated and therapy interrupted in tofacitinib (edge-inclusion frequency 1.000, orientation stability 0.542) and pain and therapy interrupted in upadacitinib (1.000 and 0.565). Hill-climbing selects one member of a Markov equivalence class, so arcs of this kind indicate that two terms remain dependent after conditioning on the other terms in the network, without indicating which term precedes or produces the other. The network is therefore presented as a structural summary of the reporting data, and the interpretation offered above rests on the adjacencies and on the conditional-independence pattern rather than on the arrowheads.

Consequently, the direction of these associations remains undetermined, substantially affecting the interpretation of the functional-injury structure. The near-indeterminate orientation of the pain–therapy interruption adjacency noted above indicates that the data cannot distinguish whether functional deterioration leads to treatment interruption or vice versa. Worsening pain, reduced grip strength, and impaired gait may prompt treatment discontinuation when patients perceive inadequate efficacy. Furthermore, reports prepared at discontinuation may retrospectively aggregate accumulated complaints, presenting functional impairments as evidence of perceived ineffectiveness. Conversely, treatment interruption may lead to the return or worsening of disease activity and instability, potentially increasing fall risk. Because FAERS does not provide reliable onset dates or event sequences within individual reports, the functional-injury structure should be regarded as hypothesis-generating.

### 4.4. Interpretation of Time-to-Onset Patterns

TTO describes the interval between the treatment-start date and the event date recorded in a report. Reports meeting the date and 0–365-day eligibility criteria represented 5.56% of tofacitinib and 15.20% of upadacitinib positive-signal reports in the common window. This subset was enriched for serious reports in both drug cohorts and for health-professional reports in the tofacitinib cohort, indicating that the analyzed reports differed from those excluded. Reporting behavior and the completeness and accuracy of recorded dates may therefore influence the observed TTO distributions. The between-drug contrast persisted in the common-window and stratified analyses, but these analyses do not eliminate selection bias related to date availability or reporting practices. Therefore, the longer recorded intervals for upadacitinib should not be interpreted as evidence that its associated adverse events actually occur later during treatment. These findings generate hypotheses for evaluation in longitudinal cohorts with prospectively recorded treatment exposure and event onset.

### 4.5. Limitations

This study has several limitations. First, spontaneous reporting systems are subject to underreporting, reporting surges following regulatory actions or heightened public attention, and variations in reporting patterns across reporters, regions, and time periods. The absence of reliable exposure denominators and treatment-duration data precludes the calculation of incidence estimates or the establishment of comparative risk. Consequently, PTs supported by few reports were interpreted as exploratory signals rather than definitive drug-related adverse events. Differences in reporter composition and submission type may influence between-drug reporting patterns, and stratified analyses cannot fully exclude these effects. Similarly, variations in database coverage, report volume, reporter composition, and reporting context imply that overlap between FAERS and CVARD should not be interpreted as formal validation or a direct measure of reproducibility. Second, confounding by indication and symptoms attributable to RA itself may have influenced the observed event patterns. Concomitant medications and potential drug–drug interactions may also have contributed to reported events, but the available data did not allow for the isolation of these effects. Third, co-occurrence networks depict statistical associations among events documented within the same reports rather than clinical or causal relationships. Similarly, Bayesian network edges represent conditional dependencies; their directions do not establish temporal ordering or causal pathways. The functional-injury structure is therefore compatible with reverse causation (protopathic bias), wherein deteriorating function or perceived ineffectiveness precedes and precipitates treatment interruption or discontinuation. Finally, as discussed in Section 4.4, the TTO analysis was based on the subset of reports with usable dates and describes reported intervals rather than absolute onset latency. For tofacitinib, the early-failure classification describes the shape of the reported-interval distribution and should not be interpreted as a declining hazard. For upadacitinib, the Weibull shape parameter was 1.03 (95% CI, 1.00–1.06); thus, the random-failure classification should be regarded as a boundary result rather than an established hazard pattern. The 0–365-day restriction truncates the right tail of the reporting-time distribution and may affect the estimated shape parameters. Therefore, the wear-out patterns observed for COVID-19 and death describe the selected first-year reporting window and should not be extrapolated to longer treatment durations.

## 5. Conclusions

By integrating disproportionality analysis, complementary co-occurrence networks, Bayesian networks, descriptive cross-database comparisons, and TTO analysis, this study identified clinically interpretable, multidimensional reporting structures specific to two JAK inhibitors in RA. Tofacitinib reports exhibited a symptom-focused reporting pattern characterized by fatigue and treatment experiences, including pain, mood changes, perceived effectiveness, and medication-use events. In contrast, upadacitinib reports demonstrated a broader functional-injury reporting pattern involving pain, restricted mobility, treatment interruption, falls, fractures, and injury. Many terms defining these structures—including pain, fatigue, functional limitation, and falls—may also reflect the clinical manifestations of RA itself. Nevertheless, these drug-specific configurations are unlikely to be attributed solely to the underlying RA disease burden for two reasons. First, all estimates were derived within the RA reporting background. Second, a methotrexate comparator cohort from the same indication produced networks organized around disease-activity terms rather than falls and pain. Although these features provide disease-specific context, they do not eliminate the overlap with RA manifestations. Consequently, the associations reported here should not be interpreted as indicating a causal relationship with either drug. The functional and symptom domains defining these structures remained consistent when networks were re-estimated within reporter and submission-type strata, indicating that the observed contrast cannot be explained solely by reporter composition. The contrast in recorded intervals, which were shorter for tofacitinib, persisted when both cohorts were restricted to the common 2019–2025 window and within reporter, submission-type, and seriousness strata. However, as this metric describes reported intervals among reports with usable dates and is subject to reporting behavior and date completeness, it should be regarded as hypothesis-generating. These converging patterns across analyses identify symptom, function, treatment persistence, and injury domains that may help prioritize clinical assessment and pharmacovigilance review, thereby generating hypotheses for testing in cohorts with recorded exposure.

## Figures and Tables

**Table 1:** Baseline characteristics of adverse-event reports for tofacitinib and upadacitinib in FAERS.

Characteristics	Tofacitinib n = 57,438	Upadacitinib n = 29,869
Sex, n (%)		
Female	46,953 (81.7)	23,394 (78.3)
Male	10,026 (17.5)	5723 (19.2)
Unknown	459 (0.8)	752 (2.5)
Age (years), n (%)		
<18	90 (0.2)	7 (0.0)
18–39	2951 (5.1)	833 (2.8)
40–49	5820 (10.1)	1699 (5.7)
50–64	24,340 (42.4)	8024 (26.9)
≥65	21,481 (37.4)	8029 (26.9)
Unknown	2756 (4.8)	11,277 (37.8)
Weight (kg), n (%)		
<50	682 (1.2)	427 (1.4)
50–100	11,753 (20.5)	5904 (19.8)
>100	2267 (3.9)	1349 (4.5)
Unknown	42,736 (74.4)	22,189 (74.3)
Reporter, n (%)		
Health professional	25,104 (43.7)	5369 (18.0)
Consumer	24,063 (41.9)	21,645 (72.5)
Unknown	8271 (14.4)	2855 (9.6)
Outcome, n (%)		
Death (DE)	2015 (3.5)	1160 (3.9)
Life-threatening (LT)	510 (0.9)	213 (0.7)
Hospitalization (HO)	8280 (14.4)	7333 (24.6)
Disability (DS)	455 (0.8)	317 (1.1)
Congenital anomaly (CA)	8 (0.0)	4 (0.0)
Required intervention (RI)	30 (0.1)	32 (0.1)
Other serious (OT)	14,839 (25.8)	11,913 (39.9)
Non-serious	31,301 (54.5)	8897 (29.8)
Continent, n (%)		
Americas	53,853 (93.8)	26,497 (88.7)
Asia	2223 (3.9)	1172 (3.9)
Europe	1071 (1.9)	1857 (6.2)
Oceania	241 (0.4)	75 (0.3)
Africa	30 (0.1)	124 (0.4)
Unknown	20 (0.0)	144 (0.5)
Submission type, n (%)		
Periodic	30,167 (52.5)	4894 (16.4)
Expedited	21,421 (37.3)	20,881 (69.9)
Direct	5850 (10.2)	4094 (13.7)

Data are presented as n (%). Percentages were calculated using the total number of reports in each drug cohort (tofacitinib, N = 57,438; upadacitinib, N = 29,869), including reports with unknown values. Percentages may not sum to 100% because of rounding.

**Table 2:** Properties of tofacitinib adverse-event co-occurrence networks.

Property	Ising	Phi (Φ)	PPMI
Nodes (N)	77	77	77
Connected nodes	67	71	73
Isolated nodes	10	6	4
Links (n)	256	369	498
Edge density	0.087	0.126	0.170
Central node (degree)	Fatigue (21)	Fatigue (30)	Fatigue (40)
Heaviest links (1–3)	Depressed mood—discouragement (4.132) Depression—discouragement (3.855) fall—limb injury (1.997)	Depressed mood—discouragement (0.330) Depression—discouragement (0.278) Pain in extremity—peripheral swelling (0.214)	Depressed mood—discouragement (4.123) Depression—discouragement (3.834) Rhinorrhoea—nasal congestion (3.461)
Clusters (N)	17	12	9
Clustering modularity	0.606	0.486	0.427
Small-worldness (ω)	0.208	0.130	0.155
Cross-network similarity			
	Ising—Phi (Φ)	Ising—PPMI	Phi (Φ)—PPMI
Jaccard index	0.676	0.514	0.741
Purity index	0.916	0.766	0.773

Edge weights are method-specific and are not directly comparable across columns. Ising values are L1-regularized logistic-regression coefficients on the log-odds scale; retained Phi values are non-negative phi coefficients ranging from 0 to 1, with negative associations set to zero; and PPMI values are positive pointwise mutual information values in bits. The “heaviest links (1–3)” row lists the three strongest edges according to each method’s own weighting scale. Network density, modularity, small-worldness, Jaccard similarity, and purity are dimensionless indices.

**Table 3:** Properties of upadacitinib adverse-event co-occurrence networks.

Property	Ising	Phi (Φ)	PPMI
Nodes (N)	65	65	65
Connected nodes	36	41	43
Isolated nodes	29	24	22
Links (n)	54	90	104
Edge density	0.026	0.043	0.050
Central node (degree)	fall (11)	fall (12)	pain (12)
Heaviest links (1–3)	Thrombosis—pulmonary thrombosis (2.650) Loss of personal independence in daily activities—grip strength decreased (2.391) Fall—hip fracture (2.302)	Loss of personal independence in Daily activities—grip strength decreased (0.308) Joint range of motion decreased—Grip strength decreased (0.288) Loss of personal independence in daily activities—joint range of motion decreased (0.239)	Joint range of motion decreased—grip strength decreased (4.292) Loss of personal independence in daily activities—grip strength decreased (4.106) Rib fracture—spinal fracture (4.076)
Clusters (N)	34	30	28
Clustering modularity	0.674	0.582	0.601
Small-worldness (ω)	0.027	−0.202	−0.232
Cross-network similarity			
	Ising—Phi (Φ)	Ising—PPMI	Phi (Φ)—PPMI
Jaccard index	0.600	0.519	0.830
Purity index	0.969	0.938	0.969

Edge weights are method-specific and are not directly comparable across columns. Ising values are L1-regularized logistic-regression coefficients on the log-odds scale; retained Phi values are non-negative phi coefficients ranging from 0 to 1, with negative associations set to zero; and PPMI values are positive pointwise mutual information values in bits. The “heaviest links (1–3)” row lists the three strongest edges according to each method’s own weighting scale. Network density, modularity, small-worldness, Jaccard similarity, and purity are dimensionless indices.

**Table 4:** The focus PTs in the methotrexate comparator cohort, evaluated against the same rheumatoid arthritis reporting background and with the same composite criterion as the primary analyses.

PT	Tofacitinib	Upadacitinib	Methotrexate
Arthralgia	0.83, no signal	0.90, no signal	0.81, no signal
Pain	1.45, node	1.16, node	0.82, no signal
Fall	1.15, node	1.77, node	0.79, no signal
Depressed mood	11.58, node	0.31, no signal	0.60, no signal
Grip strength decreased	0.37, no signal	1.61, node	1.21, below threshold
Fatigue	1.15, node	0.62, no signal	1.16, node
Loss of personal independence	1.36, node	1.40, node	1.75, node

Values are reporting odds ratios obtained within the rheumatoid arthritis reporting background, so the reference group for each drug is all other reports recording rheumatoid arthritis as the indication. “Node” indicates that the term met the composite positive-signal criterion and was retained as a co-occurrence network node; “no signal” indicates that it was evaluated and did not meet the criterion; “below threshold” indicates that it met the criterion but was reported too infrequently for network inclusion. None of these indicates absence from the reports. Within-network centrality percentiles are given in Supplementary Table S32.

**Table 5:** Overall report-level TTO and Weibull distribution analysis for tofacitinib-related reports containing at least one positive PT.

Cases (n)	Overall Description		Weibull Distribution Analysis				
	TTO (Days)		Scale		Shape		Weibull Shape Classification
	Median (IQR)	Min–Max	α	95% CI	β	95% CI	
3808	34 (3–129)	0–365	56.93	53.79–60.08	0.61	0.59–0.62	Early failure

α, Weibull scale parameter; β, Weibull shape parameter; CI, confidence interval; IQR, interquartile range. Descriptive TTO ranges retain the observed 0-day values; same-day values were replaced with 0.5 days only for Weibull fitting. Classification adheres to the β-based rule outlined in Section 2 and characterizes the shape of the fitted distribution of reported intervals.

**Table 6:** Overall report-level TTO and Weibull distribution analysis for upadacitinib-related reports containing at least one positive PT.

	Overall Description		Weibull Distribution Analysis				
Cases (n)	TTO (Days)		Scale		Shape		Weibull Shape Classification
	Median (IQR)	Min–Max	α	95% CI	β	95% CI	
3141	115 (41–212)	0–365	134.73	129.95–139.52	1.03	1.00–1.06	Random failure

α, Weibull scale parameter; β, Weibull shape parameter; CI, confidence interval; IQR, interquartile range. Descriptive TTO ranges retain the observed 0-day values; same-day values were replaced with 0.5 days only for Weibull fitting. Classification adheres to the β-based rule outlined in Section 2 and characterizes the shape of the fitted distribution of reported intervals.

## Data Availability

The cleaned FAERS dataset used for the primary analyses is available from Open Science Framework (OSF) at https://osf.io/zqu89/overview (accessed on 2 July 2026). The original FAERS quarterly data extract files are publicly available from the FDA at https://www.fda.gov/drugs/fdas-adverse-event-reporting-system-faers/fda-adverse-event-reporting-system-faers-latest-quarterly-data-files (accessed on 25 May 2026). The CVARD data extract is available from Health Canada at https://www.canada.ca/en/health-canada/services/drugs-health-products/medeffect-canada/adverse-reaction-database/canada-vigilance-online-database-data-extract.html (accessed on 25 May 2026). The DiAna resource used for drug-name standardization is available from its public repository at https://github.com/fusarolimichele/DiAna (accessed on 25 May 2026). The code used for the co-occurrence-network and Bayesian-network analyses is deposited at https://github.com/jimmy838/FAERS_network (accessed on 30 September 2026). We developed an R Shiny application that enables interactive exploration and manual layout adjustment of the co-occurrence networks. The application and its usage are described in the Supplementary Materials and are publicly available through Posit Connect Cloud at https://01a00e5b-12e3-4cd6-3574-6cada8d91575.share.connect.posit.cloud/ (accessed on 24 August 2026) and will be maintained by the authors and remain publicly accessible for the foreseeable future.

## Supplementary Materials

The following supporting information can be downloaded at: https://www.mdpi.com/article/10.3390/pharmaceutics18101318/s1, The Supplementary Material file contains Supplementary Methods, Tables S1–S34, and Figures S1–S7. Tables S18–S20 are provided as separate Excel files, comprising complete disproportionality signal estimates for tofacitinib (Table S18) and upadacitinib (Table S19), as well as PT-specific time-to-onset summaries and Weibull parameters for both drugs (Table S20).

## Abbreviations

The following abbreviations are used in this manuscript:

RA	Rheumatoid arthritis
FAERS	FDA Adverse Event Reporting System
TTO	Time to onset
CVARD	Canada Vigilance Adverse Reaction Online Database
CI	Confidence interval
DAS28	Disease Activity Score in 28 Joints
JAK	Janus kinase
FDA	U.S. Food and Drug Administration
DEMO	Demographic and Administrative Data
DRUG	Drug Information
REAC	Adverse Events
OUTC	Patient Outcomes
RPSR	Report Sources
THER	Therapy Dates
INDI	Indications
MedDRA	Medical Dictionary for Regulatory Activities
PT	Preferred Term
ROR	Reporting odds ratio
PRR	Proportional reporting ratio
BCPNN	Bayesian confidence propagation neural network
MGPS	Multi-item gamma Poisson shrinker
IC025	Lower 95% credible limit of the information component
EBGM05	Lower 90% credible limit of the empirical Bayes geometric mean
PPMI	Positive pointwise mutual information
LASSO	Least absolute shrinkage and selection operator
DAG	Directed acyclic graph
IQR	Interquartile range
OSF	Open Science Framework
