# Supplementary material for: Co-Occurrence Patterns of Symptom and Functional Burden Associated with Tofacitinib and Upadacitinib: A Multidimensional Analysis of Spontaneous Reports in Rheumatoid Arthritis
Source: Pharmaceutics. 2026 Oct 17;18(10):1318. doi: 10.3390/pharmaceutics18101318 (PMC13708423; doi:10.3390/pharmaceutics18101318)
Supplement: Supplementary file 1 [file pharmaceutics-18-01318-s001.zip › Supplementary_Material.pdf]

Contents

Table S1. Two-by-two contingency table for disproportionality analysis 2
Table S2. Formulae and positive-signal criteria for four disproportionality methods 2
Table S3. Formulae and implementation parameters for Ising, phi, and PPMI network estimation 3
Table S4. Formulae for network properties 3
Table S5. Top-degree PT nodes for tofacitinib co-occurrence networks 4
Table S6. Top-degree PT nodes for upadacitinib co-occurrence networks 4
Table S7. Strongest edges in tofacitinib co-occurrence networks 4
Table S8. Strongest edges in upadacitinib co-occurrence networks 5
Table S9. PT clusters in tofacitinib co-occurrence networks 6
Table S10. PT clusters in upadacitinib co-occurrence networks 7
Table S11. Top PT nodes in the tofacitinib Bayesian network 8
Table S12. Top PT nodes in the upadacitinib Bayesian network 9
Table S13. Tofacitinib Bayesian-network predecessor nodes and successors 10
Table S14. Upadacitinib Bayesian-network predecessor nodes and successors 10
Table S15. Baseline characteristics in the Canada Vigilance Adverse Reaction Online Database (CVARD) 18
Table S16. Common positive tofacitinib PT signals in CVARD and FAERS 19
Table S17. Common positive upadacitinib PT signals in CVARD and FAERS 22
Table S18. Complete tofacitinib disproportionality signals in FAERS and CVARD [Excel]
Table S19. Complete upadacitinib disproportionality signals in FAERS and CVARD [Excel]
Table S20. PT-specific TTO summaries and Weibull parameters for both drugs [Excel]
Table S21. Baseline characteristics stratified by drug and reporter type 25
Table S22. Common-window reporter-stratified signal summary 26
Table S23. Pairwise Jaccard overlap of common-window positive PT signals 26
Table S24. Selected PT signals across reporter strata in the common window 26
Table S25. Common-window TTO summary in positive-signal reports 27
Table S26. Positive-signal TTO exclusion reasons in the common window 27
Table S27. Valid TTO proportions by reporter, seriousness, and reporting year 28
Table S28. Comparison of Bayesian and co-occurrence network edge sets 30
Table S29. Edge-level bootstrap stability of the Bayesian networks 30
Table S30. Sensitivity of positive PT signals to the composite signal definition 31
Table S31. Report-count distribution and minimum-report-count sensitivity 32
Table S32. Methotrexate comparator cohort and density-corrected centrality 33
Table S33. Reporter- and submission-stratified co-occurrence networks 35
Table S34. Cross-drug structural comparison by reporter and submission stratum 38
Figure S1A. Tofacitinib adverse-event co-occurrence networks under a unified node layout 11
Figure S1B. Tofacitinib adverse-event co-occurrence networks using method-specific layouts and weights 12
Figure S2A. Upadacitinib adverse-event co-occurrence networks under a unified node layout 13
Figure S2B. Upadacitinib adverse-event co-occurrence networks using method-specific layouts and weights 14
Figure S3. PT degree centrality across co-occurrence networks 15
Figure S4. Degree measures in the Bayesian networks 16
Figure S5. CVARD data-processing workflow 17
Figure S6. Overlap of positive PT signals between CVARD and FAERS 23
Figure S7A. Tofacitinib CVARD disproportionality signal profile 23
Figure S7B. Upadacitinib CVARD disproportionality signal profile 24

Supplementary Material

Co-occurrence Patterns of Symptom and Functional Burden Associated with Tofacitinib and Upadacitinib: A Multidimensional Analysis of Spontaneous Reports in Rheumatoid Arthritis

This supplementary material provides detailed methods, tables, figures, and separate Excel datasets supporting the signal detection, co-occurrence network, Bayesian network, cross-database, and time-to-onset analyses described in the main text.

Supplementary Methods: Disproportionality Analysis

Table S1. Two-by-two contingency table for disproportionality analysis.

Drug category	Target adverse event	Other adverse events	Total
Tofacitinib	*a*	*b*	*a* + *b*
Other drugs (Non-tofacitinib)	*c*	*d*	*c* + *d*
Total	*a* + *c*	*b* + *d*	*a* + *b* + *c* + *d*

**a, reports with both the target drug and target AE; b, reports with the target drug but without the target AE; c, reports without the target drug but with the target AE; d, reports with neither.*

Table S2. Formulae and positive-signal criteria for four disproportionality analysis methods.

Method	Formula	Threshold
**ROR (Reporting Odds Ratio)**		
	$ROR = \frac{a/b}{c/d} = \frac{ad}{bc}$	$a \geq 3$
	$SE(\ln R\,OR) = \sqrt{\frac{1}{a} + \frac{1}{b} + \frac{1}{c} + \frac{1}{d}}$	lower limit of 95% CI > 1
	$95\%\ CI = \exp\left(\ln(ROR) \pm 1.96 \times SE(\ln R\,OR)\right)$	
**PRR (Proportional Reporting Ratio)**		
	$PRR = \frac{a/(a+b)}{c/(c+d)}$	$PRR \geq 2$
	$\chi^2 = \frac{(ad-bc)^2(a+b+c+d)}{(a+b)(c+d)(a+c)(b+d)}$	$\chi^2 \geq 4$
**BCPNN (Bayesian Confidence Propagation Neural Network)**		
	$IC = \log_2 \frac{P(D,E)}{P(D)P(E)} = \log_2 \frac{a(a+b+c+d)}{(a+b)(a+c)}$	
	$E(IC) = \log_2 \frac{(a+\gamma_{11})(N+\alpha)(N+\beta)}{(N+\gamma)(a+b+\alpha_1)(a+c+\beta_1)}$	$IC_{025} > 0$
	$IC_{025} = E(IC) - 2\sqrt{V(IC)}$	
**MGPS/EBGM (Multi-item Gamma Poisson Shrinker/Empirical Bayes Geometric Mean)**		
	$EBGM = \frac{a(a+b+c+d)}{(a+b)(a+c)}$	$EBGM_{05} > 2$
	$95\%\ CI = \exp(\ln(EBGM) \pm 1.96 \times SE)$	

**A positive signal required at least three reports, IC025 > 0, and satisfaction of at least one of the following criteria: ROR025 > 1, PRR ≥ 2 with chi-square ≥ 4, or EBGM05 > 2.*
$N = a + b + c + d\alpha\beta\gamma$ are Bayesian prior parameters; $\alpha_1\beta_1\gamma_{11}$ are derived from marginal probabilities.

Supplementary Methods: Event Co-occurrence Network Analysis

Table S3. Formulaes and implementation parameters for Ising, phi, and PPMI network estimation.

Ising	PPMI	Phi (φ)
$Ising_{i,j} = max\left(\frac{\beta_{i,j} + \beta_{j,i}}{2}, 0\right)$	$\mathrm{PPMI}_{i,j} = \max\left\{\log_2\left[\frac{(n_{11}+k)(N+kd)}{(n_{1.}+k)(n_{.1}+k)}\right], 0\right\}, k = 1$	$\varphi_{ij} = [\frac{n_{11}n_{00} - n_{10}n_{01}}{\sqrt{(n_{11}+n_{10})(n_{01}+n_{00})(n_{11}+n_{01})(n_{10}+n_{00})}}, 0]$

Note: Ising networks were estimated using IsingFit version 0.4 with nodewise L1-regularized binary logistic regression, EBIC tuning (γ = 0.25), and the AND rule; negative edge weights were removed. Phi and PPMI networks retained positive edges at a Bonferroni-adjusted family-wise significance level of 0.01. PPMI was calculated using additive smoothing with k = 1. Edge significance was assessed using 1,000 column-wise permutations. Empirical P values were calculated as the proportion of permuted PPMI values greater than or equal to the observed value, and positive edges were retained at a Bonferroni-adjusted family-wise significance level of 0.01. Communities were identified separately using fast-greedy modularity optimization in igraph version 2.3.0.

Table S4. Formulas for calculating network properties.

Network property	Formula
Network density	$D = \frac{m}{\binom{n}{2}} = \frac{2m}{n(n-1)}$
Degree centrality	$k_i = \sum_{j=1}^{n} a_{ij}$
Weighted modularity	$Q = \frac{1}{2W}\sum_{i,j}(w_{ij} - \frac{s_i s_j}{2W})\delta(c_i, c_j)$
Small-worldness	$\omega = \frac{L_r}{L} - \frac{C}{C_l}$
Edge-set Jaccard similarity	$J(E_1, E_2) = \frac{\lvert E_1 \cap E_2 \rvert}{\lvert E_1 \cup E_2 \rvert}$
Directional cluster purity	$P(A \to B) = \frac{1}{N}\sum_{a} \max_b \lvert {C_a}^A \cap {C_b}^B \rvert$
Symmetric mean purity	$P_{\mathrm{mean}} = \frac{P(A \to B) + P(B \to A)}{2}$

Note: Small-worldness was calculated as ω = Lr/L − C/Cl. L and C were derived from the largest connected component of the unweighted observed network. Lr was averaged across 30 Erdős–Rényi G(n, m) random networks, and Cl was calculated from a ring-lattice network with the same number of nodes and edges. Edge-set Jaccard similarity compared unweighted edge sets, and symmetric mean purity was the mean of the two directional community-purity values.

Supplementary Methods: Interactive Network Visualization Application

A custom interactive network visualization application was developed in R using the Shiny framework to facilitate the examination and presentation of co-reporting networks generated in this study. The application accepts precomputed Ising, phi-coefficient, and PPMI networks in RDS, JSON, or CSV formats. Users may also provide files containing known adverse drug reaction statuses, preferred-term report counts, or previously saved node and cluster-outline coordinates. These optional inputs enable the application to distinguish known adverse reactions, scale nodes by report frequency, and reproduce existing network layouts.

The application offers both unified and method-specific layouts. The unified layout constructs a joint network from the mean normalized edge weights of the three networks and calculates a shared set of node coordinates, thereby maintaining consistent positions for preferred terms across network displays. Method-specific layouts are calculated separately using the connections and edge weights of each individual network. Users can select the network layer that determines the displayed nodes, background communities, and dashed cluster outlines. Interactive functions include node searching, zooming, repositioning, and inspection of report counts and cluster membership across networks. Additionally, users can adjust community colors, opacity, outlines, node labels, label size, node scaling, background visibility, and the overall visual theme without altering the underlying network data.

The application calculates and displays descriptive network properties, including the numbers of nodes and edges, edge density, degree-based central nodes, strongest edges, modularity, and small-worldness. It also summarizes cross-network edge similarity, community agreement, and cluster membership. Network figures can be exported as interactive HTML, PNG, SVG, PDF, or TIFF files, and associated data can be exported in CSV, Excel, or Word-compatible formats. A built-in upadacitinib example dataset demonstrates the workflow. The application is publicly accessible via Posit Connect Cloud at https://01a00e5b-12e3-4cd6-3574-6cada8d91575.share.connect.posit.cloud/. share.connect.posit.cloud/. Upon opening the application, users can click “Try demo” and select the built-in tofacitinib or upadacitinib dataset to inspect complete co-occurrence network information, including nodes, edges, clusters, centrality measures, and network properties.

Supplementary Data

Table S5. Top 10 PT nodes with the highest degree centrality across the Ising, phi, and PPMI networks for tofacitinib.

Ising		Phi		PPMI	
PT	Degree	PT	Degree	PT	Degree
fatigue	21	fatigue	30	fatigue	40
pain	21	pain	28	pain	30
pain in extremity	18	insomnia	23	back pain	28
back pain	17	illness	22	illness	28
cough	17	back pain	21	insomnia	27
headache	16	condition aggravated	21	memory impairment	27
condition aggravated	15	gait disturbance	21	pain in extremity	27
illness	15	pain in extremity	21	headache	26
malaise	14	headache	20	malaise	26
depression	13	cough	18	peripheral swelling	26

Table S6. Top 10 PT nodes with the highest degree centrality across the Ising, phi, and PPMI networks for upadacitinib.

Ising		Phi		PPMI	
PT	Degree	PT	Degree	PT	Degree
fall	11	fall	12	back pain	12
joint range of motion decreased	7	dysstasia	11	fall	12
pain in extremity	7	pain in extremity	11	joint range of motion decreased	12
musculoskeletal stiffness	6	peripheral swelling	10	pain	12
peripheral swelling	6	back pain	9	pain in extremity	12
back pain	5	grip strength decreased	9	peripheral swelling	11
dysstasia	5	joint range of motion decreased	9	grip strength decreased	10
loss of personal independence in daily activities	5	loss of personal independence in daily activities	8	musculoskeletal disorder	10
intervertebral disc protrusion	4	pain	8	dysstasia	9
pain	4	musculoskeletal stiffness	7	musculoskeletal stiffness	9

Table S7. Top 10 strongest edges across the Ising, phi, and PPMI networks for tofacitinib.

Ising			Phi			PPMI		
**Node 1**	**Weight**	**Node 2**	**Node 1**	**Weight**	**Node 2**	**Node 1**	**Weight**	**Node 2**
depressed mood	4.13	discouragement	depressed mood	0.33	discouragement	depressed mood	4.12	discouragement
depression	3.86	discouragement	depression	0.28	discouragement	depression	3.83	discouragement
fall	2.00	limb injury	pain in extremity	0.21	peripheral swelling	nasal congestion	3.46	rhinorrhoea
anxiety	1.98	depression	nasal congestion	0.19	rhinorrhoea	abdominal distension	3.23	constipation
nasal congestion	1.96	rhinorrhoea	oropharyngeal pain	0.17	rhinorrhoea	oropharyngeal pain	3.13	rhinorrhoea
fatigue	1.94	somnolence	cough	0.16	nasopharyngitis	dyspepsia	3.06	gastrooesophageal reflux disease
intentional dose omission	1.89	product dose omission in error	nasal congestion	0.14	oropharyngeal pain	nasal congestion	2.97	oropharyngeal pain
cystitis	1.76	urinary tract infection	back pain	0.14	pain in extremity	anxiety	2.93	depression
pain in extremity	1.67	peripheral swelling	memory impairment	0.14	product dose omission issue	abdominal distension	2.91	dyspepsia
oropharyngeal pain	1.65	rhinorrhoea	cough	0.13	oropharyngeal pain	abdominal distension	2.89	abdominal pain upper

Table S8. Top 10 strongest edges across the Ising, phi, and PPMI networks for upadacitinib.

Ising			Phi			PPMI		
**Node 1**	**Weight**	**Node 2**	**Node 1**	**Weight**	**Node 2**	**Node 1**	**Weight**	**Node 2**
pulmonary thrombosis	2.65	thrombosis	grip strength decreased	0.31	loss of personal independence in daily activities	grip strength decreased	4.29	joint range of motion decreased
grip strength decreased	2.39	loss of personal independence in daily activities	grip strength decreased	0.29	joint range of motion decreased	grip strength decreased	4.11	loss of personal independence in daily activities
fall	2.30	hip fracture	joint range of motion decreased	0.24	loss of personal independence in daily activities	rib fracture	4.08	spinal fracture
rib fracture	2.21	spinal fracture	musculoskeletal stiffness	0.21	pain	ankle fracture	3.88	lower limb fracture
fall	2.20	upper limb fracture	fall	0.20	hip fracture	pulmonary thrombosis	3.82	thrombosis
intervertebral disc protrusion	2.07	nerve compression	pain in extremity	0.20	peripheral swelling	intervertebral disc protrusion	3.57	nerve compression
fall	1.99	lower limb fracture	grip strength decreased	0.20	musculoskeletal stiffness	joint range of motion decreased	3.48	loss of personal independence in daily activities
grip strength decreased	1.99	joint range of motion decreased	fall	0.18	upper limb fracture	foot fracture	3.43	rib fracture
kidney infection	1.90	nephrolithiasis	loss of personal independence in daily activities	0.17	pain	dysstasia	3.38	grip strength decreased
fall	1.81	femur fracture	loss of personal independence in daily activities	0.16	musculoskeletal stiffness	nerve compression	3.33	spinal stenosis

Table S9. PT clusters identified across the Ising, phi, and PPMI networks for tofacitinib.

Ising	Phi	PPMI
Cluster 1: back pain; condition aggravated; drug ineffective; fall; fatigue; gait disturbance; gait inability; limb injury; loss of personal independence in daily activities; movement disorder; muscle spasms; musculoskeletal stiffness; myalgia; pain; pain in extremity; peripheral swelling; sensitivity to weather change; somnolence; therapeutic product effect incomplete	Cluster 1: back pain; condition aggravated; drug ineffective; fall; fatigue; gait disturbance; gait inability; insomnia; limb injury; loss of personal independence in daily activities; movement disorder; muscle spasms; musculoskeletal stiffness; myalgia; pain; pain in extremity; peripheral swelling; sensitivity to weather change; somnolence; stress; therapeutic product effect incomplete	Cluster 1: anxiety; back pain; cardiac disorder; cerebrovascular accident; condition aggravated; depressed mood; depression; discouragement; drug ineffective; fall; feeling abnormal; gait disturbance; gait inability; hypoacusis; intentional product misuse; limb injury; loss of personal independence in daily activities; memory impairment; movement disorder; musculoskeletal stiffness; pain; pain in extremity; peripheral swelling; product dose omission in error; product dose omission issue; product prescribing error; sensitivity to weather change; stress; therapeutic product effect incomplete; therapeutic response unexpected; visual impairment
Cluster 2: abdominal discomfort; abdominal distension; abdominal pain upper; blood cholesterol increased; blood pressure increased; constipation; diarrhoea; dyspepsia; gastrooesophageal reflux disease; weight increased	Cluster 2: abdominal discomfort; abdominal distension; abdominal pain upper; acne; blood cholesterol increased; blood pressure increased; constipation; diarrhoea; dyspepsia; gastrooesophageal reflux disease; weight increased	Cluster 2: abdominal discomfort; abdominal distension; abdominal pain upper; acne; blood cholesterol increased; blood pressure increased; constipation; diarrhoea; dyspepsia; fatigue; gastrooesophageal reflux disease; headache; insomnia; muscle spasms; myalgia; somnolence; weight increased
Cluster 3: covid-19; hypoacusis; illness; intentional dose omission; memory impairment; product dose omission in error; product dose omission issue; therapeutic response unexpected; visual impairment	Cluster 3: anxiety; covid-19; depressed mood; depression; discouragement; feeling abnormal; hypoacusis; illness; intentional dose omission; intentional product misuse; memory impairment; product dose omission in error; product dose omission issue; product prescribing error; therapeutic response unexpected; therapy interrupted; visual impairment	
Cluster 4: bronchitis; cough; dysphonia; headache; immune system disorder; influenza; malaise; nasal congestion; nasopharyngitis; oropharyngeal pain; rhinorrhoea; sinus disorder; sinusitis; upper respiratory tract infection; viral infection	Cluster 4: bronchitis; cough; dysphonia; headache; immune system disorder; influenza; malaise; nasal congestion; nasopharyngitis; oropharyngeal pain; rhinorrhoea; sinus disorder; sinusitis; upper respiratory tract infection; viral infection	Cluster 3: bronchitis; cough; covid-19; dysphonia; illness; immune system disorder; influenza; intentional dose omission; malaise; nasal congestion; nasopharyngitis; oropharyngeal pain; rhinorrhoea; sinus disorder; sinusitis; therapy interrupted; upper respiratory tract infection; viral infection
Cluster 5: cystitis; disease recurrence; fungal infection; herpes zoster; urinary tract infection	Cluster 5: cystitis; disease recurrence; fungal infection; herpes zoster; urinary tract infection	Cluster 4: cystitis; disease recurrence; fungal infection; herpes zoster; urinary tract infection
Cluster 6: anxiety; depressed mood; depression; discouragement; feeling abnormal; insomnia; stress		
Cluster 7: pulmonary embolism; thrombosis	Cluster 6: pulmonary embolism; thrombosis	Cluster 5: pulmonary embolism; thrombosis
**Isolated nodes:** acne; cardiac disorder; cerebrovascular accident; death; intentional product misuse; neoplasm malignant; product prescribing error; therapy cessation; therapy interrupted; white blood cell count decreased	**Isolated nodes:** cardiac disorder; cerebrovascular accident; death; neoplasm malignant; therapy cessation; white blood cell count decreased	**Isolated nodes:** death; neoplasm malignant; therapy cessation; white blood cell count decreased

Table S10. PT clusters identified across the Ising, phi, and PPMI networks for upadacitinib.

Ising	Phi	PPMI
Cluster 1: ankle fracture; balance disorder; dehydration; fall; femur fracture; foot fracture; hip fracture; limb injury; loss of consciousness; lower limb fracture; rib fracture; spinal fracture; upper limb fracture	Cluster 1: ankle fracture; balance disorder; dehydration; fall; femur fracture; foot fracture; hip fracture; joint dislocation; limb injury; loss of consciousness; lower limb fracture; rib fracture; spinal fracture; upper limb fracture	Cluster 1: ankle fracture; dehydration; fall; femur fracture; foot fracture; hip fracture; joint dislocation; limb injury; loss of consciousness; lower limb fracture; rib fracture; spinal fracture; tendon rupture; upper limb fracture
Cluster 2: arthritis; dysstasia; gait inability; grip strength decreased; joint range of motion decreased; loss of personal independence in daily activities; musculoskeletal disorder; musculoskeletal stiffness; pain; pain in extremity; peripheral swelling	Cluster 2: arthritis; dysstasia; gait inability; grip strength decreased; joint range of motion decreased; loss of personal independence in daily activities; musculoskeletal disorder; musculoskeletal stiffness; pain; pain in extremity; peripheral swelling	Cluster 2: arthritis; balance disorder; dysstasia; gait inability; grip strength decreased; joint range of motion decreased; loss of personal independence in daily activities; musculoskeletal disorder; musculoskeletal stiffness; pain; pain in extremity; peripheral swelling; stress
Cluster 3: kidney infection; nephrolithiasis; sepsis; urinary tract infection	Cluster 3: kidney infection; nephrolithiasis; sepsis; urinary tract infection	Cluster 3: kidney infection; nephrolithiasis; sepsis; urinary tract infection
Cluster 4: back disorder; back pain; intervertebral disc protrusion; nerve compression; spinal stenosis	Cluster 4: back disorder; back pain; intervertebral disc protrusion; nerve compression; spinal stenosis	Cluster 4: back disorder; back pain; intervertebral disc protrusion; nerve compression; spinal stenosis
Cluster 5: pulmonary embolism; pulmonary thrombosis; thrombosis	Cluster 5: pulmonary embolism; pulmonary thrombosis; thrombosis	Cluster 5: pulmonary embolism; pulmonary thrombosis; thrombosis
	Cluster 6: illness; influenza; intentional dose omission; therapy interrupted	Cluster 6: illness; influenza; intentional dose omission; therapy interrupted
**Isolated nodes:** acne; basal cell carcinoma; breast cancer female; cardiac disorder; cataract; cerebrovascular accident; cholelithiasis; covid-19; covid-19 pneumonia; death; diverticulitis; hernia; herpes zoster; hospitalisation; illness; immunodeficiency; influenza; intentional dose omission; joint dislocation; neoplasm malignant; osteoarthritis; sars-cov-2 test positive; seizure; skin cancer; skin disorder; stress; tendon rupture; therapy cessation; therapy interrupted	**Isolated nodes:** acne; basal cell carcinoma; breast cancer female; cardiac disorder; cataract; cerebrovascular accident; cholelithiasis; covid-19; covid-19 pneumonia; death; diverticulitis; hernia; herpes zoster; hospitalisation; immunodeficiency; neoplasm malignant; osteoarthritis; sars-cov-2 test positive; seizure; skin cancer; skin disorder; stress; tendon rupture; therapy cessation	**Isolated nodes:** acne; basal cell carcinoma; breast cancer female; cardiac disorder; cataract; cerebrovascular accident; cholelithiasis; covid-19; covid-19 pneumonia; death; diverticulitis; hernia; herpes zoster; hospitalisation; immunodeficiency; neoplasm malignant; osteoarthritis; sars-cov-2 test positive; seizure; skin cancer; skin disorder; therapy cessation

Table S11. Top 25 PT nodes ranked by total degree in the tofacitinib Bayesian network.

Rank	PT	In-degree	Out-degree	Total degree	Betweenness
1	pain	1	18	19	0.0083
2	drug ineffective	2	14	16	0.0324
3	therapy interrupted	4	8	12	0.0351
4	headache	0	11	11	0.0000
5	fatigue	3	8	11	0.0128
6	nasopharyngitis	2	8	10	0.0160
7	cough	0	9	9	0.0000
8	pain in extremity	0	9	9	0.0000
9	product dose omission in error	0	8	8	0.0000
10	condition aggravated	1	6	7	0.0004
11	abdominal pain upper	2	5	7	0.0035
12	product dose omission issue	2	5	7	0.0093
13	musculoskeletal stiffness	2	4	6	0.0009
14	gait disturbance	3	3	6	0.0019
15	illness	3	3	6	0.0054
16	diarrhoea	2	3	5	0.0071
17	memory impairment	2	3	5	0.0068
18	abdominal discomfort	3	2	5	0.0019
19	bronchitis	3	2	5	0.0049
20	depressed mood	3	2	5	0.0019
21	influenza	4	1	5	0.0061
22	abdominal distension	2	2	4	0.0022
23	depression	2	2	4	0.0019
24	herpes zoster	2	2	4	0.0041
25	rhinorrhoea	2	2	4	0.0001

Table S12. Top 25 PT nodes ranked by total degree in the upadacitinib Bayesian network.

Rank	PT	In-degree	Out-degree	Total degree	Betweenness
1	therapy interrupted	3	21	24	0.0201
2	fall	0	18	18	0.0000
3	pain	1	17	18	0.0039
4	pain in extremity	2	8	10	0.0055
5	covid-19	3	7	10	0.0052
6	musculoskeletal stiffness	0	8	8	0.0000
7	back pain	2	4	6	0.0043
8	loss of personal independence in daily activities	2	3	5	0.0011
9	urinary tract infection	2	3	5	0.0028
10	death	4	0	4	0.0000
11	intervertebral disc protrusion	1	2	3	0.0010
12	pulmonary thrombosis	1	2	3	0.0017
13	rib fracture	1	2	3	0.0000
14	gait inability	2	1	3	0.0008
15	grip strength decreased	2	1	3	0.0000
16	loss of consciousness	2	1	3	0.0008
17	joint range of motion decreased	3	0	3	0.0000
18	peripheral swelling	3	0	3	0.0000
19	sepsis	3	0	3	0.0000
20	spinal fracture	3	0	3	0.0000
21	kidney infection	1	1	2	0.0000
22	lower limb fracture	1	1	2	0.0000
23	acne	2	0	2	0.0000
24	ankle fracture	2	0	2	0.0000
25	breast cancer female	2	0	2	0.0000

Table S13. Top 10 nodes ranked by out-degree and their outgoing adjacencies in the tofacitinib Bayesian network.

Node	Out-degree	Outgoing adjacencies
pain	18	back pain; condition aggravated; fall; fatigue; feeling abnormal; gait disturbance; musculoskeletal stiffness; peripheral swelling; product dose omission issue; pulmonary embolism; sensitivity to weather change; therapy interrupted; stress; death; loss of personal independence in daily activities; therapy cessation; movement disorder; gait inability
drug ineffective	14	fall; herpes zoster; influenza; malaise; pulmonary embolism; urinary tract infection; cerebrovascular accident; death; cardiac disorder; covid-19; neoplasm malignant; product prescribing error; therapeutic product effect incomplete; therapy cessation
headache	11	abdominal discomfort; abdominal pain upper; diarrhoea; fatigue; insomnia; nasal congestion; oropharyngeal pain; rhinorrhoea; therapy interrupted; blood pressure increased; sinus disorder
cough	9	bronchitis; influenza; nasal congestion; nasopharyngitis; oropharyngeal pain; rhinorrhoea; viral infection; upper respiratory tract infection; dysphonia
pain in extremity	9	back pain; gait disturbance; insomnia; musculoskeletal stiffness; pain; peripheral swelling; sensitivity to weather change; muscle spasms; gait inability
fatigue	8	depression; feeling abnormal; insomnia; malaise; myalgia; therapy interrupted; weight increased; somnolence
nasopharyngitis	8	bronchitis; drug ineffective; illness; influenza; malaise; oropharyngeal pain; sinusitis; sinus disorder
product dose omission in error	8	depressed mood; illness; memory impairment; discouragement; covid-19; intentional dose omission; intentional product misuse; therapeutic response unexpected
therapy interrupted	8	blood cholesterol increased; diarrhoea; drug ineffective; product dose omission issue; covid-19; therapeutic product effect incomplete; intentional product misuse; therapeutic response unexpected
condition aggravated	6	sensitivity to weather change; therapy interrupted; stress; cerebrovascular accident; death; loss of personal independence in daily activities

Edge orientations are model-derived and do not indicate temporal sequence or causal direction.

Table S14. Top 10 nodes ranked by out-degree and their outgoing adjacencies in the upadacitinib Bayesian network.

Node	Out-degree	Outgoing adjacencies
therapy interrupted	21	back pain; gait inability; intervertebral disc protrusion; loss of consciousness; pain in extremity; pulmonary thrombosis; cataract; death; illness; acne; peripheral swelling; covid-19 pneumonia; musculoskeletal disorder; arthritis; basal cell carcinoma; hernia; immunodeficiency; osteoarthritis; skin disorder; stress; tendon rupture
fall	18	covid-19; loss of consciousness; lower limb fracture; rib fracture; therapy interrupted; spinal fracture; acne; ankle fracture; balance disorder; femur fracture; foot fracture; herpes zoster; hip fracture; influenza; intentional dose omission; joint dislocation; limb injury; upper limb fracture
pain	17	covid-19; loss of personal independence in daily activities; therapy interrupted; urinary tract infection; breast cancer female; death; hospitalisation; neoplasm malignant; herpes zoster; influenza; intentional dose omission; cerebrovascular accident; covid-19 pneumonia; pulmonary embolism; sepsis; skin cancer; therapy cessation
musculoskeletal stiffness	8	grip strength decreased; loss of personal independence in daily activities; pain; pain in extremity; therapy interrupted; joint range of motion decreased; dehydration; peripheral swelling
pain in extremity	8	back pain; covid-19; gait inability; urinary tract infection; death; peripheral swelling; sepsis; musculoskeletal disorder
covid-19	7	breast cancer female; cataract; cholelithiasis; death; hospitalisation; illness; neoplasm malignant
back pain	4	back disorder; nerve compression; spinal fracture; spinal stenosis
loss of personal independence in daily activities	3	grip strength decreased; dysstasia; joint range of motion decreased
urinary tract infection	3	kidney infection; nephrolithiasis; sepsis
intervertebral disc protrusion	2	nerve compression; spinal stenosis

Edge orientations are model-derived and do not indicate temporal sequence or causal direction.

Tofacitinib

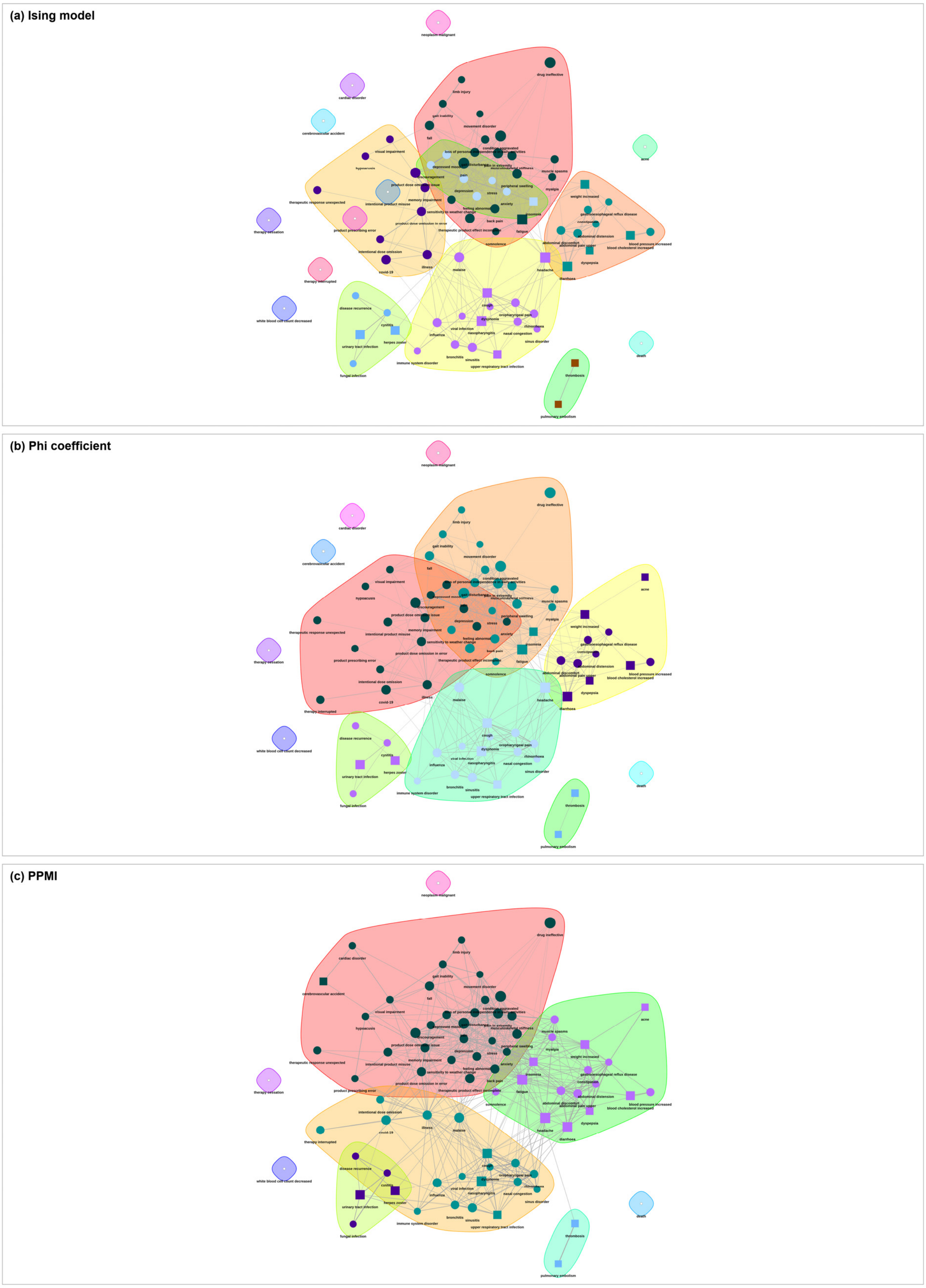

Figure S1A. Tofacitinib adverse-event co-occurrence networks under a unified node layout: (a) Ising model, (b) phi coefficient, and (c) PPMI. The same node coordinates are used across the three panels to facilitate direct structural comparison; edge width represents the normalized association weight for each method.

Tofacitinib method-specific weighted networks

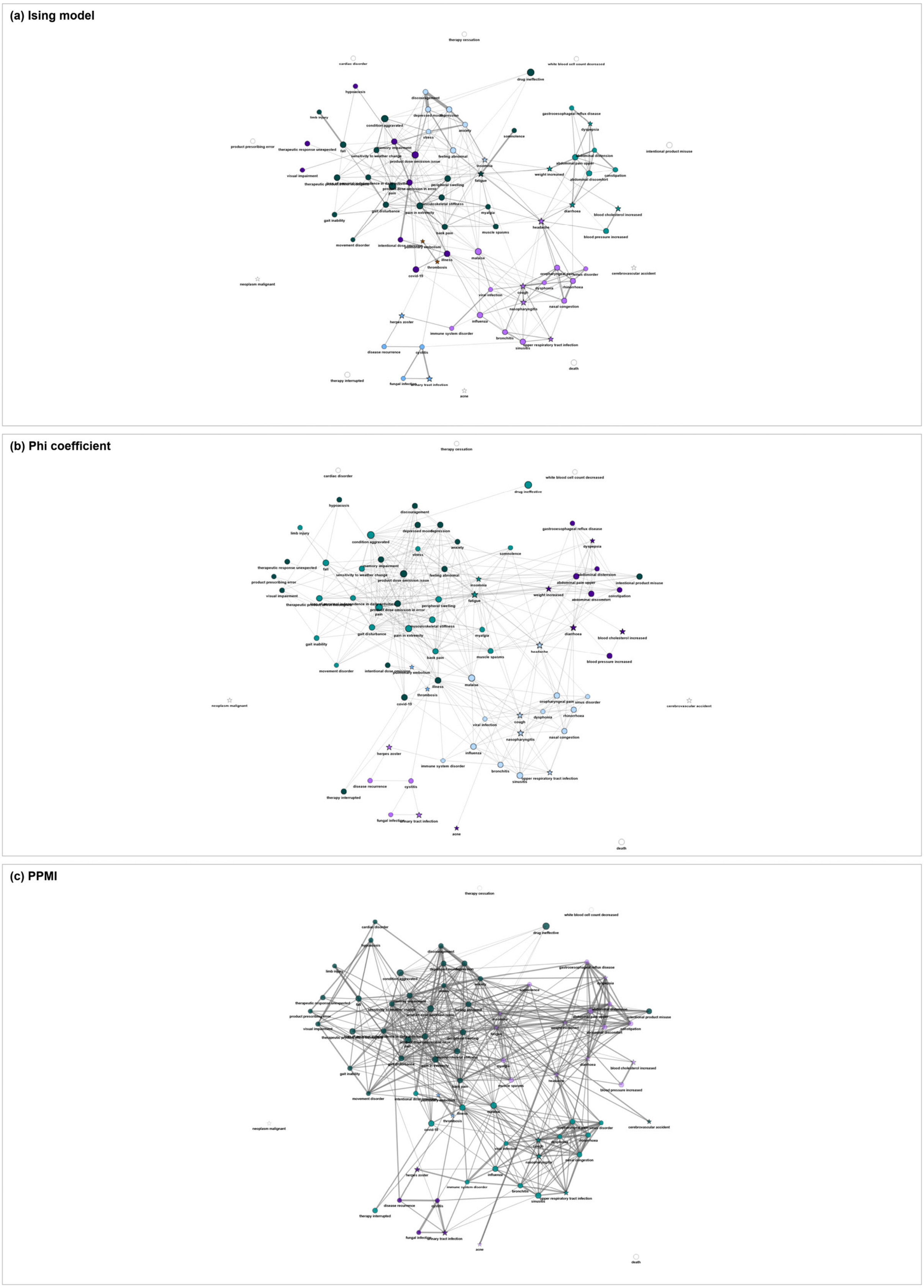

Figure S1B. Tofacitinib adverse-event co-occurrence networks using method-specific layouts and weights: (a) Ising model, (b) phi coefficient, and (c) PPMI. Node positions are optimized separately for each network, and edge width is proportional to the association weight estimated by the corresponding method.

Upadacitinib

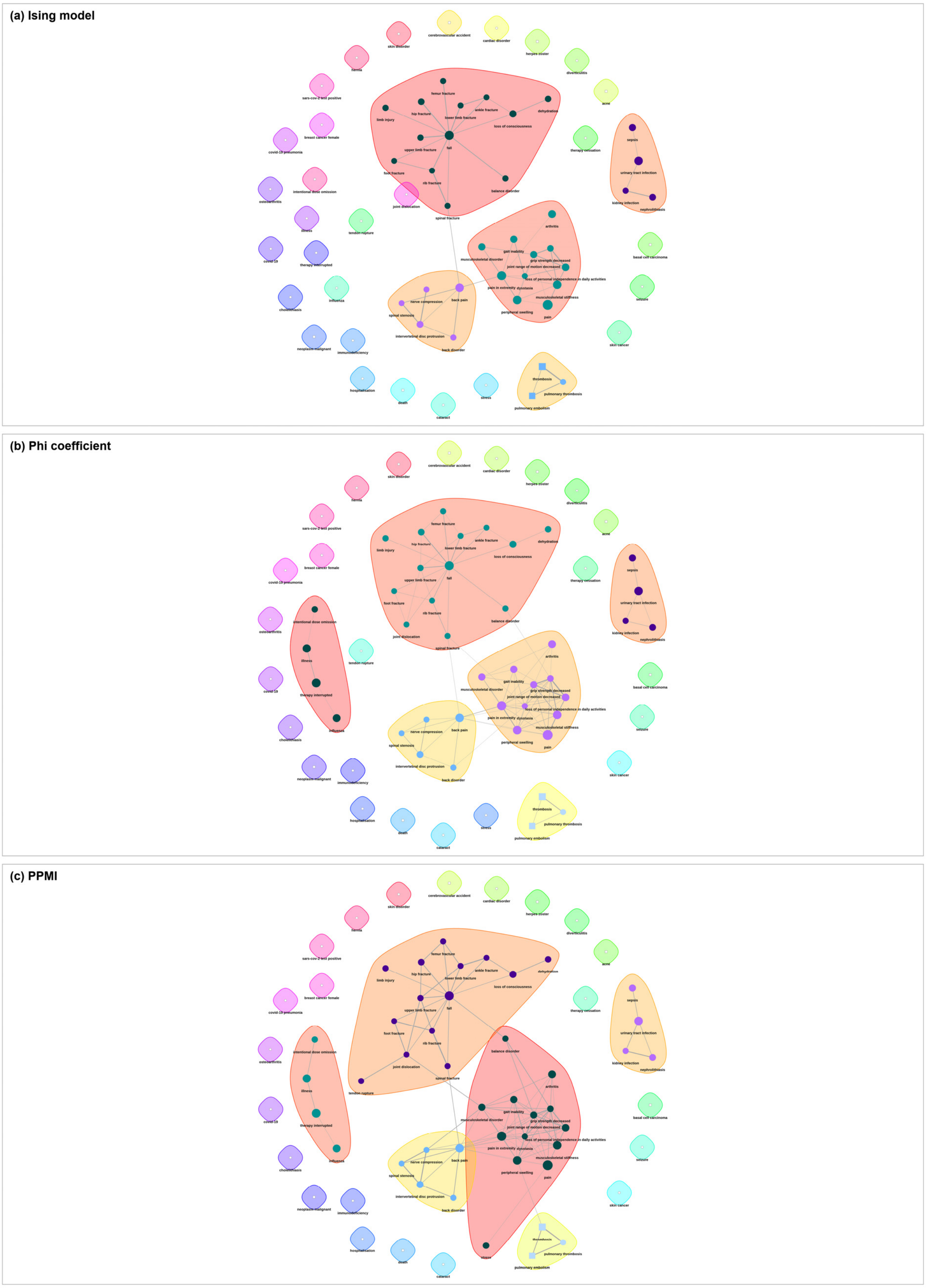

Figure S2A. Upadacitinib adverse-event co-occurrence networks under a unified node layout: (a) Ising model, (b) phi coefficient, and (c) PPMI. The same node coordinates are used across the three panels to facilitate direct structural comparison; edge width represents the normalized association weight for each method.

Upadacitinib method-specific weighted networks

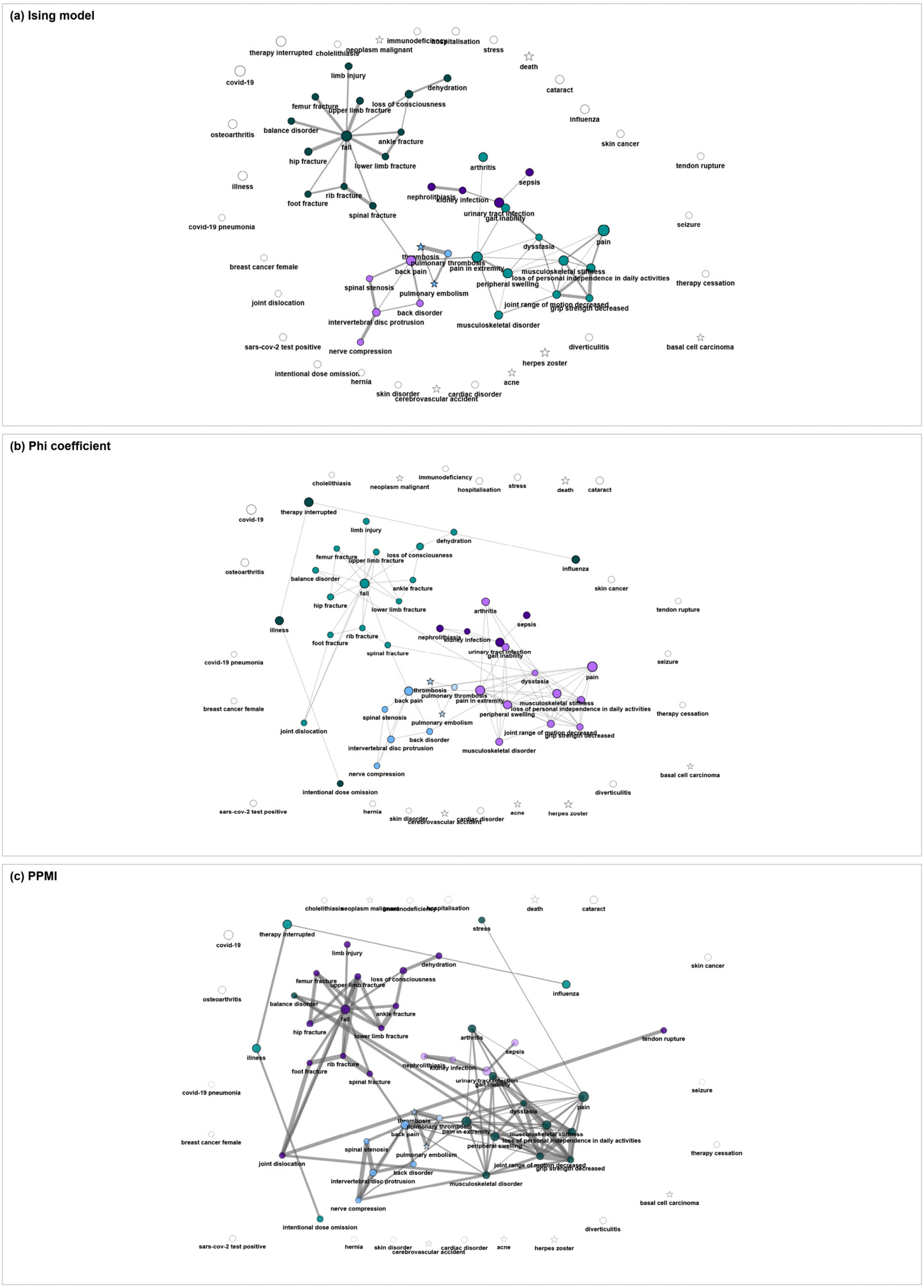

Figure S2B. Upadacitinib adverse-event co-occurrence networks using method-specific layouts and weights: (a) Ising model, (b) phi coefficient, and (c) PPMI. Node positions are optimized separately for each network, and edge width is proportional to the association weight estimated by the corresponding method.

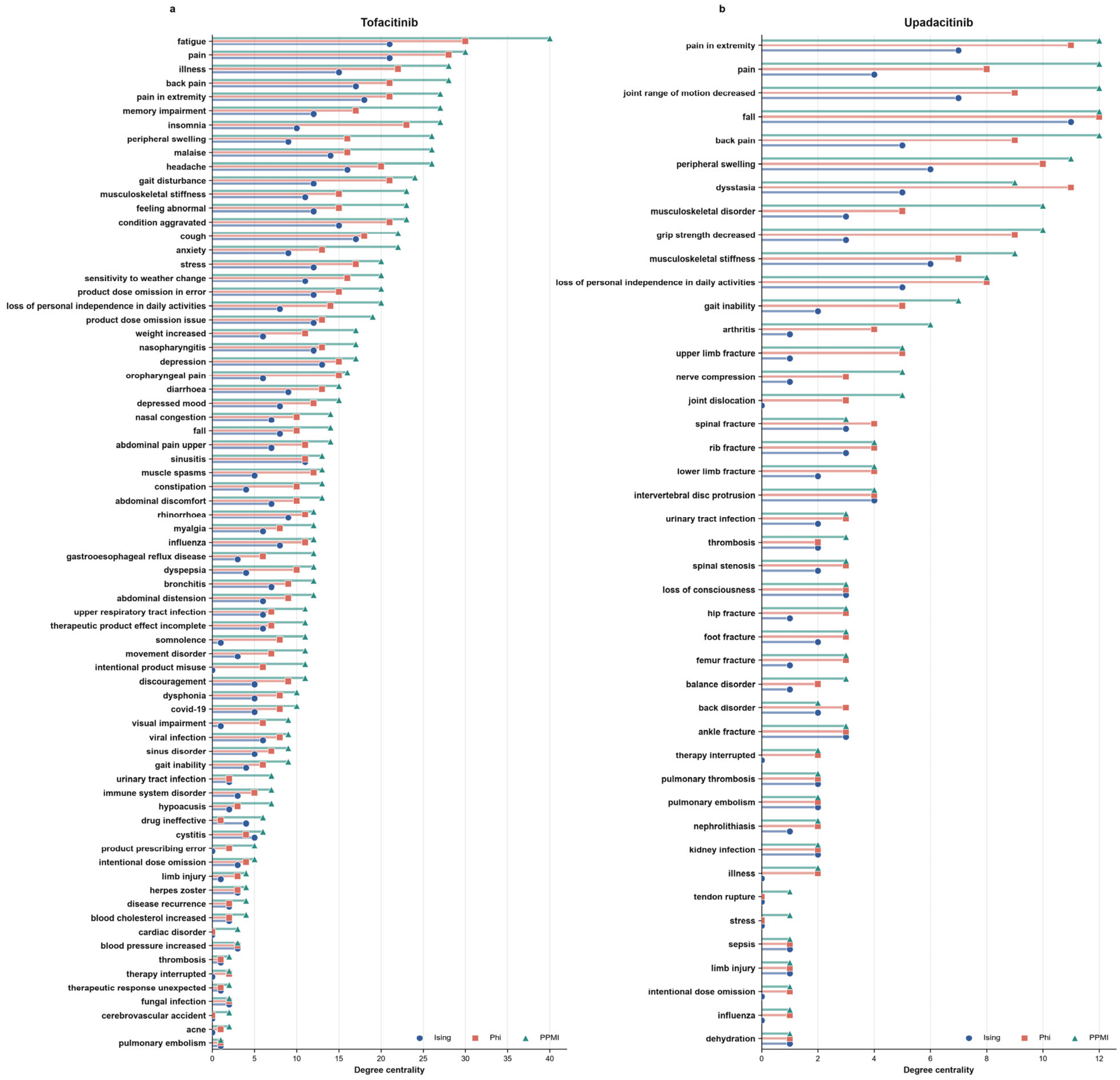

Figure S3. Degree centrality of PT nodes across the Ising, phi, and PPMI adverse-event co-occurrence networks for tofacitinib (a) and upadacitinib (b).

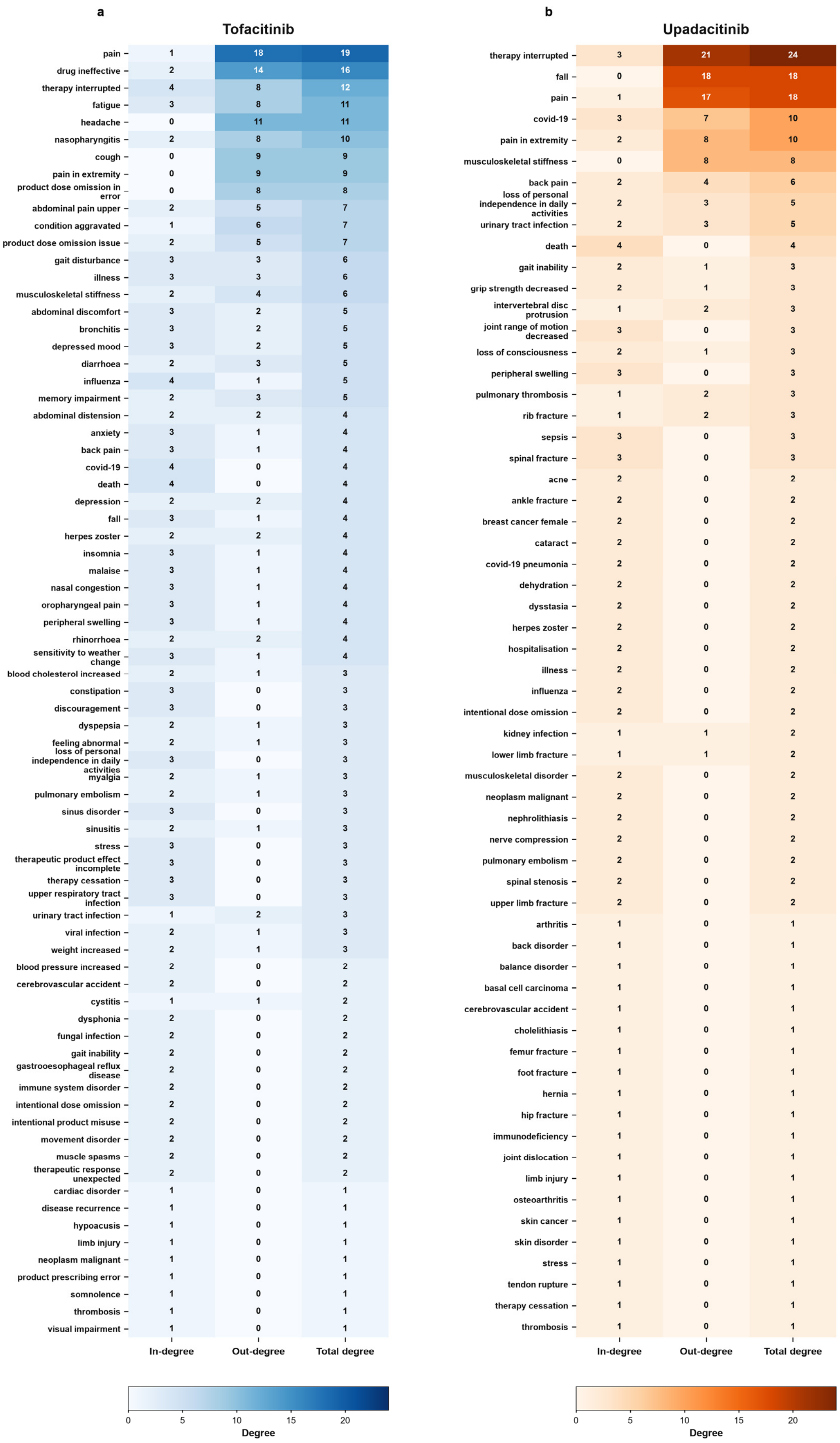

Figure S4. In-degree, out-degree, and total degree of all PT nodes in the Bayesian networks for tofacitinib (a) and upadacitinib (b).

Supplementary Analysis Using CVARD

Supplementary Methods: CVARD Data Processing

A cross-database comparison of positive preferred-term (PT) signals was conducted using the Canadian Vigilance and Risk Assessment Database (CVARD) maintained by Health Canada. Reports recorded from 1 January 2012 to 31 December 2025 were retrieved, and tofacitinib and upadacitinib were identified by generic name. The analysis was restricted to reports listing RA as the indication and the drug role as suspect. Drug names were standardized to active ingredients, and adverse events were coded at the PT level using the Medical Dictionary for Regulatory Activities (MedDRA), version 28.1. Database-specific report identifiers and update information were used to identify duplicate and follow-up reports, retaining the most recent version. The final dataset comprised 15,648 tofacitinib-related and 4,985 upadacitinib-related reports. Positive PT signals in CVARD were defined using the same composite criterion as in FAERS: at least three reports, IC025 > 0, and at least one of ROR025 > 1, PRR ≥ 2 with $\chi^2 \geq 4$, or EBGM05 > 2.

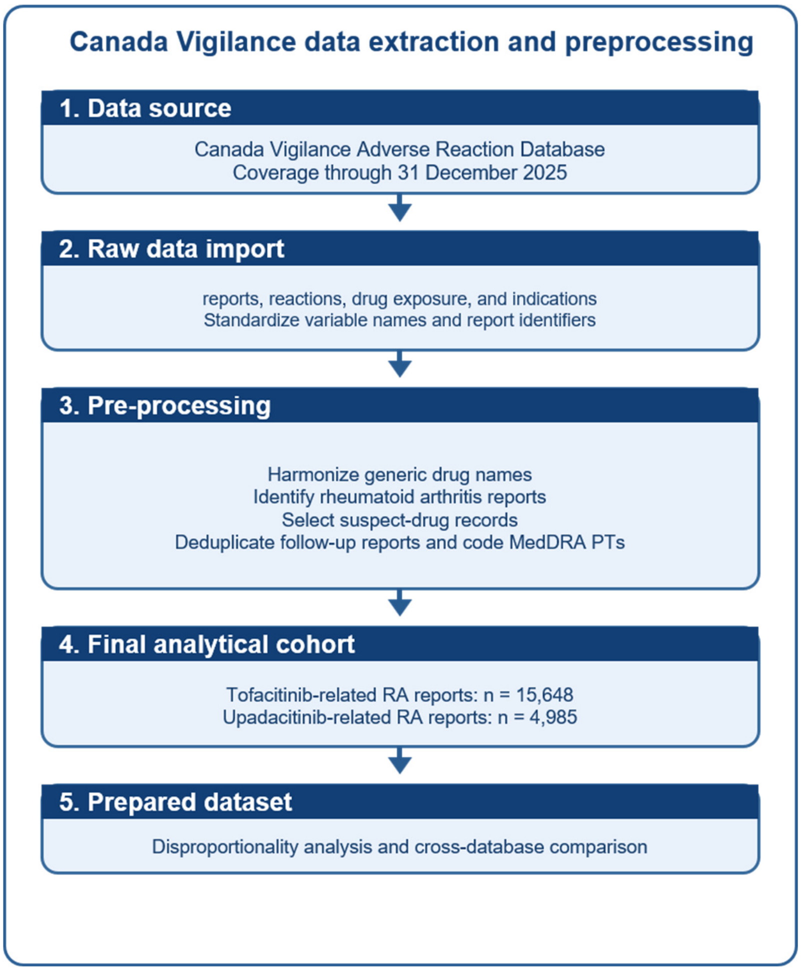

Figure S5. Data extraction and preprocessing workflow for the supplementary CVARD analysis.

CVARD Results

Baseline Characteristics of Adverse Event Reports

The CVARD database yielded 15,648 tofacitinib-related and 4,985 upadacitinib-related adverse event reports. Table S15 presents the complete descriptive characteristics of these reports.

Table S15. Baseline characteristics of tofacitinib- and upadacitinib-related reports in the CVARD.

Characteristics	Tofacitinib n = 15,648	Upadacitinib n = 4,985
**Sex, n (%)**	**Sex, n (%)**	**Sex, n (%)**
Female	12,661 (80.9%)	3,810 (76.4%)
Male	2,916 (18.6%)	1,124 (22.5%)
Unknown	71 (0.5%)	51 (1.0%)
**Age (years), n (%)**	**Age (years), n (%)**	**Age (years), n (%)**
<18	26 (0.2%)	—
18–39	1,142 (7.3%)	732 (14.7%)
40–49	2,318 (14.8%)	375 (7.5%)
50–64	6,250 (39.9%)	1,186 (23.8%)
≥65	4,821 (30.8%)	1,438 (28.8%)
Unknown	1,091 (7.0%)	1,254 (25.2%)
Median (IQR) [range]	59 (50-67) [0.1-98] (n=14,557, 93.0%)	60 (46-66) [18-97] (n=3,731, 74.8%)
**Weight (kg), n (%)**	**Weight (kg), n (%)**	**Weight (kg), n (%)**
<50	39 (0.2%)	8 (0.2%)
50–100	1,647 (10.5%)	82 (1.6%)
>100	168 (1.1%)	5 (0.1%)
Unknown	13,794 (88.2%)	4,890 (98.1%)
Median (IQR) [range]	80 (68-96) [36-225] (n=1,854, 11.8%)	70 (60.1-90) [43-200] (n=95, 1.9%)
**Seriousness, n (%)**	**Seriousness, n (%)**	**Seriousness, n (%)**
Serious	9,125 (58.3%)	1,725 (34.6%)
Not serious	6,523 (41.7%)	3,260 (65.4%)
**Outcome, n (%)**	**Outcome, n (%)**	**Outcome, n (%)**
Death (DE)	1,068 (6.8%)	88 (1.8%)
Not recovered (NR)	6,942 (44.4%)	1,194 (24.0%)
Recovered (RE)	1,214 (7.8%)	359 (7.2%)
Recovered with sequelae (RS)	8 (0.1%)	14 (0.3%)
Recovering (RG)	840 (5.4%)	245 (4.9%)
Unknown	5,576 (35.6%)	3,085 (61.9%)
**Reporter, n (%)**	**Reporter, n (%)**	**Reporter, n (%)**
Health professional	10,802 (69.7%)	435 (9.4%)
Consumer	4,451 (28.7%)	2,118 (45.9%)
Unknown	234 (1.5%)	2,066 (44.7%)
**Report source, n (%)**	**Report source, n (%)**	**Report source, n (%)**
MAH	15,417 (98.5%)	4,959 (99.5%)
Clinical Study	141 (0.9%)	—
Community	80 (0.5%)	17 (0.3%)
Hospital	9 (0.1%)	9 (0.2%)
Other	1 (0.0%)	—
**Report type, n (%)**	**Report type, n (%)**	**Report type, n (%)**
Spontaneous	14,770 (94.4%)	170 (3.4%)
Study	506 (3.2%)	4,815 (96.6%)
Other	346 (2.2%)	—

Characteristics	Tofacitinib<br>n = 15,648	Upadacitinib<br>n = 4,985
Published	16 (0.1%)	—
Unknown	10 (0.1%)	—
**Exposure and report density**	**Exposure and report density**	**Exposure and report density**
Therapy duration, median (IQR) [range]	150 (60-471) [1-7461] (n=5,140, 32.8%)	180 (79-404.1) [1-2190] (n=1,155, 23.2%)
Reaction duration, median (IQR) [range]	30 (3-31) [0-2359] (n=724, 4.6%)	12.5 (2-30) [0-730] (n=215, 4.3%)
Reactions per patient, median (IQR) [range]	4 (2-9) [1-196] (n=15,648, 100.0%)	2 (1-4) [1-159] (n=4,985, 100.0%)

MAH, marketing authorization holder; IQR, interquartile range. "—" indicates that data for the corresponding category were unavailable in the source descriptive table.

Signal Detection at the PT Level and Concordance with Overlap

CVARD identified 557 positive tofacitinib PT signals and 101 positive upadacitinib PT signals. The overlaps with the FAERS-positive signal sets comprised 129 PTs for tofacitinib and 43 PTs for upadacitinib. These figures represent 40.8% and 10.4% of the FAERS sets, respectively, but 23.2% and 42.6% of the CVARD sets. Because an overlap cannot exceed the size of the smaller signal set, the 101 positive upadacitinib PTs in CVARD established an upper bound of 24.5% on the proportion of the 413 FAERS signals that could be matched. Expressed as a fraction of this attainable maximum, the overlap was 40.8% for tofacitinib and 42.6% for upadacitinib.

We categorized the FAERS-positive PTs that were not positive in CVARD by reason. Of the 370 such upadacitinib PTs, 158 (38.3% of the 413 FAERS signals) were not reported in the CVARD cohort; 112 (27.1%) were reported fewer than three times and thus excluded from disproportionality analysis; and 100 (24.2%) were evaluated but did not meet the positive-signal criterion. The corresponding counts for tofacitinib were 24 (7.6%), 44 (13.9%), and 119 (37.7%). Only the last category represents disagreement between the two databases. When restricted to PTs that CVARD had sufficient reports to evaluate, the overlap was 129 of 248 (52.0%) for tofacitinib and 43 of 143 (30.1%) for upadacitinib. The mirror-image partition yielded a consistent picture from the CVARD perspective: of the 557 positive tofacitinib PTs, 345 (61.9%) were evaluated in FAERS without meeting the criterion, compared with 43 of 101 (42.6%) for upadacitinib.

These differences reflect the amount of information contained in each cohort. The tofacitinib CVARD cohort contributed 136,242 PT-report pairs across 15,648 reports, with a median of 4 reactions per report and 1,457 PTs reaching the three-report minimum. In contrast, the upadacitinib cohort contributed 17,744 pairs across 4,985 reports, with a median of 2 reactions per report and 679 PTs reaching that minimum. Among the seven clinically focal PTs, the two databases agreed on 9 of the 14 drug-PT comparisons, including all three negative findings for upadacitinib (arthralgia, fatigue, and depressed mood). Complete overlapping signal lists are provided in Tables S16 and S17.

Table S16. Common positive tofacitinib PT signals in CVARD and FAERS.

PT	CVARD (N)	FAERS (N)	CVARD IC (IC025)	FAERS IC (IC025)	Listing status (CVARD/FAERS)
Drug ineffective	7,713	7,360	0.51 (0.48)	0.27 (0.23)	Unlisted/Unlisted
Therapeutic product effect incomplete	2,974	2,263	1.8 (1.74)	1.35 (1.28)	Unlisted/Unlisted
Condition aggravated	2,910	6,497	1.68 (1.62)	1.78 (1.74)	Unlisted/Unlisted
Pain	2,514	6,413	0.91 (0.85)	0.45 (0.4)	Unlisted/Unlisted
Fatigue	2,426	3,452	1.17 (1.11)	0.18 (0.12)	Listed/Listed
Headache	2,017	3,687	1.29 (1.22)	0.52 (0.47)	Listed/Listed
Diarrhoea	1,372	2,343	1.23 (1.15)	0.52 (0.45)	Listed/Listed
Pain in extremity	1,363	3,245	0.87 (0.79)	0.21 (0.15)	Unlisted/Unlisted
Abdominal discomfort	1,352	1,107	1.69 (1.6)	0.12 (0.02)	Unlisted/Unlisted
Nasopharyngitis	1,282	2,412	0.94 (0.86)	0.29 (0.22)	Listed/Listed
Peripheral swelling	1,229	2,187	1.05 (0.97)	0.44 (0.36)	Unlisted/Unlisted
Musculoskeletal stiffness	1,085	1,614	1.15 (1.06)	0.23 (0.15)	Unlisted/Unlisted
Malaise	1,056	3,122	0.97 (0.88)	0.64 (0.58)	Unlisted/Unlisted
Weight increased	828	1,172	0.73 (0.63)	0.22 (0.12)	Listed/Listed
Abdominal pain upper	731	1,160	1.54 (1.43)	0.62 (0.52)	Unlisted/Unlisted
Gait disturbance	644	1,415	1.01 (0.89)	0.28 (0.19)	Unlisted/Unlisted
Sinusitis	644	1,591	0.95 (0.84)	0.09 (0.01)	Unlisted/Unlisted
Cough	628	1,878	0.55 (0.45)	0.33 (0.26)	Listed/Listed
Insomnia	621	855	1.26 (1.14)	0.18 (0.07)	Listed/Listed
Loss of personal independence in daily activities	619	828	1.3 (1.18)	0.4 (0.29)	Unlisted/Unlisted

PT	CVARD (N)	FAERS (N)	CVARD IC (IC025)	FAERS IC (IC025)	Listing status (CVARD/FAERS)
Back pain	521	1,494	0.72 (0.6)	0.28 (0.19)	Unlisted/Unlisted
Blood cholesterol increased	503	1,211	1.96 (1.81)	1.3 (1.2)	Listed/Listed
Product dose omission issue	472	3,772	1.25 (1.11)	1.27 (1.22)	Unlisted/Unlisted
Dyspepsia	438	500	1.69 (1.54)	0.48 (0.34)	Listed/Listed
Sensitivity to weather change	420	855	2.38 (2.21)	2.8 (2.68)	Unlisted/Unlisted
Influenza	407	1,465	0.62 (0.48)	0.62 (0.53)	Unlisted/Unlisted
Herpes zoster	405	1,397	1.02 (0.88)	1.02 (0.93)	Listed/Listed
Muscle spasms	396	734	1.56 (1.4)	0.44 (0.31)	Unlisted/Unlisted
Depression	320	1,127	1.26 (1.1)	0.8 (0.7)	Unlisted/Unlisted
Anxiety	303	599	1.05 (0.89)	0.16 (0.03)	Unlisted/Unlisted
Gait inability	298	557	1.38 (1.2)	0.15 (0.01)	Unlisted/Unlisted
Illness	296	1,697	0.68 (0.52)	1.65 (1.56)	Unlisted/Unlisted
Memory impairment	277	1,330	1.62 (1.43)	1.39 (1.3)	Unlisted/Unlisted
Intentional product misuse	257	1,204	1.93 (1.72)	1.99 (1.89)	Unlisted/Unlisted
Constipation	256	601	1.43 (1.24)	1.06 (0.93)	Unlisted/Unlisted
Abdominal distension	230	321	1.64 (1.43)	0.43 (0.24)	Unlisted/Unlisted
Feeling abnormal	228	1,150	1.06 (0.87)	0.84 (0.74)	Unlisted/Unlisted
Stress	222	426	0.99 (0.8)	0.38 (0.22)	Unlisted/Unlisted
Gastrooesophageal reflux disease	218	330	1.49 (1.28)	0.2 (0.02)	Unlisted/Unlisted
Myalgia	197	544	1 (0.8)	0.54 (0.4)	Unlisted/Unlisted
Rhinorrhoea	189	850	0.76 (0.57)	1.05 (0.94)	Unlisted/Unlisted
Therapeutic product effect delayed	187	145	2.33 (2.07)	1.7 (1.42)	Unlisted/Unlisted
Neoplasm malignant	167	331	0.84 (0.63)	0.48 (0.3)	Listed/Listed
Joint injury	147	285	1.2 (0.96)	0.43 (0.24)	Unlisted/Unlisted
Disease recurrence	135	342	2.03 (1.74)	2.21 (2.03)	Unlisted/Unlisted
Visual impairment	132	468	1.02 (0.77)	0.31 (0.16)	Unlisted/Unlisted
Limb discomfort	124	286	1.18 (0.92)	0.65 (0.45)	Unlisted/Unlisted
Hot flush	119	195	1.1 (0.84)	0.35 (0.11)	Unlisted/Unlisted
Acne	118	359	1.09 (0.82)	1.36 (1.19)	Listed/Listed
Therapeutic response unexpected	101	665	2.43 (2.07)	2.63 (2.5)	Unlisted/Unlisted
Thrombosis	100	421	0.77 (0.5)	0.64 (0.48)	Listed/Listed
Renal impairment	89	285	0.87 (0.57)	0.68 (0.48)	Unlisted/Unlisted
Dry eye	86	282	0.79 (0.5)	0.51 (0.31)	Unlisted/Unlisted
Poor quality sleep	86	98	1.44 (1.11)	0.73 (0.4)	Unlisted/Unlisted
Skin lesion	81	212	1.02 (0.71)	0.8 (0.57)	Unlisted/Unlisted
Flatulence	78	210	1.68 (1.32)	1.3 (1.08)	Unlisted/Unlisted
Depressed mood	75	1,039	1.52 (1.17)	2.7 (2.59)	Unlisted/Unlisted
Movement disorder	75	294	0.85 (0.53)	1.35 (1.16)	Unlisted/Unlisted
Pollakiuria	75	201	1.4 (1.06)	0.99 (0.76)	Unlisted/Unlisted
Hernia	72	150	0.96 (0.63)	0.28 (0.01)	Unlisted/Unlisted
Immune system disorder	65	298	1.36 (0.99)	1.39 (1.2)	Unlisted/Unlisted
Bone disorder	60	151	1.1 (0.73)	0.35 (0.08)	Unlisted/Unlisted
Haematochezia	59	140	0.77 (0.41)	0.31 (0.03)	Unlisted/Unlisted
Bone pain	58	222	0.91 (0.54)	0.4 (0.18)	Unlisted/Unlisted
Red blood cell count decreased	57	110	1.17 (0.79)	0.49 (0.17)	Unlisted/Unlisted
Oral pain	56	101	1.33 (0.93)	0.35 (0.02)	Unlisted/Unlisted
Product dose omission in error	55	1,598	1.76 (1.33)	3.4 (3.32)	Unlisted/Unlisted
Back disorder	54	177	0.99 (0.61)	0.53 (0.28)	Unlisted/Unlisted

PT	CVARD (N)	FAERS (N)	CVARD IC (IC025)	FAERS IC (IC025)	Listing status (CVARD/FAERS)
Deep vein thrombosis	53	201	0.89 (0.51)	0.48 (0.25)	Listed/Listed
Middle insomnia	53	71	1.56 (1.14)	1.32 (0.93)	Unlisted/Unlisted
Blood test abnormal	52	136	1.05 (0.66)	0.85 (0.57)	Unlisted/Unlisted
Hypoacusis	49	451	1.08 (0.67)	1.61 (1.45)	Unlisted/Unlisted
Skin disorder	49	228	0.77 (0.38)	0.86 (0.64)	Unlisted/Unlisted
Dysuria	48	144	0.99 (0.59)	0.49 (0.22)	Unlisted/Unlisted
Pre-existing condition improved	44	131	2.74 (2.17)	2.6 (2.31)	Unlisted/Unlisted
Drug dependence	43	73	1.79 (1.3)	0.75 (0.37)	Unlisted/Unlisted
Pelvic fracture	38	95	0.85 (0.4)	0.41 (0.07)	Unlisted/Unlisted
Brain fog	37	53	1.05 (0.59)	1.32 (0.87)	Unlisted/Unlisted
Hypokinesia	36	136	1.03 (0.56)	1.26 (0.97)	Unlisted/Unlisted
Increased appetite	36	72	1.8 (1.26)	1.14 (0.75)	Unlisted/Unlisted
Pulmonary thrombosis	35	118	0.96 (0.49)	0.61 (0.31)	Unlisted/Unlisted
Hypersomnia	32	75	0.86 (0.38)	0.44 (0.06)	Unlisted/Unlisted
Muscle strain	30	78	0.97 (0.46)	0.67 (0.29)	Unlisted/Unlisted
Emotional disorder	28	65	1.03 (0.5)	0.44 (0.03)	Unlisted/Unlisted
Decreased immune responsiveness	27	174	0.96 (0.43)	0.99 (0.74)	Unlisted/Unlisted
Chromaturia	26	67	1.44 (0.85)	0.84 (0.43)	Unlisted/Unlisted
Faeces soft	26	31	1.64 (1.03)	1.46 (0.86)	Unlisted/Unlisted
Peripheral coldness	25	71	0.96 (0.4)	0.67 (0.28)	Unlisted/Unlisted
Rosacea	25	51	1.29 (0.7)	1.19 (0.73)	Unlisted/Unlisted
Dizziness postural	24	25	1.75 (1.1)	0.69 (0.02)	Unlisted/Unlisted
Energy increased	23	47	2.52 (1.77)	1.91 (1.42)	Unlisted/Unlisted
Glomerular filtration rate decreased	23	40	1.51 (0.88)	1.27 (0.74)	Unlisted/Unlisted
Hunger	22	42	2.17 (1.45)	1.1 (0.58)	Unlisted/Unlisted
Trigger finger	21	78	1.05 (0.44)	0.53 (0.16)	Unlisted/Unlisted
Decreased activity	20	32	1.38 (0.71)	0.71 (0.12)	Unlisted/Unlisted
Micturition urgency	20	37	1.49 (0.81)	0.58 (0.04)	Unlisted/Unlisted
Parkinson's disease	20	49	1.21 (0.56)	0.61 (0.14)	Unlisted/Unlisted
Ageusia	18	58	1.4 (0.7)	0.79 (0.36)	Unlisted/Unlisted
Irritability	18	76	1.32 (0.63)	0.51 (0.13)	Unlisted/Unlisted
Eructation	15	36	1.47 (0.69)	0.59 (0.04)	Unlisted/Unlisted
Intentional underdose	15	26	2.88 (1.89)	1.95 (1.3)	Unlisted/Unlisted
Ophthalmic herpes zoster	14	43	1.13 (0.37)	1.08 (0.57)	Unlisted/Unlisted
Urine odour abnormal	14	40	1.57 (0.76)	0.96 (0.43)	Unlisted/Unlisted
Hair growth abnormal	13	65	2.21 (1.27)	2.39 (1.97)	Unlisted/Unlisted
Product dispensing error	13	102	1.4 (0.58)	1.12 (0.79)	Unlisted/Unlisted
Bowel movement irregularity	12	40	1.09 (0.28)	1.45 (0.92)	Unlisted/Unlisted
Red blood cell sedimentation rate decreased	12	16	2.05 (1.1)	1.21 (0.37)	Unlisted/Unlisted
Nocturia	10	26	1.18 (0.28)	0.84 (0.19)	Unlisted/Unlisted
Greater trochanteric pain syndrome	9	6	1.31 (0.35)	1.5 (0.08)	Unlisted/Unlisted
Initial insomnia	9	23	2.2 (1.09)	0.85 (0.15)	Unlisted/Unlisted
Root canal infection	9	15	1.69 (0.67)	1.32 (0.45)	Unlisted/Unlisted
Abnormal dreams	8	26	1.83 (0.73)	0.99 (0.34)	Unlisted/Unlisted
Dermatitis acneiform	8	18	1.9 (0.78)	1.16 (0.37)	Unlisted/Unlisted
Laziness	8	10	2.22 (1.04)	1.41 (0.33)	Unlisted/Unlisted
Low density lipoprotein increased	8	51	1.77 (0.67)	1.61 (1.15)	Listed/Listed
Lung carcinoma cell type unspecified stage IV	8	17	1.36 (0.34)	1.32 (0.51)	Unlisted/Unlisted

PT	CVARD (N)	FAERS (N)	CVARD IC (IC025)	FAERS IC (IC025)	Listing status (CVARD/FAERS)
Mental fatigue	8	12	2.05 (0.9)	1.39 (0.42)	Unlisted/Unlisted
Overweight	8	41	1.21 (0.21)	1.03 (0.51)	Unlisted/Unlisted
Mucous stools	7	14	1.49 (0.38)	0.98 (0.08)	Unlisted/Unlisted
Thinking abnormal	7	65	1.49 (0.38)	1.57 (1.16)	Unlisted/Unlisted
Seborrhoea	6	29	2.42 (1.02)	2.57 (1.95)	Unlisted/Unlisted
Accidental underdose	5	8	2.34 (0.84)	1.64 (0.42)	Unlisted/Unlisted
Metatarsalgia	5	10	2.19 (0.73)	1.23 (0.16)	Unlisted/Unlisted
Product residue present	5	61	2.72 (1.11)	2.93 (2.51)	Unlisted/Unlisted
Weight loss poor	5	17	1.82 (0.45)	1.01 (0.19)	Unlisted/Unlisted
Food interaction	4	8	2.97 (1.09)	2.49 (1.28)	Unlisted/Unlisted
Inner ear disorder	3	10	2.05 (0.26)	1.38 (0.3)	Unlisted/Unlisted
Muscle discomfort	3	8	2.05 (0.26)	2.18 (0.97)	Unlisted/Unlisted
Product solubility abnormal	3	7	2.58 (0.6)	1.85 (0.54)	Unlisted/Unlisted

All listed PTs met the combined positive-signal criterion in both databases. Rows are ordered by CVARD report count. Listing status is shown as CVARD/FAERS. IC values are presented as IC (IC025).

Table S17. Common positive upadacitinib PT signals in CVARD and FAERS.

PT	CVARD (N)	FAERS (N)	CVARD IC (IC025)	FAERS IC (IC025)	Listing status (CVARD/FAERS)
Pain	546	2,783	0.57 (0.46)	0.18 (0.12)	Unlisted/Unlisted
Pain in extremity	343	1,656	0.68 (0.54)	0.18 (0.1)	Unlisted/Unlisted
Musculoskeletal disorder	88	324	1.77 (1.47)	1.38 (1.2)	Unlisted/Unlisted
Neoplasm malignant	66	201	1.14 (0.81)	0.7 (0.47)	Listed/Listed
Acne	63	200	1.76 (1.4)	1.46 (1.22)	Listed/Listed
Cerebrovascular accident	58	305	1 (0.66)	0.44 (0.25)	Listed/Listed
Joint range of motion decreased	58	302	0.99 (0.65)	0.63 (0.44)	Unlisted/Unlisted
Skin cancer	47	204	1.48 (1.08)	1.4 (1.17)	Unlisted/Unlisted
Pulmonary embolism	45	158	1.42 (1.01)	0.36 (0.1)	Listed/Listed
Grip strength decreased	39	197	1 (0.59)	0.65 (0.42)	Unlisted/Unlisted
Back disorder	31	138	1.74 (1.23)	1.11 (0.82)	Unlisted/Unlisted
General symptom	22	27	3.53 (2.81)	0.76 (0.12)	Unlisted/Unlisted
Walking aid user	21	66	1.32 (0.73)	0.6 (0.19)	Unlisted/Unlisted
Middle insomnia	18	27	1.7 (1.04)	0.86 (0.22)	Unlisted/Unlisted
Pulmonary thrombosis	16	137	1.43 (0.76)	1.76 (1.48)	Unlisted/Unlisted
Intestinal perforation	15	40	1.66 (0.95)	0.69 (0.16)	Unlisted/Unlisted
Joint lock	15	54	1.78 (1.06)	0.88 (0.43)	Unlisted/Unlisted
Hypophagia	14	90	1.35 (0.64)	1.17 (0.82)	Unlisted/Unlisted
Inflammatory marker increased	14	78	1.38 (0.67)	1.2 (0.82)	Unlisted/Unlisted
Breast cancer female	13	109	1.61 (0.85)	1.99 (1.67)	Unlisted/Unlisted
Joint noise	13	51	1.74 (0.97)	0.91 (0.45)	Unlisted/Unlisted
Transient ischaemic attack	13	95	1.12 (0.4)	0.75 (0.41)	Unlisted/Unlisted
Urosepsis	13	42	1.78 (1.01)	0.78 (0.26)	Unlisted/Unlisted
Suspected COVID-19	11	22	2.31 (1.44)	0.95 (0.24)	Unlisted/Unlisted
Acute myocardial infarction	9	59	2.22 (1.26)	0.59 (0.16)	Unlisted/Unlisted
Papule	8	30	2.51 (1.47)	0.95 (0.34)	Unlisted/Unlisted
Troponin increased	7	13	2.8 (1.66)	1.34 (0.4)	Unlisted/Unlisted
Weight bearing difficulty	7	30	1.5 (0.5)	1.19 (0.58)	Unlisted/Unlisted
Bone cancer	5	15	1.75 (0.56)	1.2 (0.33)	Unlisted/Unlisted
Squamous cell carcinoma of skin	5	99	1.53 (0.37)	2.35 (2.02)	Unlisted/Unlisted
Haemoglobin abnormal	4	28	1.93 (0.6)	2.12 (1.49)	Unlisted/Unlisted

PT	CVARD (N)	FAERS (N)	CVARD IC (IC025)	FAERS IC (IC025)	Listing status (CVARD/FAERS)
Nephritis	4	13	3.21 (1.68)	0.95 (0.01)	Unlisted/Unlisted
Breakthrough pain	3	14	2.95 (1.27)	0.94 (0.04)	Unlisted/Unlisted
Colitis ischaemic	3	19	1.91 (0.42)	0.85 (0.08)	Unlisted/Unlisted
Coronary arterial stent insertion	3	35	1.81 (0.32)	1.76 (1.19)	Unlisted/Unlisted
Fracture pain	3	8	2.41 (0.83)	1.53 (0.32)	Unlisted/Unlisted
Gastric perforation	3	21	2.41 (0.83)	1.93 (1.2)	Unlisted/Unlisted
Low density lipoprotein increased	3	27	2.03 (0.52)	1.61 (0.97)	Listed/Listed
Obstruction	3	22	2.95 (1.27)	2.14 (1.42)	Unlisted/Unlisted
Obstruction gastric	3	7	3.25 (1.51)	1.68 (0.38)	Unlisted/Unlisted
Product lot number issue	3	8	3.9 (1.99)	1.91 (0.7)	Unlisted/Unlisted
Renal injury	3	12	2.41 (0.83)	1.2 (0.22)	Unlisted/Unlisted
Sciatic nerve neuropathy	3	12	2.1 (0.57)	1.12 (0.14)	Unlisted/Unlisted

All listed PTs met the combined positive-signal criterion in both databases. Rows are ordered by CVARD report count. Listing status is shown as CVARD/FAERS. IC values are presented as IC (IC025).

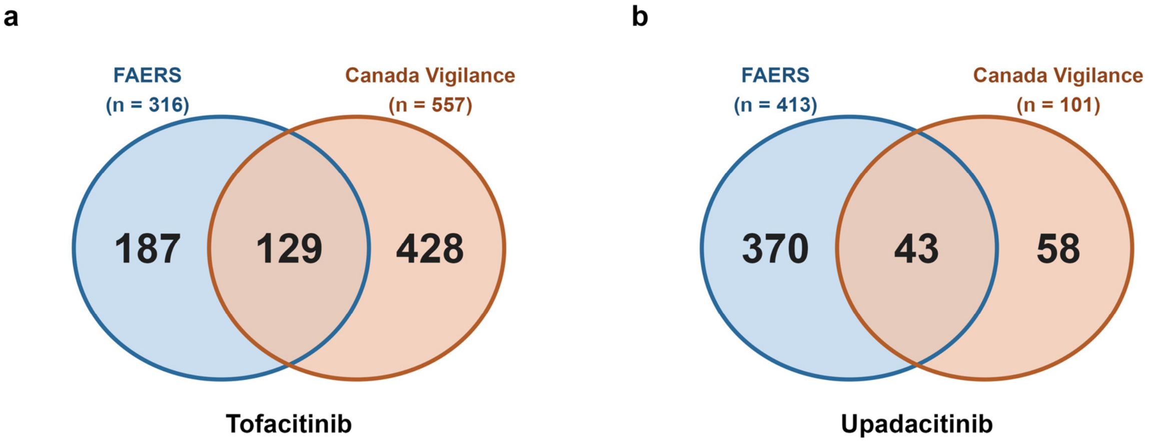

Figure S6. Overlap of positive MedDRA preferred term signals between CVARD and FAERS: (a) tofacitinib; (b) upadacitinib.

For tofacitinib, the most frequently reported positive PTs in CVARD were drug ineffective, therapeutic product effect incomplete, condition aggravated, pain, and fatigue. For upadacitinib, the leading positive PTs were drug ineffective, pain, therapeutic product effect incomplete, therapeutic product effect decreased, and pain in extremity. Figure S7 shows the regenerated forest plots.

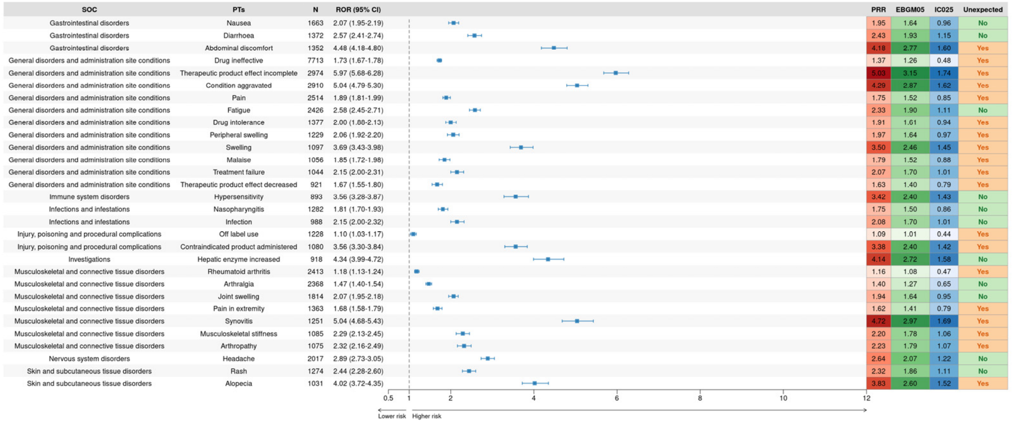

Figure S7A. Disproportionality signal profile of tofacitinib-related adverse events in CVARD at the PT level.

SOC	PTs	N	ROR (95% CI)	PRR	EBGM05	IC025	Unexpected
Gastrointestinal disorders	Intestinal perforation	15	2.95 (1.72-5.05)	2.94	1.59	0.95	Yes
General disorders and administration site conditions	Drug ineffective	2501	1.70 (1.61-1.80)	1.35	1.26	0.53	Yes
General disorders and administration site conditions	Pain	546	1.10 (1.01-1.21)	1.09	0.99	0.46	Yes
General disorders and administration site conditions	Therapeutic product effect incomplete	483	1.82 (1.65-2.01)	1.74	1.53	0.94	Yes
General disorders and administration site conditions	Therapeutic product effect decreased	426	2.43 (2.19-2.69)	2.31	1.97	1.23	Yes
General disorders and administration site conditions	Inflammation	118	1.29 (1.07-1.56)	1.29	1.05	0.55	Yes
General disorders and administration site conditions	Unevaluable event	105	9.84 (7.83-12.38)	9.66	5.68	2.59	Yes
General disorders and administration site conditions	Adverse drug reaction	68	2.29 (1.78-2.94)	2.27	1.68	1.05	Yes
General disorders and administration site conditions	Feeling abnormal	55	1.32 (1.01-1.74)	1.32	0.99	0.47	Yes
General disorders and administration site conditions	Therapeutic product effect delayed	31	2.71 (1.87-3.94)	2.70	1.74	1.07	Yes
General disorders and administration site conditions	General symptom	22	20.01 (11.33-35.32)	19.92	6.37	2.81	Yes
Infections and infestations	Tuberculosis	17	1.74 (1.06-2.84)	1.73	1.03	0.47	No
Investigations	Grip strength decreased	39	1.59 (1.15-2.20)	1.59	1.12	0.59	Yes
Metabolism and nutrition disorders	Hypophagia	14	2.20 (1.27-3.81)	2.20	1.21	0.64	Yes
Musculoskeletal and connective tissue disorders	Pain in extremity	343	1.21 (1.08-1.35)	1.20	1.06	0.54	Yes
Musculoskeletal and connective tissue disorders	Musculoskeletal disorder	88	3.31 (2.65-4.15)	3.27	2.39	1.47	Yes
Musculoskeletal and connective tissue disorders	Joint range of motion decreased	58	1.58 (1.21-2.06)	1.57	1.18	0.65	Yes
Musculoskeletal and connective tissue disorders	Back disorder	31	3.17 (2.18-4.62)	3.16	1.99	1.23	Yes
Musculoskeletal and connective tissue disorders	Myositis	17	6.18 (3.62-10.54)	6.16	2.98	1.73	Yes
Musculoskeletal and connective tissue disorders	Joint lock	15	3.31 (1.93-5.68)	3.30	1.76	1.06	Yes
Neoplasms benign, malignant and unspecified (incl cysts and polyps)	Neoplasm malignant	66	1.83 (1.42-2.35)	1.82	1.37	0.81	No
Neoplasms benign, malignant and unspecified (incl cysts and polyps)	Skin cancer	47	2.52 (1.86-3.41)	2.50	1.74	1.08	Yes
Nervous system disorders	Cerebrovascular accident	58	1.59 (1.22-2.08)	1.59	1.19	0.66	No
Psychiatric disorders	Delirium	19	1.82 (1.14-2.91)	1.82	1.10	0.55	Yes
Psychiatric disorders	Middle insomnia	18	3.06 (1.87-5.00)	3.05	1.72	1.04	Yes
Renal and urinary disorders	Pollakiuria	17	1.70 (1.04-2.78)	1.70	1.01	0.45	Yes
Respiratory, thoracic and mediastinal disorders	Pulmonary embolism	45	2.37 (1.74-3.22)	2.35	1.64	1.01	No
Respiratory, thoracic and mediastinal disorders	Pulmonary thrombosis	16	2.39 (1.43-4.00)	2.38	1.35	0.76	Yes
Skin and subcutaneous tissue disorders	Acne	63	3.26 (2.50-4.25)	3.23	2.27	1.40	No
Social circumstances	Walking aid user	21	2.15 (1.37-3.36)	2.14	1.31	0.73	Yes

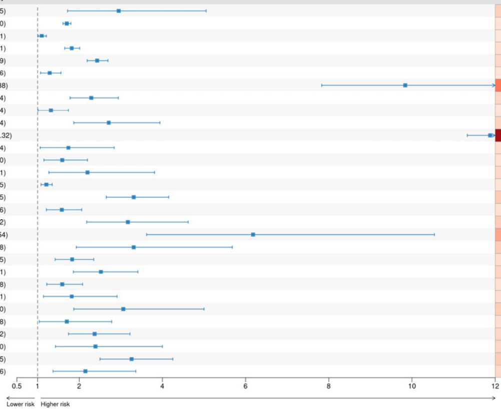

Figure S7B. Disproportionality signal profile of upadacitinib-related adverse events in CVARD at the PT level.

Supplementary Analysis Using FAERS

Two supplementary robustness analyses were conducted using FAERS. The first examined whether reporter type and submission type influenced the PT-level signal profile. The second re-estimated TTO within a common calendar window and characterized the report-level selection introduced by date requirements.

Supplementary Methods: Reporter and Submission-Type Stratification

Disproportionality analysis was repeated separately for all reports, health-professional reports, consumer reports, and expedited reports. A positive signal required at least three reports, IC025 > 0, and at least one of the following criteria: ROR025 > 1, PRR ≥ 2 with chi-square ≥ 4, or EBGM05 > 2. Reports with missing reporter or submission codes were retained as unknown in descriptive files, whereas the prespecified strata defined below were applied only when the corresponding code was available.

Reporter and Submission-Type Results

The composition of spontaneous reports differed between the two drugs. Tofacitinib exhibited a more balanced reporter mix, whereas upadacitinib had a larger consumer component and a higher proportion of expedited submissions. To address this difference directly, baseline characteristics were summarized within each drug-by-reporter stratum. Percentages in Table S21 use the corresponding stratum denominator.

Table S21. Baseline characteristics stratified by drug and reporter type in FAERS. Values are n (%); percentages use the corresponding drug-by-reporter denominator.

Dimension	Category	Tofacitinib HCP	Tofacitinib consumer	Upadacitinib HCP	Upadacitinib consumer
Report count	All reports in stratum	25,104	24,063	5,369	21,645
Sex	Female	20,176 (80.4%)	19,834 (82.4%)	3,845 (71.6%)	17,047 (78.8%)
Sex	Male	4,620 (18.4%)	4,123 (17.1%)	1,185 (22.1%)	4,200 (19.4%)
Sex	Unknown	308 (1.2%)	106 (0.4%)	339 (6.3%)	398 (1.8%)
Seriousness	Serious	12,236 (48.7%)	11,634 (48.3%)	4,023 (74.9%)	16,809 (77.7%)
Seriousness	Non-serious	12,868 (51.3%)	12,429 (51.7%)	1,346 (25.1%)	4,836 (22.3%)
Seriousness	Unknown	0 (0.0%)	0 (0.0%)	0 (0.0%)	0 (0.0%)
Submission type	Expedited	10,048 (40.0%)	9,962 (41.4%)	3,793 (70.6%)	17,085 (78.9%)
Submission type	Periodic	12,511 (49.8%)	13,872 (57.6%)	417 (7.8%)	4,473 (20.7%)
Submission type	Direct	2,545 (10.1%)	229 (1.0%)	1,159 (21.6%)	87 (0.4%)
Submission type	Unknown	0 (0.0%)	0 (0.0%)	0 (0.0%)	0 (0.0%)
Reporting year	<2012	65 (0.3%)	47 (0.2%)	1 (0.0%)	19 (0.1%)
Reporting year	2012–2018	6,630 (26.4%)	10,463 (43.5%)	28 (0.5%)	60 (0.3%)
Reporting year	2019–2021	9,573 (38.1%)	10,487 (43.6%)	2,097 (39.1%)	5,611 (25.9%)
Reporting year	2022–2025	8,836 (35.2%)	3,066 (12.7%)	3,243 (60.4%)	15,955 (73.7%)
Reporting year	Unknown	0 (0.0%)	0 (0.0%)	0 (0.0%)	0 (0.0%)

Health-professional and consumer reports are shown as the prespecified primary reporter strata. Reports with unknown reporter type were retained in the source output but are excluded from the four principal columns. The unknown-reporter groups comprised 8,271 tofacitinib reports (14.4%) and 2,855 upadacitinib reports (9.6%). Age and weight were not available as analyzable fields in the stratified export and are therefore not presented in this table.

The common 2019–2025 window was then used to align the reporter analysis with the time-to-onset (TTO) analysis. In this restricted window, the upadacitinib signal set remained closer to the consumer and expedited strata than to the health-professional stratum, whereas the tofacitinib strata showed broader overlap with one another.

Table S22. Common-window reporter-stratified signal summary for reports recorded from 2019 through 2025.

Drug	Stratum	Reports	Positive PTs	Calendar window
Tofacitinib	All reports	35,591	175	2019–2025
Tofacitinib	Health professional	18,855	156	2019–2025
Tofacitinib	Consumer	13,978	130	2019–2025
Tofacitinib	Expedited	13,453	214	2019–2025
Upadacitinib	All reports	29,869	337	2019–2025
Upadacitinib	Health professional	5,369	172	2019–2025
Upadacitinib	Consumer	21,645	245	2019–2025
Upadacitinib	Expedited	20,881	255	2019–2025

Table S23. Pairwise overlap of positive PT signals across reporter and submission strata in the common 2019–2025 window.

Drug	Stratum 1	Stratum 2	n1	n2	Intersection	Union	Jaccard
Tofacitinib	All	Health professional	175	156	109	222	0.491
Tofacitinib	All	Consumer	175	130	100	205	0.488
Tofacitinib	All	Expedited	175	214	126	263	0.479
Tofacitinib	Health professional	Consumer	156	130	59	227	0.260
Tofacitinib	Health professional	Expedited	156	214	96	274	0.350
Tofacitinib	Consumer	Expedited	130	214	91	253	0.360
Upadacitinib	All	Health professional	337	172	91	418	0.218
Upadacitinib	All	Consumer	337	245	202	380	0.532
Upadacitinib	All	Expedited	337	255	248	344	0.721
Upadacitinib	Health professional	Consumer	172	245	37	380	0.097
Upadacitinib	Health professional	Expedited	172	255	69	358	0.193
Upadacitinib	Consumer	Expedited	245	255	181	319	0.567

Selected PTs further illustrate reporter-dependent heterogeneity. These examples describe differences in signal detection across reporting strata and do not constitute evidence of causal drug-specific effects. A dash indicates that the PT did not meet the positive-signal criterion in that stratum within the common window.

Table S24. Selected PT signals across reporter strata in the common 2019–2025 window.

Drug	PT	All	Health professional	Consumer	Expedited
Tofacitinib	Pain	Positive	—	Positive	—
Tofacitinib	Drug ineffective	—	—	Positive	—
Tofacitinib	Fall	—	—	—	Positive
Tofacitinib	Fracture	Positive	—	Positive	Positive
Tofacitinib	Mood altered	Positive	—	—	—
Upadacitinib	Pain	—	—	Positive	—
Upadacitinib	Fall	Positive	—	Positive	Positive
Upadacitinib	Fracture	—	Positive	—	—
Upadacitinib	Grip strength decreased	—	—	Positive	—
Upadacitinib	Mobility decreased	—	—	Positive	—

Common-Window TTO Analysis

To reduce calendar-time differences between the drugs, TTO was re-estimated in the common 2019–2025 window. The primary TTO population was restricted to reports containing at least one positive PT signal. The common window was applied only to define the calendar window of eligible reports. Valid TTO required an event date and a therapy start date within the same report; no submission or receipt date entered any TTO calculation. TTO values below zero or above 365 days were excluded.

Table S25. Common-window TTO summary for positive-signal reports recorded from 2019 through 2025.

Drug	Positive-signal reports	Valid TTO n (%)	Median days	IQR	Weibull beta (95% CI)	Weibull shape classification
Tofacitinib	22,687	1,262 (5.56%)	59	5–174.75	0.596 (0.569–0.624)	Early failure
Upadacitinib	17,866	2,715 (15.20%)	115	43–213.5	1.011 (0.979–1.043)	Random failure

Restricting both drugs to the same calendar window reproduced the difference in median TTO and preserved the early-failure versus random-failure Weibull classification, indicating that the contrast is not produced by the different accrual periods of the drugs.

TTO Exclusion Reasons and Selection Completeness

Among positive-signal reports, 1,079 tofacitinib reports (4.76%) and 1,873 upadacitinib reports (10.48%) had TTO values above 365 days and were excluded. Missing or invalid dates and the absence of a therapy record accounted for most additional exclusions.

Table S26. TTO inclusion and exclusion reasons among positive-signal reports in the common 2019–2025 window.

Drug	Reason	N	Total positive-signal reports	%
Tofacitinib	Included	1,262	22,687	5.56
Tofacitinib	TTO >365 days	1,079	22,687	4.76
Tofacitinib	Negative TTO	1,129	22,687	4.98
Tofacitinib	Invalid START_DT/EVENT_DT	6,394	22,687	28.18
Tofacitinib	Missing START_DT/EVENT_DT	8,403	22,687	37.04
Tofacitinib	No therapy record	4,420	22,687	19.48
Upadacitinib	Included	2,715	17,866	15.20
Upadacitinib	TTO >365 days	1,873	17,866	10.48
Upadacitinib	Negative TTO	1,477	17,866	8.27
Upadacitinib	Invalid START_DT/EVENT_DT	3,246	17,866	18.17
Upadacitinib	Missing START_DT/EVENT_DT	4,092	17,866	22.90
Upadacitinib	No therapy record	4,463	17,866	24.98

Date availability differed between drugs but followed neither reporter composition nor calendar time. Within each drug, valid-TTO proportions were higher for health-professional reports than for consumer reports; yet upadacitinib, the cohort with the smaller health-professional share, had the higher overall availability. Valid-TTO proportions were also stable across the seven common reporting years within each drug, with no convergence between drugs. Serious reports were over-represented among reports contributing a valid TTO for both drugs.

Table S27. Date availability and composition of reports contributing a valid TTO among positive-signal reports in the common 2019–2025 window.

Panel A. Valid-TTO proportion

Dimension	Category	Tofacitinib valid TTO n/N (%)	Upadacitinib valid TTO n/N (%)
Overall	All positive-signal reports	1,262/22,687 (5.56)	2,715/17,866 (15.20)
Reporter	Health professional	880/12,174 (7.23)	659/2,637 (24.99)
Reporter	Consumer	337/9,323 (3.62)	2,054/13,573 (15.13)
Reporter	Unknown	45/1,190 (3.78)	2/1,656 (0.12)
Seriousness	Serious	705/9,655 (7.30)	2,495/13,940 (17.90)
Seriousness	Non-serious or not reported	557/13,032 (4.27)	220/3,926 (5.60)
Reporting year	2019	286/3,342 (8.56)	22/158 (13.92)
Reporting year	2020	276/6,391 (4.32)	190/1,675 (11.34)
Reporting year	2021	305/4,510 (6.76)	529/3,325 (15.91)
Reporting year	2022	166/3,734 (4.45)	752/4,752 (15.83)
Reporting year	2023	121/2,159 (5.61)	500/3,154 (15.85)
Reporting year	2024	61/1,425 (4.28)	405/2,645 (15.31)
Reporting year	2025	47/1,126 (4.17)	317/2,157 (14.70)

Panel A gives the proportion of positive-signal reports with a valid treatment-start and event-date pair.

Panel B. Composition of included versus excluded reports

Dimension	Category	Tofa included (n = 1,262) %	Tofa excluded (n = 21,425) %	Upa included (n=2,715) %	Upa excluded (n = 15,151) %
Reporter	Health professional	69.7	52.7	24.3	13.1
Reporter	Consumer	26.7	41.9	75.7	76.0
Reporter	Unknown	3.6	5.3	0.1	10.9
Seriousness	Serious	55.9	41.8	91.9	75.5
Seriousness	Non-serious or not reported	44.1	58.2	8.1	24.5
Reporting year	2019	22.7	14.3	0.8	0.9
Reporting year	2020	21.9	28.5	7.0	9.8
Reporting year	2021	24.2	19.6	19.5	18.5
Reporting year	2022	13.2	16.7	27.7	26.4
Reporting year	2023	9.6	9.5	18.4	17.5
Reporting year	2024	4.8	6.4	14.9	14.8
Reporting year	2025	3.7	5.0	11.7	12.1

Panel B gives the composition of reports contributing a valid TTO (included) and of those that did not (excluded); columns sum to 100% within each dimension. "Non-serious or not reported" denotes reports with no serious outcome code in the FAERS OUTC file.

Panel C. Time-to-onset by reporter type, submission type, and seriousness (common 2019–2025 window, positive-signal reports)

Stratum	Tofacitinib			Upadacitinib			Median difference (days)
	n	Median (IQR)	β (95% CI)	n	Median (IQR)	β (95% CI)	
All reports	1,262	59.00 (5.00–174.75)	0.596 (0.569–0.624)	2,715	115.00 (43.00–213.50)	1.011 (0.979–1.043)	56
Health professional	880	61.00 (6.00–173.00)	0.612 (0.578–0.646)	659	107.00 (46.00–201.00)	1.125 (1.054–1.197)	46
Consumer	337	49.00 (1.00–179.00)	0.546 (0.497–0.595)	2,054	117.00 (40.25–218.00)	0.978 (0.942–1.014)	68
Expedited	560	85.00 (14.75–200.25)	0.679 (0.631–0.728)	2,492	119.00 (45.75–216.00)	1.037 (1.002–1.071)	34
Periodic	624	32.00 (1.00–126.25)	0.533 (0.499–0.567)	183	69.00 (21.00–144.50)	0.807 (0.710–0.904)	37
Serious	705	91.00 (21.00–205.00)	0.736 (0.689–0.783)	2,495	119.00 (45.00–216.50)	1.035 (1.000–1.069)	28
Non-serious or not reported	557	24.00 (0.00–111.00)	0.507 (0.473–0.541)	220	70.00 (21.00–148.25)	0.830 (0.740–0.921)	46

n denotes reports contributing a valid TTO. β is the Weibull shape parameter. Median differences are upadacitinib minus tofacitinib; P values are from the Wilcoxon rank-sum test comparing the two drugs within the same stratum. Strata with unknown reporter type or unknown submission type contributed fewer than 80 evaluable reports per drug and are not tabulated. The shape parameter was lower for tofacitinib in every stratum, with non-overlapping 95% CIs.

The common-window analysis tests whether the TTO contrast depends on the different accrual periods of the drugs; the stratified valid-TTO proportions test whether date availability follows reporter composition or calendar time; and the stratum-specific TTO distributions test whether the contrast depends on reporter or submission mix. The contrast was reproduced under all three restrictions.

Bayesian network edge overlap and bootstrap stability

Bayesian network structures were evaluated using 1,000 bootstrap resamples for each drug. Edge-inclusion frequency was defined as the proportion of bootstrap networks containing the corresponding undirected adjacency. Orientation stability was defined as the proportion of bootstrap networks retaining the same model-derived edge orientation. These measures describe the reproducibility of the learned reporting structure and do not imply temporal or causal relationships.

The Bayesian edge sets were compared with the Ising, phi, and PPMI edge sets using the Jaccard index. Bayesian-specific edges were defined as Bayesian edges that were absent from the union of the three co-occurrence networks. For tofacitinib, 33 of 160 Bayesian edges were Bayesian-specific, with a median edge-inclusion frequency of 0.853. For upadacitinib, 55 of 100 Bayesian edges were Bayesian-specific, with a median edge-inclusion frequency of 0.855. Edge-inclusion and orientation frequencies for the key adjacencies on which the interpretation rests are reported in Table S29; the summary statistics above are computed over all 160 tofacitinib and 100 upadacitinib retained edges.

Table S28. Comparison of Bayesian and co-occurrence network edge sets.

Comparator network	Comparator edges	Shared	Jaccard	Bayesian only	Comparator only
**Tofacitinib (160 Bayesian edges)**					
Ising	256	125	0.430	35	131
phi	369	127	0.316	33	242
PPMI	498	127	0.239	33	371
Union of the three	498	127	0.239	33	371
**Upadacitinib (100 Bayesian edges)**					
Ising	54	40	0.351	60	14
phi	90	45	0.310	55	45
PPMI	104	45	0.283	55	59
Union of the three	106	45	0.280	55	61

Jaccard indices were calculated as the size of the intersection divided by the size of the union of the two edge sets. Bayesian-specific edges were defined as Bayesian edges absent from the union of the Ising, phi, and PPMI edge sets. Bayesian network edges were compared as undirected adjacencies. For tofacitinib the union of the three co-occurrence edge sets coincides with the PPMI edge set, because every Ising and phi adjacency was also present in the PPMI network.

Table S29. Edge-level bootstrap stability of the Bayesian networks: key adjacencies.

Drug	PT1	PT2	Edge freq.	Orient. freq.	BN-only
Tofacitinib	pain	therapy interrupted	1.000	0.906	No
Tofacitinib	drug ineffective	therapy interrupted	1.000	0.717	No
Tofacitinib	condition aggravated	therapy interrupted	1.000	0.542	No
Upadacitinib	fall	therapy interrupted	1.000	0.590	No
Upadacitinib	pain	therapy interrupted	1.000	0.565	No

Edge-inclusion frequency represents the proportion of 1,000 bootstrap networks containing the corresponding adjacency. Orientation stability represents the proportion of bootstrap networks retaining the same model-derived orientation. The orientation of an edge was not interpreted as causal or temporal because FAERS does not record the temporal sequence of adverse events.

Signal-definition sensitivity and report-count analysis

To assess the robustness of the signal definition, we separately evaluated the IC025, ROR, PRR/χ^2, and EBGM05 criteria and examined minimum report-count thresholds of 3, 10, and 30. Counts were calculated after the same exclusion of procedure-related PTs used in the primary analysis. Criterion-specific counts overlap and are therefore not additive.

Within the analysis set of 2,513 tofacitinib and 2,228 upadacitinib PTs, every PT meeting IC025 > 0 also met the ROR criterion. No PT therefore satisfied IC025 > 0 while failing all three supporting criteria, and the composite definition selected exactly the same PTs as IC025 > 0 for both drugs at every report-count threshold. The IC025 requirement was the binding constraint. It excluded 52 tofacitinib and 102 upadacitinib PTs that the ROR criterion alone would have accepted, and 86 and 201 PTs, respectively, that the PRR/χ^2 criterion alone would have accepted; the latter had a median of 4 reports for both drugs. Requiring a two-fold effect size on both PRR and EBGM05, as the all-four-criteria combination does, would instead have removed 145 tofacitinib and 116 upadacitinib composite-positive PTs, with medians of 190 and 75 reports and median RORs of 1.49 and 1.65. The two alternatives thus depart from the composite definition in opposite directions: a frequentist ratio applied alone admits sparsely reported terms whose ratios are unstable, whereas a two-fold effect-size requirement removes the most frequently reported terms, in which the relative increase is moderate but statistically well determined. Symptom and functional PTs fall predominantly in the latter group (Table S30B).

Table S30. Sensitivity of positive PT signals to the components of the composite signal definition.

(A) Counts by criterion and minimum report count.

Drug	Minimum reports	IC025 > 0	ROR	PRR	EBGM05	Primary composite	All four criteria
Tofacitinib	3	316	368	257	57	316	57
Tofacitinib	10	283	304	147	53	283	53
Tofacitinib	30	214	224	78	39	214	39
Upadacitinib	3	413	515	498	145	413	138
Upadacitinib	10	275	294	170	63	275	63
Upadacitinib	30	149	154	56	25	149	25

(B) Criterion-by-criterion detail for the seven symptom and functional PTs of interest.

Drug / PT	Reports	ROR (ROR025)	IC025	PRR (EBGM05)	Criteria met
**Tofacitinib**					
pain	6,413	1.45 (1.41)	0.40	1.41 (1.33)	IC025, ROR
arthralgia	4,392	0.83 (0.81)	−0.27	0.85 (0.83)	None
fatigue	3,452	1.15 (1.11)	0.12	1.14 (1.09)	IC025, ROR
fall	1,862	1.15 (1.09)	0.10	1.15 (1.08)	IC025, ROR
depressed mood	1,039	11.58 (10.63)	2.59	11.39 (6.00)	All four
loss of personal independence in daily activities	828	1.36 (1.27)	0.29	1.36 (1.24)	IC025, ROR
grip strength decreased	94	0.37 (0.30)	−1.69	0.37 (0.32)	None
**Upadacitinib**					
pain	2,783	1.16 (1.11)	0.12	1.15 (1.10)	IC025, ROR
arthralgia	2,418	0.90 (0.86)	−0.21	0.91 (0.87)	None
fall	1,444	1.77 (1.68)	0.67	1.74 (1.60)	IC025, ROR

Drug / PT	Reports	ROR (ROR025)	IC025	PRR (EBGM05)	Criteria met
fatigue	1,023	0.62 (0.58)	−0.74	0.64 (0.61)	None
loss of personal independence in daily activities	446	1.40 (1.27)	0.30	1.40 (1.25)	IC025, ROR
grip strength decreased	197	1.61 (1.39)	0.42	1.62 (1.37)	IC025, ROR
depressed mood	27	0.31 (0.20)	−2.25	0.32 (0.22)	None

Table S31. Report-count distribution and minimum-report-count sensitivity of primary positive PT signals.

Drug	Minimum reports	Positive PT n	Median report count	IQR	Range
Tofacitinib	3	316	66.5	21–285	3–7,360
Tofacitinib	10	283	81	31–299	10–7,360
Tofacitinib	30	214	162	65–492	30–7,360
Upadacitinib	3	413	17	7–53	3–2,783
Upadacitinib	10	275	34	17–95	10–2,783
Upadacitinib	30	149	86	49–165	30–2,783

Primary composite denotes IC025 > 0 plus at least one of the ROR, PRR/χ^2, or EBGM05 criteria. Criterion-specific counts are overlapping and should not be summed. In panel A the primary composite column is identical to the IC025 > 0 column at every threshold, because every PT meeting IC025 > 0 also met the ROR criterion. In panel B, values in parentheses are the lower confidence or credible limits, ROR025 and EBGM05. χ^2 exceeded 4 for every PT listed, so the PRR criterion was determined by the PRR value alone. Arthralgia is shown as a reference term because it is common in RA irrespective of treatment; it did not meet the positive-signal criterion for either drug.

Methotrexate Sensitivity Analysis

A methotrexate comparator cohort was constructed from FAERS reports recorded during the same observation period as the primary analyses. Reports were included when methotrexate was the primary suspect drug and rheumatoid arthritis was recorded as the indication. Drug names and adverse events were standardized using the same procedures as in the primary analyses. Duplicate and follow-up reports were handled using the same case-level rules, and procedure-related PTs were excluded using the same prespecified rule. The same composite positive-signal criterion was applied: at least three reports, IC025 > 0, and at least one of ROR025 > 1, PRR ≥ 2 with $\chi^2 \geq 4$, or EBGM05 > 2. The final methotrexate comparator cohort comprised 37,526 unique reports.

Candidate network nodes were selected using a frequency-based network inclusion procedure of the same form as in the primary analyses. Ising, phi, and PPMI networks were estimated using the same implementation parameters. Because raw degree counts are not directly comparable across networks with different node numbers and densities, degree was standardized within each network as $z = (d_i - \bar{d}) / SD(d)$ where d_i is the degree of node i. Empirical percentiles were calculated from the within-network degree distribution. These standardized values describe relative centrality within each network and do not represent effect sizes or causal evidence. Two distinct rules could exclude a PT from a node set, and they carry different meanings. A PT could fail the composite positive-signal criterion, which indicates that its reporting was not disproportionate relative to other rheumatoid arthritis reports, or it could meet that criterion and fall below the frequency threshold applied to network construction, which indicates only that it was too infrequent for stable pairwise estimation. In the comparator cohort, the frequency threshold retained 79 nodes, the least frequent of which was reported 294 times; decreased grip strength illustrates the second rule, since it met the composite criterion with 189 reports and an ROR of 1.21 but was not retained. Supplementary Table S32 records which of the two rules applied to each term discussed. A PT labelled "no signal" was therefore not necessarily absent from the reports, and "no signal" and "below threshold" should not be read as equivalent.

The methotrexate comparator cohort comprised 37,526 unique reports. The composite criterion identified 1,263 positive PT signals, of which 79 met the network inclusion rule. The Ising, phi, and PPMI networks contained 485, 1,538, and 1,640 edges, with densities of 0.157, 0.499, and 0.532, respectively (Table S32). The methotrexate networks were mainly organized around joint and inflammatory terms, including joint swelling, synovitis, peripheral swelling, arthropathy, and related disease-activity concepts.

Density-corrected centrality analysis indicated that fatigue remained highly central in the tofacitinib networks (99th–100th percentiles) but exhibited less consistent centrality in the methotrexate networks (57th–94th percentiles). Pain and fall did not meet the positive-signal criterion in the comparator cohort; although decreased grip strength met the criterion, it fell below the network frequency threshold. Loss of personal independence appeared in both analyses but demonstrated lower and less consistent centrality in the methotrexate comparator (Table S32). These findings suggest partial overlap for selected reporting domains but do not reproduce the fall-centred functional configuration observed for upadacitinib.

Table S32. Methotrexate comparator cohort, focus preferred terms, and density-corrected centrality within each network.

Adverse event (PT)	Network (drug)	Method	N nodes	Density	Degree	Z-score	Percentile
Fatigue	Tofacitinib	Ising	77	0.087	21	+2.66	99%
	Tofacitinib	Phi	77	0.126	30	+2.88	100%
	Tofacitinib	PPMI	77	0.170	40	+3.08	100%
	Upadacitinib	Ising	65	0.026	—	no signal	—
	Upadacitinib	Phi	65	0.043	—	no signal	—
	Upadacitinib	PPMI	65	0.050	—	no signal	—
	Methotrexate (comparator)	Ising	79	0.157	23	+1.74	94%
	Methotrexate (comparator)	Phi	79	0.499	51	+0.66	66%
	Methotrexate (comparator)	PPMI	79	0.532	52	+0.57	57%
Fall	Tofacitinib	Ising	77	0.087	8	+0.25	66%
	Tofacitinib	Phi	77	0.126	10	+0.06	56%
	Tofacitinib	PPMI	77	0.170	14	+0.12	64%
	Upadacitinib	Ising	65	0.026	11	+4.19	100%
	Upadacitinib	Phi	65	0.043	12	+2.83	100%
	Upadacitinib	PPMI	65	0.050	12	+2.32	97%
	Methotrexate (comparator)	Ising	79	0.157	—	no signal	—
	Methotrexate (comparator)	Phi	79	0.499	—	no signal	—
	Methotrexate (comparator)	PPMI	79	0.532	—	no signal	—
Pain	Tofacitinib	Ising	77	0.087	21	+2.66	99%

Adverse event (PT)	Network (drug)	Method	N nodes	Density	Degree	Z-score	Percentile
	Tofacitinib	Phi	77	0.126	28	+2.60	99%
	Tofacitinib	PPMI	77	0.170	30	+1.94	99%
	Upadacitinib	Ising	65	0.026	4	+1.05	87%
	Upadacitinib	Phi	65	0.043	8	+1.61	88%
	Upadacitinib	PPMI	65	0.050	12	+2.32	97%
	Methotrexate (comparator)	Ising	79	0.157	—	no signal	—
	Methotrexate (comparator)	Phi	79	0.499	—	no signal	—
	Methotrexate (comparator)	PPMI	79	0.532	—	no signal	—
Grip strength decreased	Tofacitinib	Ising	77	0.087	—	no signal	—
	Tofacitinib	Phi	77	0.126	—	no signal	—
	Tofacitinib	PPMI	77	0.170	—	no signal	—
	Upadacitinib	Ising	65	0.026	3	+0.60	81%
	Upadacitinib	Phi	65	0.043	9	+1.91	92%
	Upadacitinib	PPMI	65	0.050	10	+1.79	90%
	Methotrexate (comparator)	Ising	79	0.157	—	no signal	—
	Methotrexate (comparator)	Phi	79	0.499	—	no signal	—
	Methotrexate (comparator)	PPMI	79	0.532	—	no signal	—
Loss of personal independence in daily activities	Tofacitinib	Ising	77	0.087	8	+0.25	66%
	Tofacitinib	Phi	77	0.126	14	+0.62	74%
	Tofacitinib	PPMI	77	0.170	20	+0.80	77%
	Upadacitinib	Ising	65	0.026	5	+1.50	91%
	Upadacitinib	Phi	65	0.043	8	+1.61	88%
	Upadacitinib	PPMI	65	0.050	8	+1.27	85%
	Methotrexate (comparator)	Ising	79	0.157	17	+0.77	81%
	Methotrexate (comparator)	Phi	79	0.499	47	+0.44	46%
	Methotrexate (comparator)	PPMI	79	0.532	50	+0.46	47%

" No signal" indicates that the preferred term was evaluated in that cohort but did not meet the composite positive-signal criterion; "below threshold" indicates that the term met the criterion but was reported too infrequently to be retained as a network node. Neither designation implies absence from the reports. Arthralgia was evaluated in all three cohorts but met the criterion in none (2,796 methotrexate, 4,392 tofacitinib, and 2,418 upadacitinib reports; ROR 0.81, 0.83, and 0.90).

Reporter- and Submission-Stratified Network Analysis

The reporter and submission-type strata defined above were used to re-estimate the co-occurrence networks while holding each drug's main-analysis node set fixed, ensuring that only the study population varied across strata.

Table S33. Reporter- and submission-stratified co-occurrence networks.

(A) Network properties within each stratum

Drug / Stratum	Reports	Nodes	Method	Edges	Density	Modularity	Communities	Most connected node (degree)
**Tofacitinib**								
All reports	57,438	77	Ising	256	0.0875	0.606	17	fatigue (21)
All reports	57,438	77	Phi	369	0.1261	0.486	12	fatigue (30)
All reports	57,438	77	PPMI	490	0.1675	0.427	9	fatigue (39)
Health professional	25,104	77	Ising	142	0.0485	0.694	27	pain (14)
Health professional	25,104	77	Phi	257	0.0878	0.514	18	fatigue (25)
Health professional	25,104	77	PPMI	358	0.1224	0.454	16	fatigue (35)
Consumer	24,063	77	Ising	154	0.0526	0.657	25	fatigue (17)
Consumer	24,063	77	Phi	196	0.0670	0.573	19	fatigue (17)
Consumer	24,063	77	PPMI	265	0.0906	0.542	14	pain (23)
Expedited	21,421	77	Ising	173	0.0591	0.609	24	fatigue (28)
Expedited	21,421	77	Phi	336	0.1148	0.412	15	fatigue (38)
Expedited	21,421	77	PPMI	460	0.1572	0.367	11	fatigue (41)
Non-expedited	36,017	77	Ising	156	0.0533	0.694	24	pain (16)
Non-expedited	36,017	77	Phi	208	0.0711	0.601	19	pain (19)
Non-expedited	36,017	77	PPMI	260	0.0889	0.583	14	pain (23)
**Upadacitinib**								
All reports	29,869	65	Ising	54	0.0260	0.674	34	fall (11)
All reports	29,869	65	Phi	90	0.0433	0.582	30	fall (12)
All reports	29,869	65	PPMI	103	0.0495	0.607	27	back pain (12)
Health professional	5,369	61	Ising	5	0.0027	0.669	57	pain (2)

Drug / Stratum	Reports	Nodes	Method	Edges	Density	Modularity	Communities	Most connected node (degree)
Health professional	5,369	61	Phi	30	0.0164	0.561	41	musculoskeletal disorder (6)
Health professional	5,369	61	PPMI	12	0.0066	0.562	51	pain (5)
Consumer	21,645	65	Ising	50	0.0240	0.726	35	fall (11)
Consumer	21,645	65	Phi	69	0.0332	0.656	32	fall (12)
Consumer	21,645	65	PPMI	79	0.0380	0.676	31	fall (12)
Expedited	20,881	63	Ising	49	0.0251	0.691	34	fall (11)
Expedited	20,881	63	Phi	73	0.0374	0.604	31	fall (12)
Expedited	20,881	63	PPMI	86	0.0440	0.612	28	fall (12)
Non-expedited	8,988	64	Ising	18	0.0089	0.610	53	musculoskeletal stiffness (6)
Non-expedited	8,988	64	Phi	57	0.0283	0.530	35	musculoskeletal stiffness (9)
Non-expedited	8,988	64	PPMI	35	0.0174	0.507	47	grip strength decreased (8)

(B) Structural concordance between each stratum and the corresponding all-report network

Drug / Stratum	Method	Shared nodes	Edge Jaccard	Community purity
**Tofacitinib**				
Health professional	Ising	77	0.485	0.831
Health professional	Phi	77	0.647	0.883
Health professional	PPMI	77	0.631	0.812
Consumer	Ising	77	0.565	0.825
Consumer	Phi	77	0.511	0.896
Consumer	PPMI	77	0.519	0.747
Expedited	Ising	77	0.554	0.864
Expedited	Phi	77	0.613	0.844
Expedited	PPMI	77	0.610	0.753
Non-expedited	Ising	77	0.567	0.851
Non-expedited	Phi	77	0.547	0.844

Drug / Stratum	Method	Shared nodes	Edge Jaccard	Community purity
Non-expedited	PPMI	77	0.503	0.727
**Upadacitinib**				
Health professional	Ising	61	0.078	0.795
Health professional	Phi	61	0.231	0.803
Health professional	PPMI	61	0.130	0.770
Consumer	Ising	65	0.825	0.992
Consumer	Phi	65	0.710	0.954
Consumer	PPMI	65	0.670	0.923
Expedited	Ising	63	0.839	0.976
Expedited	Phi	63	0.798	1.000
Expedited	PPMI	63	0.796	0.937
Non-expedited	Ising	64	0.309	0.820
Non-expedited	Phi	64	0.374	0.836
Non-expedited	PPMI	64	0.314	0.805

Networks were re-estimated within each reporter and submission-type stratum using each drug's main-analysis node set, so that only the study population varied across strata. Nodes indicate the number of preferred terms estimable within the stratum. Community purity is the symmetric mean overlap between the community assignments of the stratified and the all-report network. Reporter groups were defined by the qualification of the primary reporter, and submission type was derived from the report type field; strata with fewer than 300 reports were not estimated.

Table S34. Cross-drug structural comparison within each reporter and submission-type stratum.

Stratum	Method	Shared nodes	Edge Jaccard (tofacitinib vs upadacitinib)	Community purity
All reports	Ising	25	0.323	0.820
All reports	Phi	25	0.442	0.840
All reports	PPMI	25	0.400	0.900
Health professional	Ising	25	0.200	0.820
Health professional	Phi	25	0.222	0.760
Health professional	PPMI	25	0.118	0.780
Consumer	Ising	25	0.400	0.840
Consumer	Phi	25	0.333	0.840
Consumer	PPMI	25	0.333	0.800
Expedited	Ising	23	0.421	0.826
Expedited	Phi	23	0.429	0.826
Expedited	PPMI	23	0.432	0.826
Non-expedited	Ising	25	0.368	0.840
Non-expedited	Phi	25	0.346	0.820
Non-expedited	PPMI	25	0.393	0.760

Comparisons were restricted to preferred terms present in both drugs' node sets within the stratum. Edge Jaccard values below 0.50 in every stratum indicate that the co-occurrence structures of the two drugs remained distinguishable irrespective of reporter or submission type.
